# *SOCS5* knockdown suppresses metastasis of hepatocellular carcinoma by ameliorating HIF-1α-dependent mitochondrial damage

**DOI:** 10.1038/s41419-022-05361-z

**Published:** 2022-11-01

**Authors:** Dingan Luo, Youpeng Wang, Mao Zhang, Haoran Li, Deze Zhao, Hui Li, Xiaowu Chen, Cheng Jin, Bing Han

**Affiliations:** 1 Department of Hepatobiliary and Pancreatic Surgery, Affiliated Hospital of Qingdao University, Qingdao, China; 2 Department of Thoracic Surgery, Xiangya Hospital, Central South University, Changsha, Hunan China; 3 Peking University Health Science center, Peking University, Beijing, China; 4 Department of Surgery, Asian Liver Center, Stanford University School of Medicine, Stanford, CA USA; 5 Institute of Medical Robotics, School of Biomedical Engineering, Shanghai Jiao Tong University, Shanghai, China

**Keywords:** Cancer microenvironment, Cancer

## Abstract

The Pringle maneuver (PM) is widely used during hepatocellular carcinoma (HCC) resection. However, it inevitably leads to ischemia and hypoxia, which promotes tumor metastasis. In this study, immunohistochemical staining of specimens from 130 HCC patients revealed that long-time PM significantly affected the prognosis of patients with high expression of suppressor of cytokine signaling 5 (*SOCS5*), but did not affect the prognosis of patients with low expression of *SOCS5*. The TCGA database showed that patients with high expression of *SOCS5* had higher hypoxia scores, and it was proved that *SOCS5* could promote the expression of hypoxia-inducible factor 1 subunit alpha (*HIF-1α*) protein by clinical tissue samples, cell experiments, lung metastases, and subcutaneous tumorigenesis experiments. Then, we used CoCl2 to construct a hypoxia model, and confirmed that *SOCS5* knockdown resisted hypoxia-induced mitochondrial damage by inhibiting the expression of *HIF-1α*, thereby inhibiting the invasion and migration of HCC cells by immunofluorescence, electron microscopy, migration, invasion, and other experiments. We performed rescue experiments using LY294002 and rapamycin and confirmed that the knockdown of SOCS5-inhibited HCC cell invasion and migration by inhibiting the *PI3K/Akt/mTOR/HIF-1α* signaling axis. More importantly, we obtained consistent conclusions from clinical, cellular, and animal studies that the hypoxia-induced invasion and migration ability of *SOCS5*-inhibited HCC were weaker than that of normal HCC. In conclusion, we identified a novel role for *SOCS5* in regulating *HIF-1α*-dependent mitochondrial damage and metastasis through the *PI3K/Akt/mTOR* pathway. The development of a *SOCS5*-specific inhibitor, an indirect inhibitor of *HIF-1α*, might be effective at controlling PM-induced tumor micrometastases during HCC resection.

## Introduction

Hepatocellular carcinoma (HCC) is a highly aggressive form of primary liver cancer, which is the third leading cause of cancer-related deaths [1]. Surgical resection is the primary treatment for patients with HCC [2], and the Pringle maneuver (PM) is widely used during liver resection because of its ability to greatly reduce the risk of bleeding [3]. However, this inevitably leads to ischemia and hypoxia in HCC, which promotes tumor invasion and metastasis by activating related signaling pathways [4, 5]. Therefore, deciphering the hypoxia-mediated mechanisms of HCC would be beneficial.

Hypoxia-inducible factor 1 subunit alpha (HIF-1α) participates in the hypoxic response of HCC. It activates various genes as transcription factors. These target genes are involved in multiple aspects of tumorigenesis, including metabolism, angiogenesis, proliferation, invasion, and metastasis [6–8]. Therefore, activating the HIF-1α pathway is related to tumor invasion and poor clinical prognosis. HIF-1α inhibitors are warranted for cancer treatment regimens; however, no specific HIF-1α inhibitor has gained clinical approval to date due to complex upstream regulation and intertwined mechanisms. Therefore, it is necessary to study the molecular mechanisms underlying HIF-1α regulation and develop more accurate anticancer therapies.

Although HIF-1α activates hypoxia-induced genes, it is easily degraded in the cytoplasm by von Hippel-Lindau (VHL)-containing E3 ubiquitin ligase [9]. Therefore, additional mechanisms must be coordinated to ensure the stability of HIF-1α, such that it may act as a transcription factor. Growth factors, cytokines, and other signaling molecules tend to accumulate with the HIF-1α protein in cells [10–12]. This is seen in the PI3K/Akt/mTOR pathway, whose activation mediates the translation of HIF-1α proteins by phosphorylating eukaryotic translation initiation factor 4E binding protein 1 and p70 S6 kinase [13]. Previously, we found that the suppressor of cytokine signaling 5 (SOCS5) can activate PI3K/Akt/mTOR signaling pathway [14]. However, little is known about the molecular mechanisms of SOCS5 in hypoxia-related HCC invasion and metastasis.

In this study, we found that HCC patients with low SOCS5 expression were more tolerant to hypoxia resulting from the PM. We studied the role of SOCS5 in hypoxia-induced invasion and metastasis of hepatocellular carcinoma in vitro and in vivo experiments. We also studied the role of SOCS5 in hypoxia-induced mitochondrial damage. Crucially, we explored the role of SOCS5 in hypoxia-induced HCC tumor metastasis in metastatic human HCC orthotopic nude mouse models using left hepatic artery and vein ligation (LHAVL) to simulate PM during surgery.

## Materials and methods

### Patient samples

Between January 2013 and December 2014, 130 patients with HCC were recruited from the Affiliated Hospital of Qingdao University (Table 1). The informed consent of all subjects was obtained. All patients met these inclusion criteria [15]: age ≥18 years, Child-Pugh grade A, HCC confirmed by histopathology, no hilar lymph nodal involvement or extrahepatic metastases, clinical diagnosis of resectable HCC, and an indication for curative resection of primary HCC including single or multiple tumors. The study protocol was in accordance with the ethical guidelines of the 1975 Declaration of Helsinki (revised in 2013). This study was approved by the Ethics Committee of the Affiliated Hospital of Qingdao University. The Pringle maneuver was defined as the continuous or intermittent tightening of a rubber tube around the entire hepatoduodenal ligament. Typically, when the estimated Pringle maneuver time exceeded 20 min, the procedure was carried out for 15 min, perfused for 5 min, and repeated as needed. The total ischemic time was obtained by adding all clamp times during surgery.

**Table 1:** Comparison of clinical characteristics in patients with the pringle maneuver time less than or over 15 min*.

Characteristics	Pringle maneuver time <15 min	Pringle maneuver time ≥15 min	*P* value
*N*	81 (62.3)	49 (37.7)	
Age, years			0.505^a^
≤60	59 (72.8)	33 (67.3)	
>60	22 (27.2)	16 (32.7)	
Sex			0.152^a^
Male	65 (80.2)	44 (89.8)	
Female	16 (19.8)	5 (10.2)	
HBsAg			1^b^
Negative	6 (7.4)	4 (8.2)	
Positive	75 (92.6)	45 (91.8)	
Cirrhosis			0.766^a^
Yes	63 (77.8)	37 (75.5)	
No	18 (22.2)	12 (24.5)	
AFP, ng/ml			0.832^a^
≤400	56 (69.1)	33 (67.3)	
>400	25 (30.9)	16 (32.7)	
Tumor size, cm	4 (2.75–5)	5 (3.25–6)	**0.032**^**d**^
Tumor number			**0.027**^**a**^
Solidary	74 (91.4)	38 (77.6)	
Multiple	7 (8.6)	11 (22.4)	
Microvascular invasion			r0.461^a^
Yes	20 (24.7)	15 (30.6)	
No	61 (75.3)	34 (69.4)	
Macroscopic vascular invasion			0.541^b^
Yes	6 (7.4)	6 (12.2)	
No	75 (92.6)	43 (87.8)	
Tumor capsule			0.126^b^
Yes	4 (4.9)	7 (14.3)	
No	77 (95.1)	42 (85.7)	
Tumor differentiation			0.751^a^
I-II	71 (87.7)	42 (85.7)	
III-IV	10 (12.3)	7 (14.3)	
Steatosis			1^c^
Yes	4 (4.9)	2 (4.1)	
No	77 (95.1)	47 (95.9)	
SOCS5 expression			0.62^a^
Low	41 (50.6)	27 (55.1)	
High	40 (49.4)	22 (44.9)	
Blood loss, ml	200 (100–300)	200 (150–300)	0.574^d^
Blood transfusion			0.717^b^
Yes	7 (8.6)	6 (12.2)	
No	74 (91.4)	43 (87.8)	

*AFP* α-fetoprotein, *SOCS5* suppressor of cytokine signaling 5.

^*^Continuous variables were presented as median (Q1–Q3) and tested by the Mann–Whitney *U*-test. Categorical variables were expressed as numbers (%) and tested by the χ^2^ test, continuity correction by χ^2^ test, or Fisher’s exact test.

^a^Pearson’s χ^2^ test.

^b^Continuity correction by χ^2^ test.

^c^Fisher exact test.

^d^Mann–Whitney *U*-test.

*P* values that are statistically significant are shown in bold.

### Immunohistochemistry

SOCS5 protein expression in 130 paraffin-embedded liver cancer tissues was detected using immunohistochemistry (IHC). The IHC staining score was based on staining intensity (0 = negative, 1 = weak, 2 = medium, or 3 = strong) and the percentage of positively stained cells in the observation field (0 = 0%, 1 = 1–25%, 2 = 26–50%, and 3 ≥ 51%). The final stained fraction was determined by multiplying the intensity and percentage fractions, ranging from 0–9. Low and high expression were defined as final scores of <4 and 4–9, respectively. In addition, 64 paired tumorous and adjacent non-tumorous liver tissues from HCC patients were obtained from the Shanghai Outdo Biotech (Shanghai, China). SOCS5 and HIF-1α protein expression in 64 paraffin-embedded liver cancer tissues were detected using IHC.

### Cell culture

The liver cancer cell lines (HepG2, Huh7, PLC/PRF/5, MHCC97H, Hep3B, and HCCLM3; contains STR profiling, without mycoplasma contamination) were purchased from a cell bank at the Chinese Academy of Sciences (Shanghai, China). HepG2 cells were cultured in minimum essential medium with 10% fetal bovine serum (FBS) and 1% penicillin/streptomycin (P/S). Huh7, PLC/PRF/5, MHCC97H, Hep3B, and HCCLM3 cells were cultured in Dulbecco’s modified Eagle’s medium supplemented with 10% FBS and 1% P/S. All cells were maintained in a humidified atmosphere with 5% CO_2_ at 37 °C.

### Transfections and cell treatments

Details of the transfection are presented in our previous study [14]. At 6 h post-transfection, cells were treated with 100–300 µM CoCl_2_ (Sigma-Aldrich) for 24 h to simulate hypoxia [16, 17], 20 µg/ml LY294002 (Absin, Shanghai, China) for 48 h to inhibit PI3K [18], and 10 µM rapamycin (MCE) for 48 h to inhibit mTOR [18].

### Immunofluorescence

Immunofluorescence staining was performed on the cells cultured on coverslips. Cells were fixed in 4% paraformaldehyde for 20 min at 37 °C. Intracellular epitope detection was performed in cells permeabilized with 0.1% TritonX-100 in PBS for 5 or 15 min. Following blocking with 5% bovine serum albumin (BSA) for 30 min, cells were exposed overnight at 4 °C to antibodies for SOCS5 (sc-100858, Santa Cruz Biotechnology, Dallas, TX, USA), E-cadherin (#3195, Cell Signaling Technology, Danvers, MA, USA), and HIF-1α (#36169, CST). Primary antibodies were detected using antirabbit IgG (#4413, CST) or anti-mouse IgG (abs20003, Absin) as secondary antibodies. 4′,6-Diamidino-2-phenylindole (DAPI) was used to counterstain the nuclei. F-actin was stained with phalloidin (CA1610, Solarbio, Beijing, China), according to the manufacturer’s instructions.

### Mitochondrial membrane potential and reactive oxygen species analysis

To determine the changes in mitochondrial membrane potential (MMP), JC-1 dye (C2006, Beyotime, Shanghai, China) was used in situ, per the manufacturer’s instructions. Intracellular ROS levels were evaluated using a Reactive Oxygen Species Assay Kit (S0033S, Beyotime) per the manufacturer’s instructions. Images were obtained using a fluorescence microscope.

### MitoTracker Green and LysoTracker Red staining

Cells in different treatment groups were collected and then incubated with MitoTracker Green (C1048, Beyotime) and LysoTracker Red (C1046; Beyotime) at 37 °C for 30 min in the dark. Images were obtained using a fluorescence microscope.

### Animal experiments

The operations and rearing of nude mice were performed in strict accordance with the principles approved by the Committee on the Ethics of Animal Experiments of Qingdao University. Twenty-four male BALB/c nude mice (4–6 weeks old) were purchased from Beijing Vital River Laboratory Animal Technology (Beijing, China) and randomly divided into two groups. Mice were injected with 2 × 10^6^ HCCLM3^LV-shNC^ cells or 2 × 10^6^ HCCLM3^LV-shSOCS5^ cells through the tail veins. After 4 weeks, all mice were sacrificed, and the lungs were excised and embedded in paraffin for IHC. Meanwhile, twelve mice from each group were subcutaneously injected at one site. The tumor onset was measured with calipers at the injection site weekly. The animals were sacrificed 28 days after injection. The tumors were excised and embedded in paraffin for IHC.

The construction of the orthotopic HCC model was performed using male BALB/c nude mice from 6–8 weeks old (about 25 g). A microinjector was used to inject 1,000,000 HCCLM3 human HCC cells in 30 μL PBS mixed with 30 μL Matrigel (BD Biosciences, United States) into the left lobe of the liver. Care was taken to ensure that no HCCLM3 cells were disseminated into the peritoneum. The needle was inserted 2–3 mm on the liver surface, and it was withdrawn to confirm that it was not in the blood vessel. Slowly inject, stay for 1 min after injection, rotate the needle to exit, avoid cell backflow, and white protrusions can generally be seen in the liver after successful injection. After needle withdrawal, quickly use a sterile cotton swab to compress the needle inlet for 30 s until there is no bleeding, and then remove the cotton swab. After the injection of tumor cells, the incision was closed with a surgical suture. Three weeks after the operation, all mice were randomly divided into each experimental group. We used LHAVL instead of PM to induce hypoxia. Sham-operated mice underwent laparotomy with exposure of the liver and dissection of the vascular structures, but without interruption of the hepatic blood flow. Then LHAVL was performed in an orthotopic HCC model overexpressing SOCS5, HCC tumors in the left lobe of the liver were collected 2 h later, and the blocking was released 40 min after LHAVL [19]. Then, we implanted SOCS5 knockdown HCCLM3 in the left lobe of the liver of nude mice, and constructed an orthotopic HCC model with SOCS5 knockdown. Then LHAVL was performed intermittently, blocking for 4 min, opening for 2 min, and then releasing the blocking after 4 min of blocking again (Anesthesia was induced by isoflurane 5 minimum alveolar concentration (mac), and when LHAVL was performed, it was adjusted to 1 mac to maintain anesthesia). After 3 weeks, the liver and lungs of nude mice were collected.

### Other basic experiments

Details of lentivirus construction and infection of cell lines, RNA extraction and quantitative real-time PCR, western blotting analysis, cell migration, and invasion assays, wound-healing assay, and electron microscopy are presented in our previous study [14]. The gray values of the three repeated bands of all western blot experiments in this study are shown in the attached table.

### Statistical analysis

All statistical analyses were performed using GraphPad Prism 8.0. Categorical variables were expressed as numbers (%) and tested using the *χ*^2^ test, continuity correction by *χ*^2^ test, or Fisher’s exact test. Continuous variables were presented as mean ± SD or median (Q1–Q3) and tested using the *t*-test or Mann–Whitney *U*-test. Survival curves were plotted using the Kaplan–Meier method and compared using the log-rank test. Statistical significance was set at *p* < 0.05.

## Results

### The role of SOCS5 in the HCC hypoxic microenvironment may depend on HIF-1α regulation

**Fig. 1:**
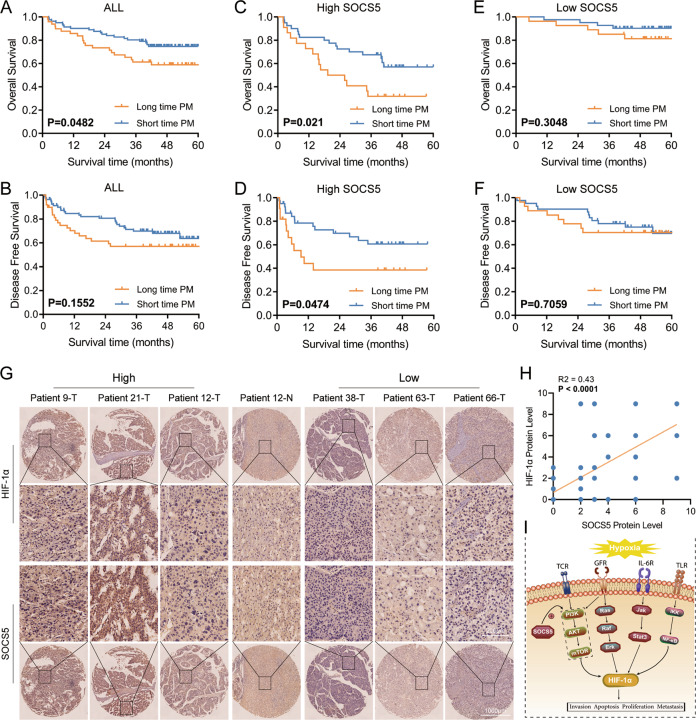
The role of SOCS5 in the HCC hypoxic microenvironment might depend on the regulation of HIF-1α. Kaplan–Meier overall survival (**A**) and disease-free survival (**B**) curves for all 130 patients with HCC stratified by long and short time Pringle maneuver. Kaplan–Meier overall survival (**C**) and disease-free survival (**D**) curves for 68 patients with high SOCS5 expression stratified by long and short time Pringle maneuver. Kaplan–Meier overall survival (**E**) and disease-free survival (**F**) curves for 62 patients with low SOCS5 expression stratified by long and short time Pringle maneuver. **G**, **H** Immunohistochemical analysis of SOCS5 and HIF-1α protein expression in human HCC tissues. **I** Schematic diagram of SOCS5 regulating HIF-1α in the hypoxic microenvironment of HCC. T tumor, N normal.

We divided 130 patients into two groups (power = 0.85): long-term PM (≥15 min) and short-term PM (<15 min), according to the time taken during radical resection of HCC [15]. Patients who received long PM had larger tumors (*p* = 0.032) and more tumors (*p* = 0.027) (Table 1). We found that patients with long-term PM exhibited worse overall survival (OS) than those with short-term PM (Fig. 1A), while there was no obvious difference in disease-free survival (DFS) (Fig. 1B). To explore the role of SOCS5 in PM, we examined its expression in 130 samples using IHC. Clinicopathological and surgical characteristics of long-term PM and short-term PM were comparable in patients with low (Table 2) or high (Table 3) expression of SOCS5. Interestingly, we found that PM duration significantly affected the survival (Fig. 1C) and recurrence (Fig. 1D) of patients with high SOCS5 expression, but did not affect survival (Fig. 1E) or recurrence (Fig. 1F) in patients with low SOCS5 expression. HCC patients with low expression of SOCS5 may be more tolerant to PM-induced changes in the hypoxic tumor microenvironment.

**Table 2:** Comparison of clinical characteristics in low SOCS5 patients with the pringle maneuver time less than or over 15 min*.

Characteristics	Pringle maneuver time <15 min	Pringle maneuver time ≥15 min	*p* value
*N*	41 (60.3)	27 (39.7)	
Age, years			0.565^a^
≤60	30 (73.2)	18 (66.7)	
>60	11 (26.8)	9 (33.3)	
Sex			0.957^b^
Male	35 (85.4)	24 (88.9)	
Female	6 (14.6)	3 (11.1)	
HBsAg			0.423^c^
Negative	3 (7.3)	4 (14.8)	
Positive	38 (92.7)	23 (85.2)	
Cirrhosis			0.633^b^
Yes	35 (85.4)	21 (77.8)	
No	6 (14.6)	6 (22.2)	
AFP, ng/ml			0.668^a^
≤400	30 (73.2)	21(77.8)	
>400	11 (26.8)	6 (22.2)	
Tumor size, cm	4 (2.75–5)	4 (3–6)	0.182^d^
Tumor number			0.25^c^
Solidary	38 (92.7)	22 (81.5)	
Multiple	3 (7.3)	5 (18.5)	
Microvascular invasion			0.069^a^
Yes	6 (14.6)	9 (33.3)	
No	35 (85.4)	18 (66.7)	
Macroscopic vascular invasion			0.514^c^
Yes	2 (4.9)	0 (0.0)	
No	39 (95.1)	27 (1.0)	
Tumor capsule			0.558^c^
Yes	1 (2.4)	2 (7.4)	
No	40 (97.6)	25(92.6)	
Tumor differentiation			1^b^
I-II	36 (87.8)	23 (85.2)	
III-IV	5 (12.2)	4 (14.8)	
Steatosis			1^c^
Yes	2 (4.9)	1 (3.7)	
No	39 (95.1)	26 (96.3)	
Blood loss, ml	200 (100–300)	200 (100–200)	0.321^d^
Blood transfusion			1^c^
Yes	3 (7.3)	2 (7.4)	
No	38 (92.7)	25 (92.6)	

*AFP* α-fetoprotein, *SOCS5* suppressor of cytokine signaling 5.

^*^Continuous variables were presented as median (Q1–Q3) and tested by the Mann–Whitney *U*-test. Categorical variables were expressed as numbers (%) and tested by the *χ*^2^ test, continuity correction by *χ*^2^ test, or Fisher’s exact test.

^a^Pearson’s *χ*^2^ test.

^b^Continuity correction by *χ*^2^ test.

^c^Fisher exact test.

^d^Mann–Whitney *U*-test.

**Table 3:** Comparison of clinical characteristics in high SOCS5 patients with the pringle maneuver time less than or over 15 min*.

Characteristics	Pringle maneuver time <15 min	Pringle maneuver time ≥15 min	*p* value
*N*	40 (64.5)	22 (35.4)	
Age, years			0.72^a^
≤60	29 (72.5)	15 (68.2)	
>60	11 (27.5)	7 (31.8)	
Sex			0.238^b^
Male	30 (75.0)	20 (90.9)	
Female	10 (25.0)	2 (9.1)	
HBsAg			0.546^c^
Negative	39 (7.5)	0 (0.0)	
Positive	37 (92.5)	22 (1.0)	
Cirrhosis			0.821^a^
Yes	28 (70.0)	16 (72.7)	
No	12 (30.0)	6(27.3)	
AFP, ng/ml			0.419^a^
≤400	26 (65.0)	12 (54.5)	
>400	14 (35.0)	10 (45.5)	
Tumor size, cm	4 (2.63–6.38)	5.88 (33.88–7.13)	0.068^d^
Tumor number			0.159^b^
Solidary	36 (90.0)	16 (72.7)	
Multiple	4 (10.0)	6 (27.3)	
Microvascular invasion			0.533^a^
Yes	14 (35.0)	6 (27.3)	
No	26 (65.0)	16 (72.7)	
Macroscopic vascular invasion			0.159^b^
Yes	4 (10.0)	6 (27.3)	
No	36 (90.0)	16 (72.7)	
Tumor capsule			0.188^b^
Yes	3 (7.5)	5 (22.7)	
No	37 (92.5)	17 (77.3)	
Tumor differentiation			1^b^
I-II	35 (87.5)	19 (86.4)	
III-IV	5 (12.5)	3 (13.6)	
Steatosis			1^c^
Yes	2 (5.0)	1(4.5)	
No	38 (95.0)	21 (95.5)	
Blood loss, ml	200 (100–375)	200 (200–550)	0.057^d^
Blood transfusion			0.601^b^
Yes	4 (10.0)	4 (18.2)	
No	36 (90.0)	18 (81.8)	

*AFP* α-fetoprotein, *SOCS5* suppressor of cytokine signaling 5.

^*^Continuous variables were presented as median (Q1–Q3) and tested by the Mann–Whitney *U*-test. Categorical variables were expressed as numbers (%) and tested by the *χ*^2^ test, continuity correction by *χ*^2^ test, or Fisher’s exact test.

^a^Pearson’s *χ*^2^ test.

^b^Continuity correction by *χ*^2^ test.

^c^Fisher exact test.

^d^Mann–Whitney *U*-test.

To further explore the role of SOCS5 in the hypoxic tumor microenvironment, the high and low expression of SOCS5 were divided according to the median value of *SOCS5* mRNA expression in the TCGA database, and hypoxia scores of HCC patients were calculated using the hypoxia score formula of Ragnum [20], Elvidge [21], and Seigneuric [22]. We found that the hypoxia scores of HCC patients with high SOCS5 expression were higher than those with low SOCS5 expression (Supplementary Fig. S1A–C). We also found a positive relationship between SOCS5 and HIF-1α, the core molecule of the hypoxic tumor microenvironment, at the RNA and protein levels via the cBioPortal website (Supplementary Fig. S1D) and the Human Protein Atlas website (Supplementary Fig. S1E). Critically, we detected the protein expression of SOCS5 and HIF-1α in 64 HCC tissues by IHC using TMA. We found SOCS5 protein levels were positively correlated with HIF-1α protein levels in HCC tissues (Fig. 1G, H). Considering these data and existing knowledge on HIF1A, we hypothesized that SOCS5 promotes the expression of HIF1A and affects the hypoxic tumor microenvironment (Fig. 1I).

### SOCS5 positively regulates the protein expression of HIF-1α in HCC

**Fig. 2:**
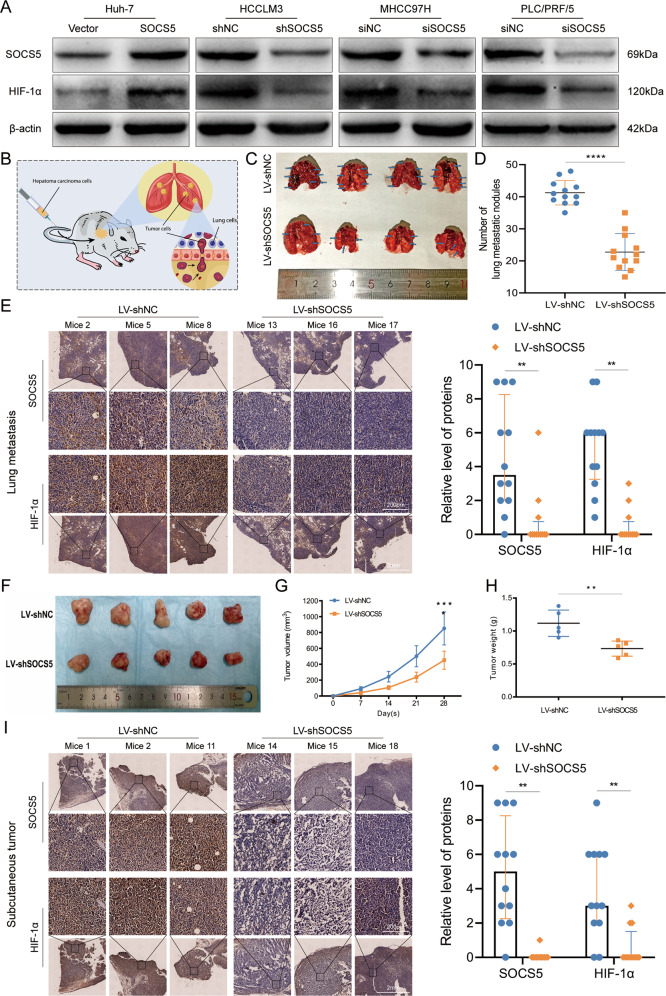
SOCS5 positively regulates the protein expression of HIF-1α in HCC. **A** Western blot analysis of HIF-1α protein expression in HCC cells after upregulation and downregulation of SOCS5. **B** Schematic of lung metastases model. **C** Representative images of lung tissues from nude mice. **D** The number of metastatic nodules in the lungs. **E** Immunohistochemical staining of SOCS5 and HIF-1α protein expression in the lungs. **F** Photographs of tumors after inoculation of stably transfected HCCLM3 cells into nude mice. **G** The tumor volume of shSOCS5-treated HCCLM3 cells in nude mice. **H** Mean tumor volume measured by caliper on the indicated weeks. **I** Immunohistochemical staining of SOCS5 and HIF-1α protein expression in Subcutaneous tumors. **P < 0.01, ***P < 0.001, ****P < 0.0001.

We first determined the mRNA and protein expression levels of SOCS5 in diverse human HCC cell lines (Supplementary Fig. S2A, B). The expression levels of SOCS5 were relatively low in Huh7 and Hep3B cells but higher in PLC/PRF/5, MHCC97H, and HCCLM3 cells. We overexpressed SOCS5 in Huh7 cells and knocked it down in PLC/PRF/5, MHCC97H, and HCCLM3 cells (Supplementary Fig. S2C). Additionally, we verified that SOCS5 protein decreased significantly in HCCLM3 cells with SOCS5 stably knocked down (Supplementary Fig. S2D). To determine the regulatory relationship between SOCS5 and HIF-1α, we detected the protein expression of HIF-1α in the constructed cell model. HIF-1α increased with SOCS5 overexpression in Huh7 cells, but decreased with the knockdown of *SOCS5* in PLC/PRF/5, MHCC97H, and HCCLM3 cells (Fig. 2A, Supplementary Fig. 3A, and Supplementary Table S1). We further examined the in vivo anti-tumor effects of *SOCS5* inhibition in lateral tail vein metastasis and subcutaneous tumorigenesis models. The number of lung metastatic nodules (Fig. 2B–D) and subcutaneous tumor volume and weight (Fig. 2F–H) in nude mice inoculated with HCCLM3 LV-sh*SOCS5* cells were significantly reduced compared with the control group, indicating that the tumor behavior of HCCLM3 cells was significantly reduced upon inhibiting *SOCS5* by shRNA. More importantly, we found that HIF-1α expression was lower in tumors inoculated with stable *SOCS5* knockdown cells than in the control group (Fig. 2E, I). In summary, SOCS5 upregulated the expression of HIF-1α in in vivo and in vitro, and SOCS5 knockdown inhibited HIF-1α expression.

### SOCS5 promotes the invasion and migration of HCC cells by activating EMT and upregulating F-actin expression

**Fig. 3:**
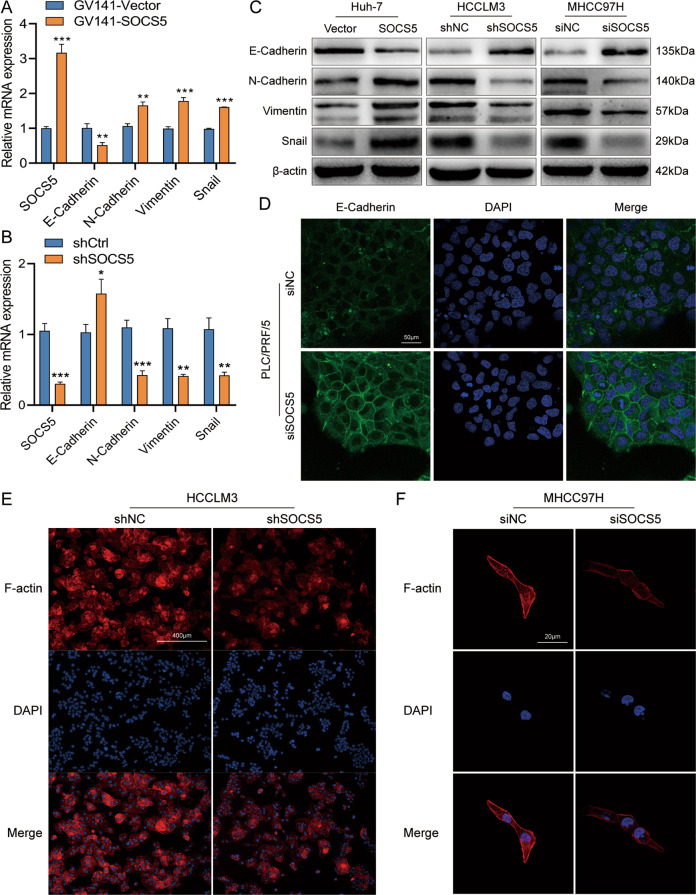
SOCS5 promotes the invasion and migration of HCC cells by activating EMT and upregulating F-actin expression. qPCR analysis of EMT-related mRNA expression in Huh7 (**A**) and HCCLM3 (**B**) cells after upregulation and downregulation of SOCS5. mRNA expression levels were normalized according to the GAPDH expression levels. **C** Western blot analysis of EMT-related protein expression in HCC cells after upregulation and downregulation of SOCS5. **D** Immunofluorescence images of SOCS5 in PLC/PRF/5 cells transfected with siSOCS5 for 48 h. **E**, **F** Immunofluorescence images of F-actin in HCCLM3 (**E**) and MHCC97H (**F**) cells. *P < 0.05, **P < 0.01, and ***P < 0.001.

To further explore the mechanism by which SOCS5 affects HCC cell invasion and migration, we overexpressed SOCS5 in Huh7. PCR and western blot experiments showed that with increased SOCS5, E-cadherin decreased, while N-cadherin, vimentin, and Snail increased, whereas, in HCCLM3 and MHCC97H cells with knockdown of *SOCS5*, we found that E-cadherin increased with a decrease in SOCS5, and N-cadherin, vimentin, and Snail decreased with a decrease in SOCS5 (Fig. 3A–C, Supplementary Fig. 3B, and Supplementary Table S2). After SOCS5 knockdown in PLC/PRF/5 cells, we found that E-cadherin expression increased significantly in the IF experiment (Fig. 3D). Additionally, with the decrease of SOCS5 expression, the rearrangement of F-actin fibers and the expression of F-actin decreased significantly in HCCLM3 and MHCC97H cells (Fig. 3E, F and Supplementary Fig. S3C). These results suggest that SOCS5 promotes the invasion and migration of HCC cells by regulating the expression of EMT-related proteins and F-actin.

### Downregulation of SOCS5 inhibits the invasion and migration of HCC cells by inhibiting HIF-1α expression

**Fig. 4:**
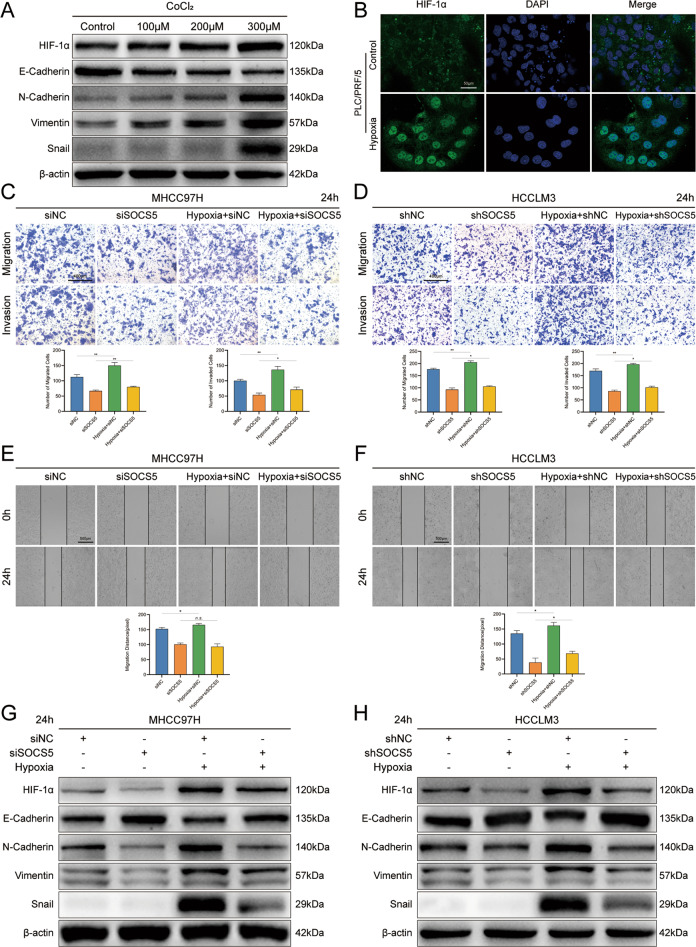
Downregulation of SOCS5 inhibits the invasion and migration of HCC cells by inhibiting the expression of HIF-1α. **A** Western blot analysis of HIF-1α and EMT-related protein expression in HCCLM3 cells under hypoxia induced by Cocl2 (100–300 μM). **B** Immunofluorescence images of HIF-1α in PLC/PRF/5 cells under hypoxia (300 μM). Comparison of the migration and invasion of MHCC97H (**C**) and HCCLM3 (**D**) cells using transwell compartments. Wound-healing assay comparing the motility of MHCC97H (**E**) and HCCLM3 (**F**) cells. Western blotting analysis of HIF-1α and EMT-related protein expression in MHCC97H (**G**) and HCCLM3 (**H**) cells. *P < 0.05, **P < 0.01.

We used CoCl_2_ to induce hypoxia in HCCLM3 cells, which increased HIF-1α protein expression, finding that E-cadherin decreased with increased HIF-1α, while N-cadherin, vimentin, and Snail increased (Fig. 4A, Supplementary Fig. 3D, and Supplementary Table S3). We further confirmed that HIF-1α expression and nuclear migration increased upon induction of hypoxia by CoCl_2_ (300 µM) in PLC/PRF/5 cells (Fig. 4B). To further explore the specific molecular mechanism by which SOCS5 promotes HCC cell invasion and migration, we treated cells with si/sh-SOCS5 and CoCl_2_ in MHCC97H and HCCLM3. We found that SOCS5 knockdown inhibited the invasion and migration of HCC cells under both normoxia and hypoxia (Fig. 4C–F). Compared with the control group (siNC), cells treated with hypoxia induced by CoCl_2_ had stronger invasion and migration abilities. Interestingly, in SOCS5 knockdown cells, the invasion and migration ability after hypoxia was not significantly improved compared to the control group (si*SOCS5)*. Even in the migration experiment with MHCC97H cells, the migration ability of *SOCS5* knockdown cells did not change after hypoxia treatment (*p* = 0.28) (Fig. 4E). We also detected the expression of HIF-1α-and EMT-related proteins, which is consistent with previous conclusions (Fig. 4G, H, Supplementary Fig. S3E, F, and Supplementary Table S3). Therefore, SOCS5 knockdown inhibits HIF-1α-mediated invasion and metastasis, and SOCS5-inhibited HCC cells are less sensitive to changes in the hypoxic environment of ordinary HCC cells.

### SOCS5 downregulation can resist hypoxia-induced mitochondrial damage by inhibiting the expression of HIF-1α

**Fig. 5:**
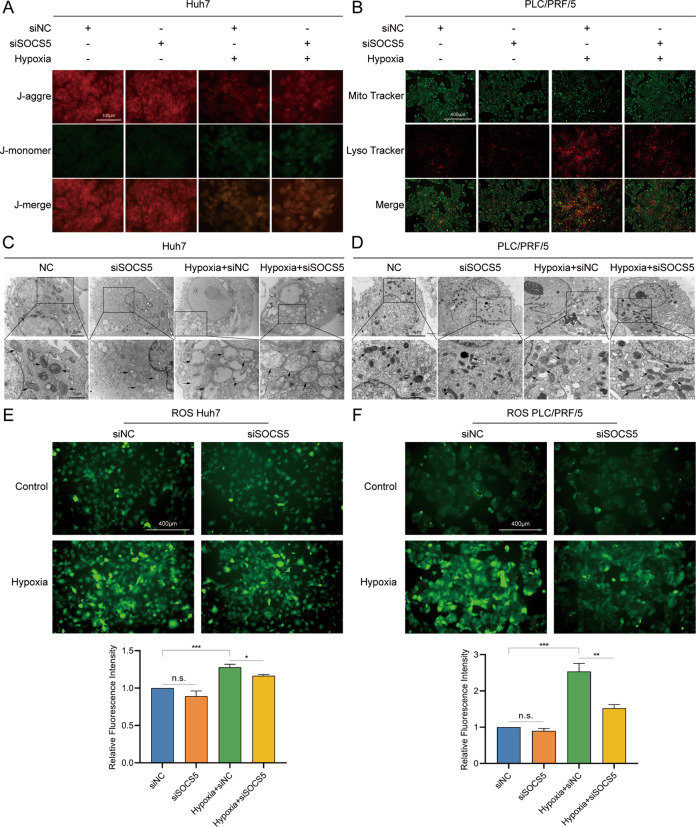
Downregulation of SOCS5 can resist hypoxia-induced mitochondrial damage by inhibiting the expression of HIF-1α. **A** Representative images of JC-1 fluorescence imaging. **B** Immunofluorescence analysis of mitochondria (Mito Tracker Green) and lysosomes (Lyso Tracker Red). Representative electron micrographs of the mitochondrion in Huh7 (**C**) and PLC/PRF/5 (**D**) cells. DCFDA staining showing the ROS levels of Huh7 (**E**) and PLC/PRF/5 (**F**) cells. *P < 0.05, **P < 0.01, and ***P < 0.001.

Studies have shown that hypoxia can lead to mitochondrial damage, release ROS, and promote tumor invasion and metastasis [23, 24]. To explore the effect of SOCS5 on hypoxia-induced mitochondrial damage, we treated the cells with siSOCS5 and CoCl_2_. JC-1 fluorescence imaging showed that hypoxia reduced the mitochondrial membrane potential in cells, suggesting that the mitochondria were damaged. However, the knockdown of SOCS5 could resist the decrease in mitochondrial membrane potential induced by hypoxia, indicating that mitochondria in cells are protected to a certain extent (Fig. 5A). Figure 5B shows that compared with the control group (siNC), hypoxia-induced cells had fewer mitochondria (Mito Tracker Green), more lysosomes (Mito Tracker Red), and more colocalization of mitochondria and lysosomes (yellow puncta). This suggests that tumor cells have more lysosomes to consume mitochondria damaged by hypoxia. Interestingly, SOCS5 knockdown can increase mitochondria, reduce lysosomes, and resist hypoxia-induced mitochondrial damage. We also observed ultrastructural changes in the mitochondria by transmission electron microscopy (Fig. 5C, D). The results showed that hypoxia-induced mitochondrial cristae disappearance, swelling, and vacuolation; further, SOCS5 knockdown attenuated hypoxia-induced mitochondrial damage. Additionally, we measured the level of intracellular ROS using DCFH-DA. The results showed that hypoxia-induced a large release of ROS in cells. Interestingly, SOCS5 knockdown reduced the release of ROS induced by hypoxia (Fig. 5E, F). These results suggest that SOCS5 knockdown resisted hypoxia-induced mitochondrial damage by downregulating HIF-1α expression.

### SOCS5 upregulated HIF-1α expression in HCC by activating the PI3K/Akt/mTOR pathway

**Fig. 6:**
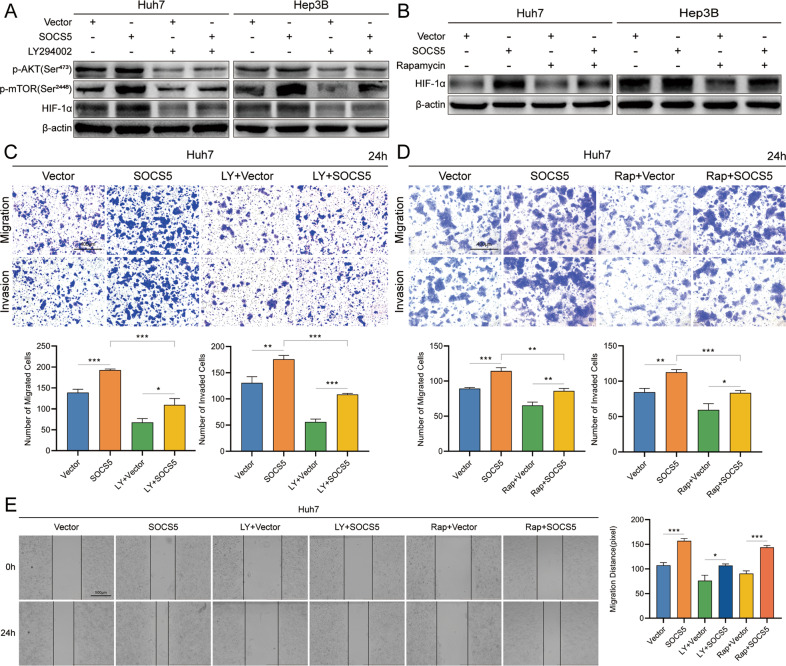
SOCS5 upregulated HIF-1α expression in HCC by activating the PI3K/mTOR/Akt pathway. **A** Western blot analysis of p-Akt (Ser473), p-mTOR (Ser2448), and HIF-1α protein expression in Huh7 and Hep3B SOCS5-OE-cells treated with 20 μg/mL LY294002 for 48 h. **B** Western blot analysis of HIF-1α protein expression in Huh7 and Hep3B SOCS5-OE-cells treated with 10 μg/mL Rapamycin for 48 h. Comparison of the migration and invasion of Huh7 SOCS5-OE-cells treated with LY294002 (**C**) or Rapamycin (**D**) using transwell compartments. **E** Wound-healing assay comparing the motility of Huh7 SOCS5-OE-cells cells. *P < 0.05, **P < 0.01, and ***P < 0.001.

To explore the molecular mechanism by which SOCS5 regulates HIF-1α, we treated HCC cells (Huh7 and Hep3B) with a GV141-SOCS5 and PI3K inhibitor (LY294002) or mTOR inhibitor (rapamycin) and then detected changes in HIF-1α protein expression. HIF-1α expression in cells treated with LY294002 or rapamycin alone decreased significantly compared with the control group (Vector), and increased after the combined overexpression of SOCS5 (Fig. 6A, B, Supplementary Fig. 3G, H, and Supplementary Table S4). More importantly, we treated cells with GV141-SOCS5 and LY294002 or rapamycin in Huh7, finding that LY294002 or rapamycin inhibited invasion and migration, which was reversible by overexpression of SOCS5 (Fig. 4C–E). These results suggest that SOCS5 promotes the invasion and migration of HCC cells by activating the PI3K/Akt/mTOR pathway.

### Downregulation of SOCS5 inhibits PM-induced HCC metastasis

**Fig. 7:**
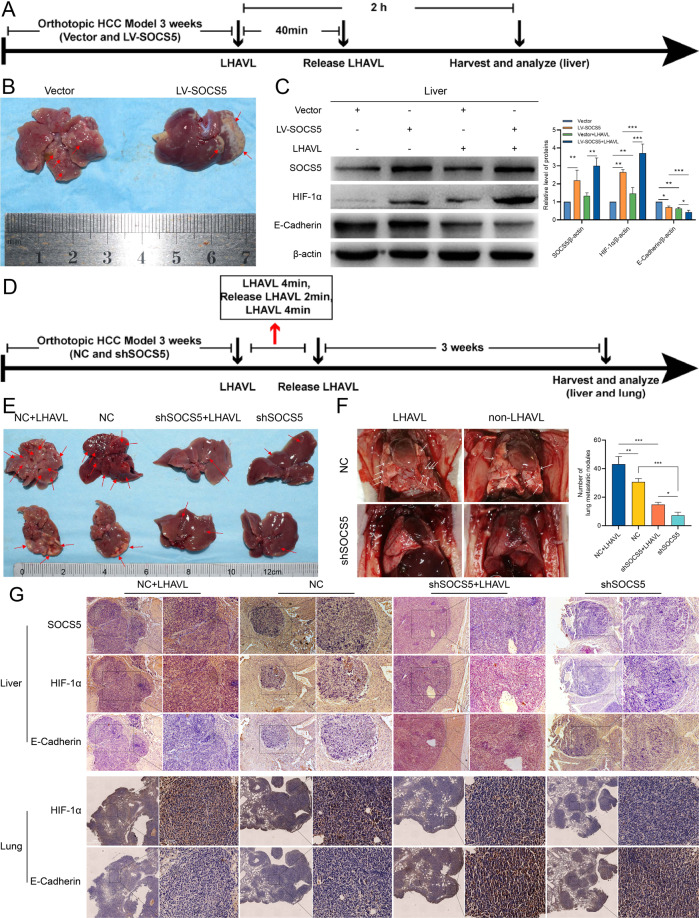
Downregulation of SOCS5 inhibits PM-induced HCC metastasis. **A** Schematic diagram of the protocol used to measure the changes in the expression of hypoxic and EMT markers following transient left hepatic artery and vein ligation (LHAVL) of the left lobe of the liver. **B** Representative images of orthotopic HCC model (LV-SOCS5). **C** Western blot analysis of SOCS5, HIF-1α, and E-Cadherin protein expression in orthotopic HCC treated with LHAVL for 2 h. **D** Schematic diagram of the protocol used to measure the changes in the expression of hypoxic and EMT markers following intermittent LHAVL of the left lobe of the liver. **E** Representative images of orthotopic HCC model (shSOCS5) treated with intermittent LHAVL. **F** Representative images of the corresponding lung metastasis model of orthotopic HCC after intermittent LHAVL. The number of metastatic nodules in the lungs. **G** Immunohistochemical staining of SOCS5, HIF-1α, and EMT protein expression in the liver and lungs. *P < 0.05, **P < 0.01, and ***P < 0.001.

We implanted HCCLM3 overexpressing SOCS5 into the left lobe of the liver of nude mice (Fig. 7A), and constructed an orthotopic HCC model overexpressing SOCS5 (Fig. 7B). Then LHAVL was performed, HCC tumors in the left lobe of the liver were collected 2 h later, and the blocking was released 40 min after LHAVL. We found that LHAVL could increase the expression of HIF-1α protein and decrease the expression of E-Cadherin in HCC (Fig. 7C). More importantly, after LHAVL was performed on HCC tumors overexpressing SOCS5, HIF-1α expression significantly increased and E-Cadherin expression significantly decreased. Then, we implanted SOCS5 knockdown HCCLM3 in the left lobe of the liver of nude mice, and constructed an orthotopic HCC model with SOCS5 knockdown. Then LHAVL was performed intermittently, blocking for 4 min, opening for 2 min, and then releasing the blocking after 4 min of blocking again (Fig. 7D). After 3 weeks, the liver and lungs of nude mice were collected. Liver (Fig. 7E) and lung (Fig. 7F) gross specimens revealed that the knockdown of SOCS5 significantly inhibited the intrahepatic metastasis and lung metastasis of HCC. More importantly, LHAVL promoted HCC intrahepatic metastasis and lung metastasis in the NC groups, whereas the metastatic effect of LHAVL on HCC with SOCS5 knockdown was attenuated. We collected liver left lobe HCC for IHC experiments (Fig. 7G) and found that SOCS5 was significantly higher in the NC groups than in the knockdown SOCS5 groups, and more importantly, LHAVL in the NC groups obviously promoted the expression of HIF-1α and suppressed the expression of E-cadherin, interestingly, LHAVL in the knockdown SOCS5 groups failed to obviously alter the expression of HIF-1α and E-cadherin. Similarly, our IHC experiments on nude mice lungs revealed that LHAVL obviously promoted the expression of HIF-1α and suppressed the expression of E-cadherin in the NC groups, but LHAVL failed to obviously alter the expression of HIF-1α and E-cadherin in the knockdown SOCS5 groups (Fig. 7G).

## Discussion

Previous research of our research group shows that SOCS5 plays an important role in promoting the progress of HCC, especially by enhancing HCC metastasis [14]. This is consistent with the research of some scholars [25, 26]. Some scholars also proposed the tumor inhibition effect of SOCS5 in HCC [27]. These authors had not examined SOCS5 expression in HCC samples, studied cell growth as the only phenotype, and showed increased growth of their xenografts from HCC cells transfected seemingly transient SOSC5 siRNA. It is also possible that SOCS5 regulates HCC growth and progression in a complex manner.

**Fig. 8:**
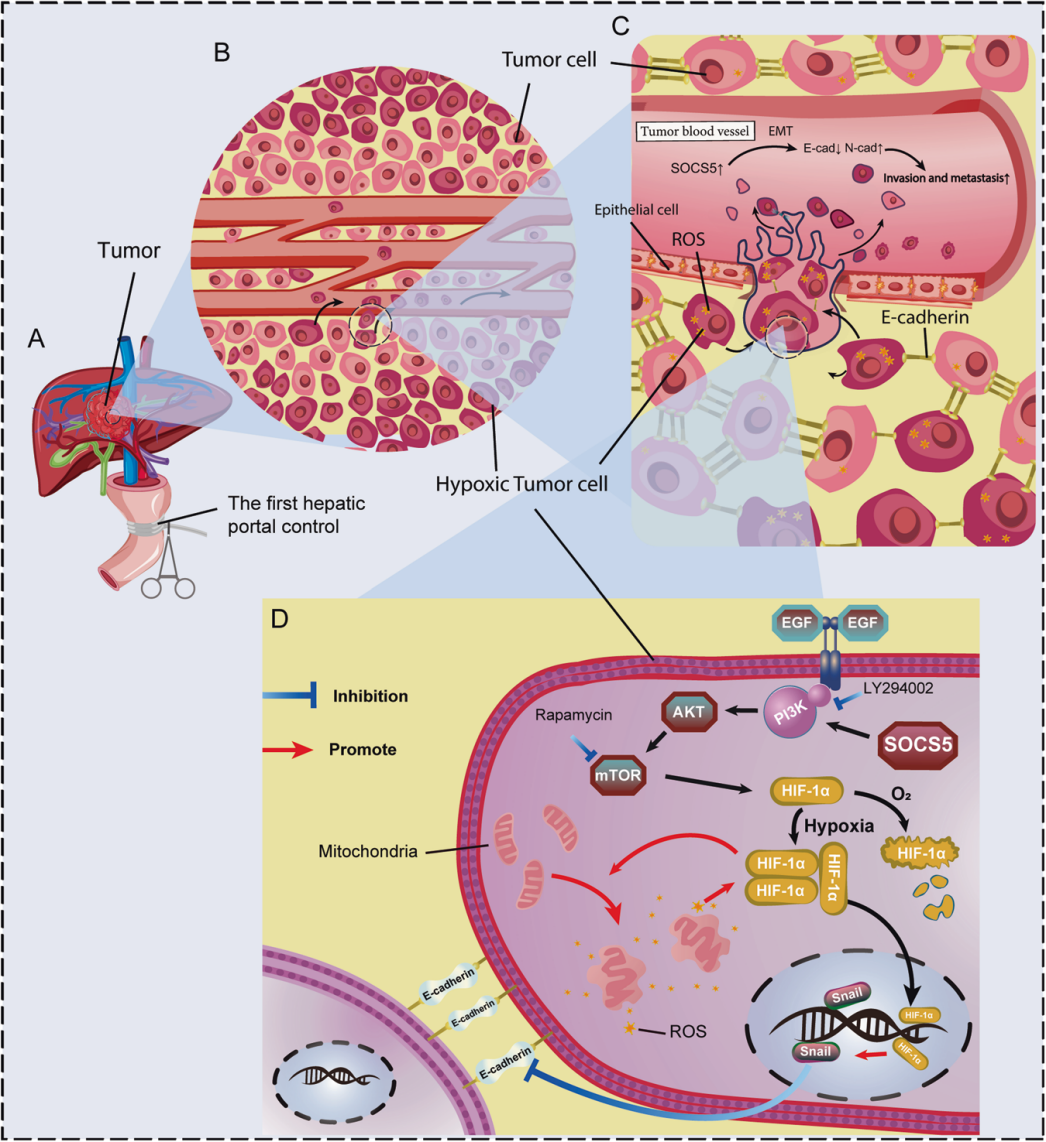
Schematic diagram depicting the mechanism by which SOCS5 promoted the invasion and migration of HCC cells by activating PI3K/Akt/mTOR-HIF-1α signal axis. **A** The first hepatic portal control. **B** Tumor cells far away from blood vessels are more hypoxic and invasive. **C** The connections between hypoxic tumor cells were loose and there were more ROS. **D** SOCS5 promoted the invasion and migration of HCC cells by activating PI3K/Akt/mTOR-HIF-1α signal axis.

In this study, we found that HCC patients with low SOCS5 expression were more tolerant to hypoxia resulting from the PM. We investigated the hypoxic tumor microenvironment, finding that SOCS5 promoted the invasion and migration of HCC cells by activating the PI3K/Akt/mTOR-HIF-1α signaling axis. Meanwhile, downregulating SOCS5 not only inhibited HCC invasion and metastasis but also resisted hypoxia-induced mitochondrial damage by downregulating HIF-1α expression (Fig. 8). More importantly, we found that PM can promote HCC tumor metastasis in metastatic human HCC orthotopic nude mouse model, and HCC tumors with knockdown of SOCS5 were more resistant to hypoxia caused by PM.

PM is the safest and simplest method to reduce bleeding during hepatectomy. It is the most commonly used vascular inflow control technology in liver surgery [28]. However, PM blocks the blood supply to the tumor, leading to changes in the hypoxic microenvironment and promoting tumor micrometastasis, which in turn affects patient prognosis [29–31]. Our findings support this conclusion. Interestingly, we found that the PM duration in HCC patients with high SOCS5 expression had a highly significant effect on patient outcomes, but the PM duration in HCC patients with low SOCS5 expression did not affect patient outcomes. Further, the hypoxia score in the high SOCS5 expression group was higher than that in the low-expression group. This suggests that HCC patients with low SOCS5 expression were more tolerant to changes in the tumor-hypoxic microenvironment induced by PM, and that SOCS5 might play a crucial role in the tumor-hypoxic microenvironment. Critically, we have shown that SOCS5 positively regulates the expression of HIF-1α protein in clinical tissue samples, HCC cell experiments, and animal experiments. In summary, our results show that SOCS5 upregulates the expression of HIF-1α in vivo and in vitro, and SOCS5 knockdown inhibits its expression. This study fills the gap of knowledge on the relationship between SOCS5 and HIF-1α.

Tumor cells can regulate the expression of intercellular adhesion molecule E-cadherin and the rearrangement of F-actin to promote tumor invasion and metastasis [32, 33]. SOCS5 promoted the rise of interstitial phenotypic markers to competitively inhibit the expression of the epithelial phenotypic marker E-cadherin, thereby promoting invasion and migration. Concurrently, we found that SOCS5 knockdown reduced the rearrangement of F-actin fibers and the expression of F-actin. Hypoxia activates related pathways by inducing HIF-1α protein expression and nuclear metastasis, promoting tumor invasion and metastasis [8, 34], consistent with our data. More importantly, we found that hypoxia-induced invasion and migration could be reversed by inhibiting SOCS5, consistent with the expression of EMT-related marker proteins. Interestingly, in SOCS5 knockdown cells, the invasion and migration abilities after hypoxia were not significantly improved compared with the control group. This indicated that SOCS5-inhibited HCC cells were less sensitive to changes in the hypoxic environment than ordinary HCC cells. This may explain why patients with low SOCS5 expression were more tolerant to PM-induced hypoxia. Therefore, our results show that SOCS5 knockdown inhibits HIF-1α mediated invasion and metastasis, providing a deeper understanding of the oncogenic molecular mechanism by which SOCS5 promotes HCC progression, and providing clues for the anti-invasive and anti-metastatic treatment of HCC.

Hypoxia can cause mitochondrial damage and increase ROS levels, thereby promoting tumor progression [23, 24]. However, cells exposed to a hypoxic environment activate HIF-1α dependent transcriptional targets, in turn inhibiting ROS production [35, 36]. Intermittent hypoxia induces ROS burst release, activates some transcriptional factors (e.g., AP1, MMP, Smad, and Snail), and promotes cancer progression [37–39]. However, high ROS levels can cause oxidative stress, affect DNA oxidation, and induce pro-apoptotic pathways [40]. Therefore, tumor cells would consume more ROS than they can tolerate, so that the level of intracellular ROS remains low while being conducive to tumor progress [40]. Our results showed that hypoxia-induced mitochondrial damage released large ROS quantities in a short time, consistent with the results of previous studies. Additionally, SOCS5 inhibition significantly inhibited Snail expression in hypoxic environments, consistent with the conclusion that ROS upregulates Snail and activates EMT [41]. SOCS5 knockdown reversed hypoxia-induced mitochondrial damage, reduced the hypoxia-induced release of mitochondrial ROS, inhibited snail expression, and inhibited invasion and metastasis. To date, there is no literature on the relationship between SOCS5 and mitochondria, and our study bridges this gap.

SOCS5 activates the PI3K/Akt/mTOR pathway [14], a classical pathway that regulates HIF-1α protein expression [12]. To explore the specific molecular mechanism of SOCS5 regulation of HIF-1α, we performed a rescue experiment using the SOCS5-PI3K/Akt/mTOR-HIF-1α axis with PI3K and mTOR inhibitors. SOCS5 upregulates the expression of HIF-1α by activating the PI3K/Akt/mTOR signaling pathway, thereby promoting the invasion and migration of HCC cells. These results suggest that the combined targeting of SOCS5 and PI3K/Akt/mTOR pathway provides an option to weaken tumor metastasis.

We found that HCC with low expression of SOCS5 were more resistant to PM-induced hypoxia from clinical (Fig. 1) and cellular experiments (Fig. 4). To further explore the role of SOCS5 in PM-induced HCC metastasis, we constructed orthotopic HCC models with overexpression of SOCS5 and knockdown of SOCS5. There are studies that show that a significant decrease in pO2 was observed after hepatic artery ligation (HAL) plus hepatic vein ligation (HVL), but not HAL alone [19]. This result indicated that the pO2 of HCC in the orthotopic xenograft model was supplied by both the hepatic artery and vein. So, we choose LHAVL to simulate PM in surgery. In an overexpressed SOCS5 orthotopic HCC model, we showed that LHAVL-induced HIF-1α expression promotes HCC tumor metastasis and, more importantly, that HCC tumors overexpressing SOCS5 undergo LHAVL, with a significant rise in metastatic ability. In the knockdown SOCS5 orthotopic HCC model, we found that in the NC groups, LHAVL-induced HIF-1α expression and promoted HCC intrahepatic metastasis and lung metastasis, whereas HCC with knockdown of SOCS5 was more resistant to LHAVL-induced hypoxia and metastasis. This is in keeping with previous literature results that further hypoxia in HCC induced the expression of HIF-1α and promoted metastasis [42–45]. In this study, we obtained consistent conclusions from clinical, cellular, and animal studies that HCC with low SOCS5 expression is tolerant to hypoxia-induced metastasis.

In conclusion, we identified a novel role of SOCS5 in regulating HIF-1α dependent mitochondrial damage, invasion, and metastasis through the PI3K/Akt/mTOR pathway. SOCS5 knockdown counteracted hypoxia-induced mitochondrial damage and inhibited hypoxia-induced invasion and metastasis by inhibiting the PI3K/Akt/mTOR pathway. More importantly, our data suggest that the development of a SOCS5-specific inhibitor, an indirect inhibitor of HIF-1α, may be effective in controlling Pringle maneuver-induced tumor micrometastases during liver cancer resection.

## Supplementary information

original data files

Supplementary Figures

Supplementary Table S1

Supplementary Table S2

Supplementary Table S3

Supplementary Table S4

aj-checklist

## Data Availability

All data generated or analyzed during this study are included either in this article or in the supplemental materials files. Additional raw data may be available from the corresponding author for reasonable reasons.
