# Supplementary material for: SOCS5 knockdown suppresses metastasis of hepatocellular carcinoma by ameliorating HIF-1α-dependent mitochondrial damage
Source: Cell Death Dis. 2022 Nov 1;13(11):918. doi: 10.1038/s41419-022-05361-z (PMC9626553; doi:10.1038/s41419-022-05361-z)

Figure 2_A_HUH7

SOCS5

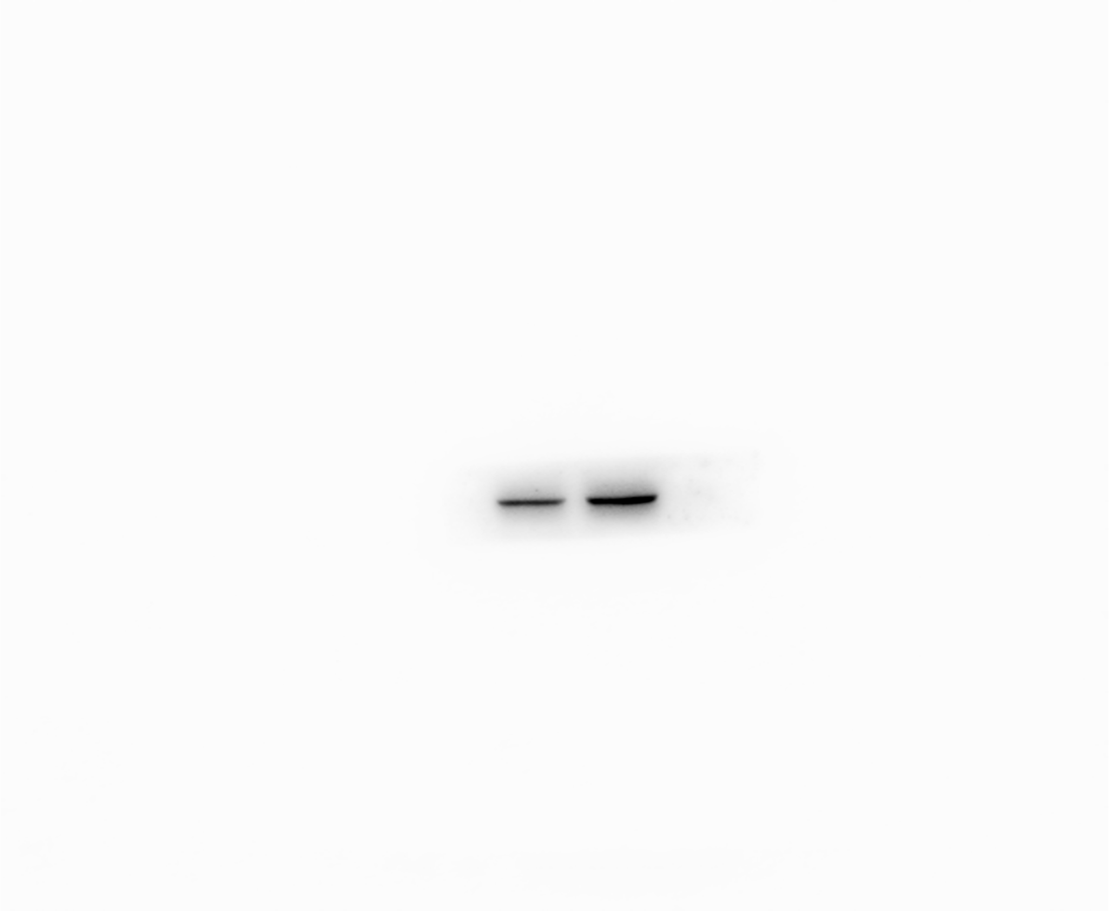

HIF-1α

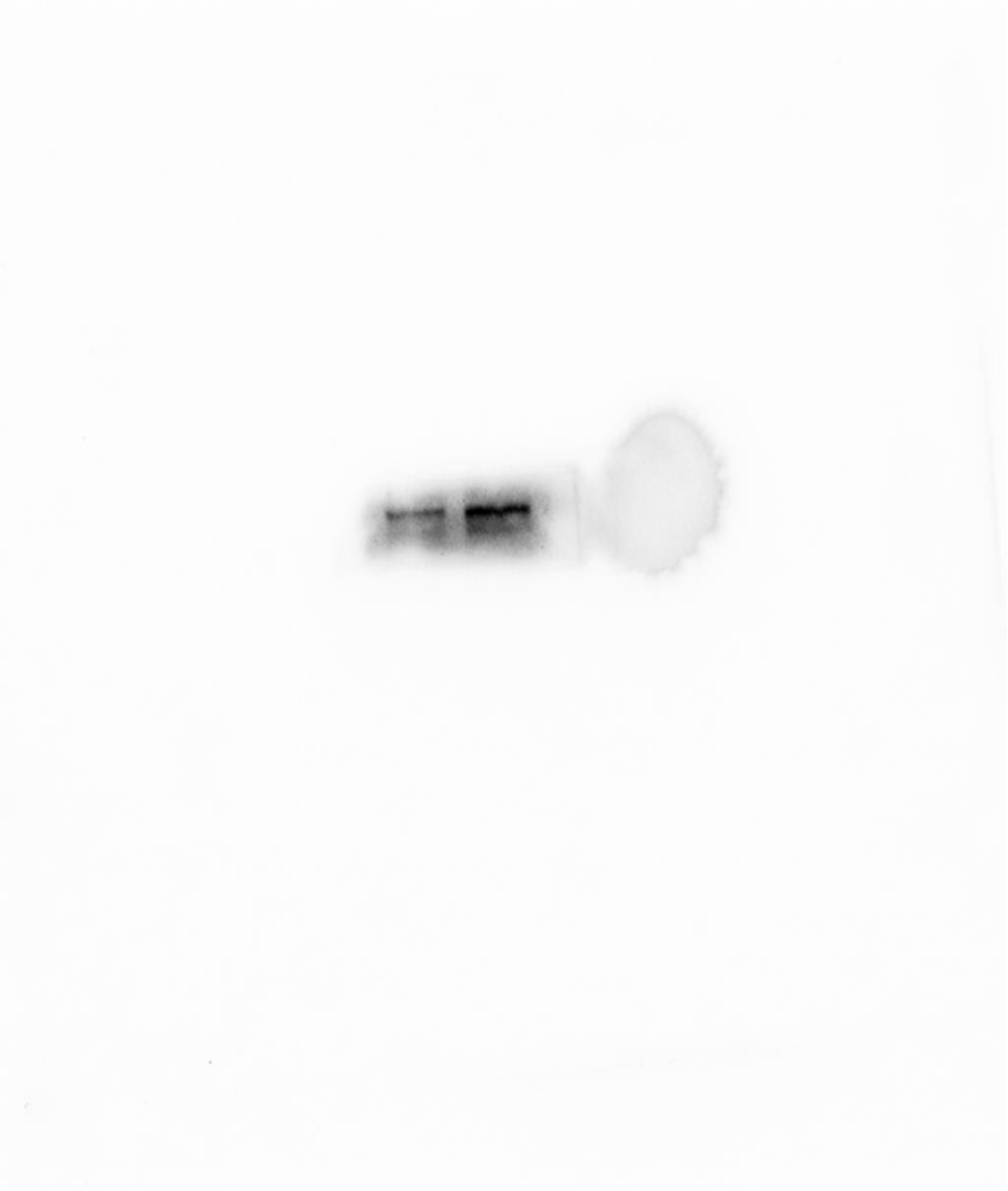

β-ACTIN

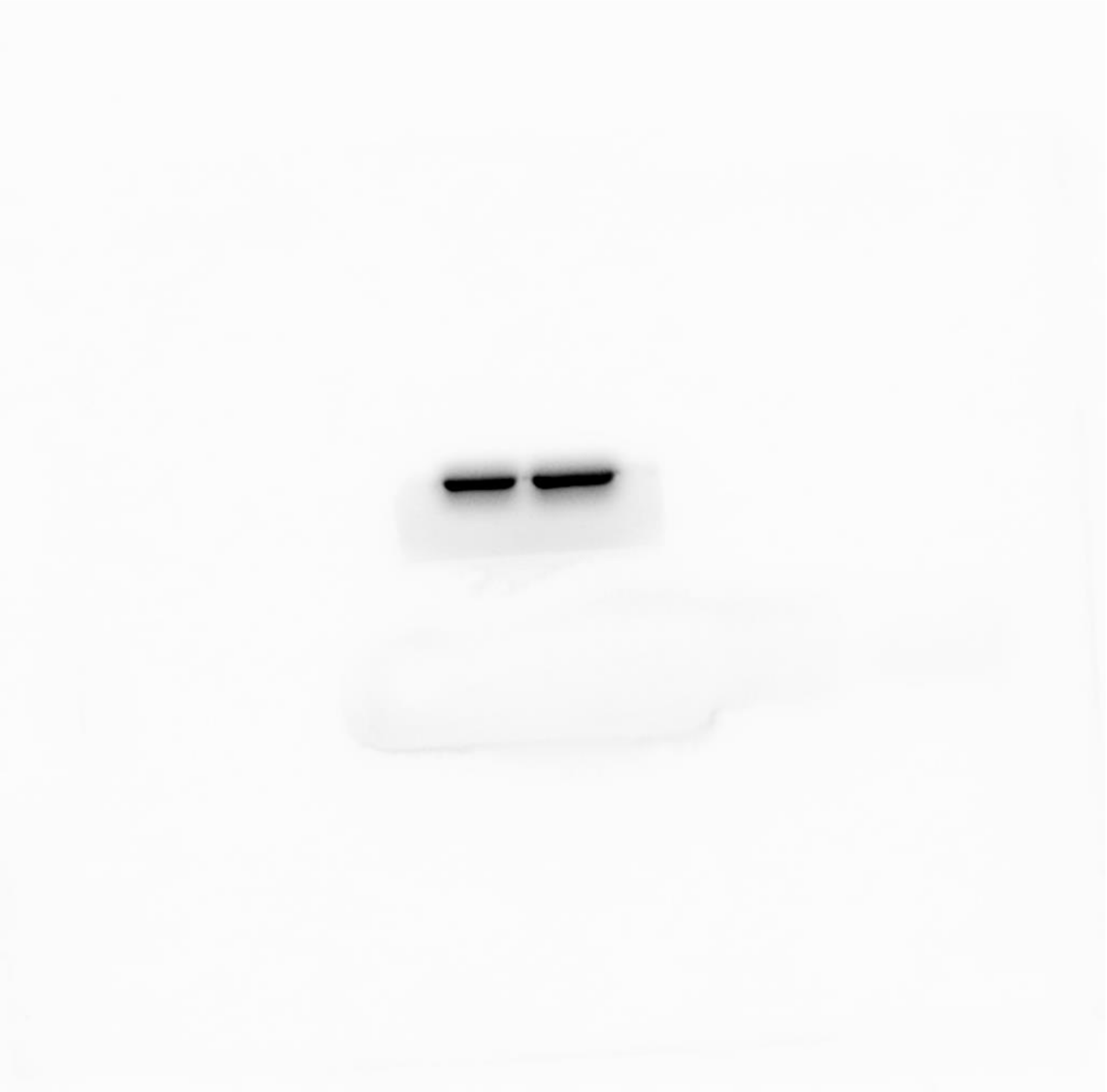

Figure 2_A_HCCLM3

SOCS5

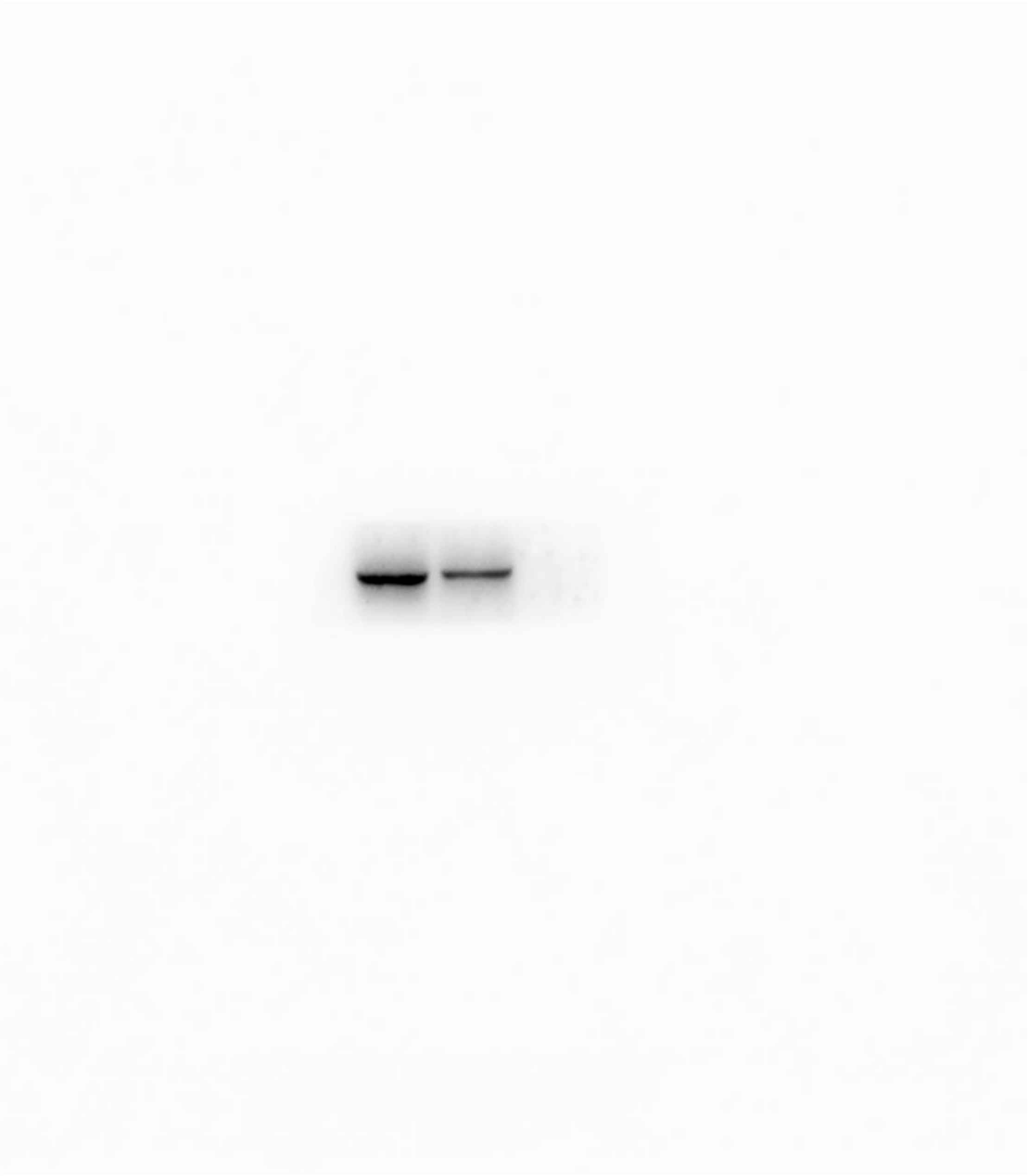

HIF-1α

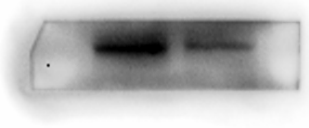

β-ACTIN

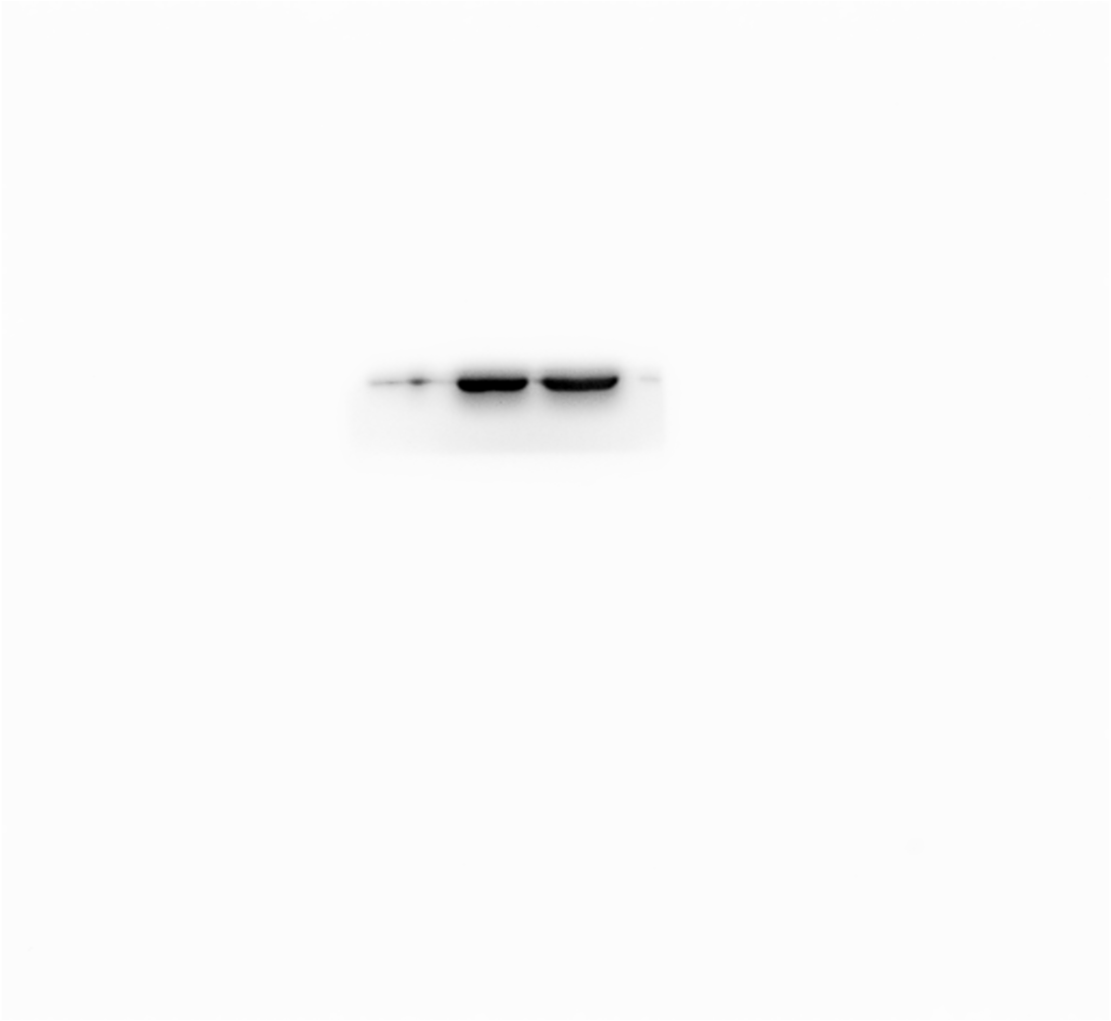

Figure 2_A_MHCC97H

SOCS5

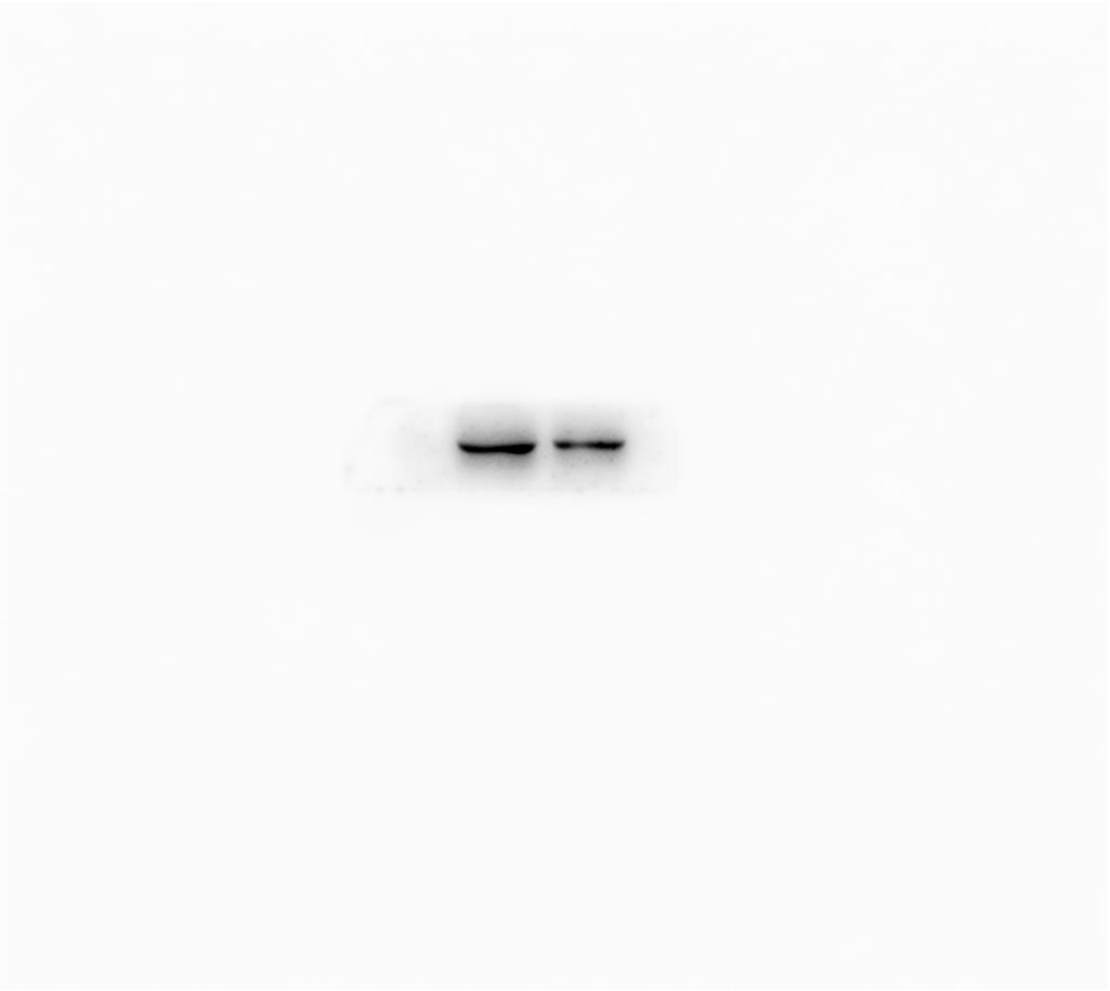

HIF-1α

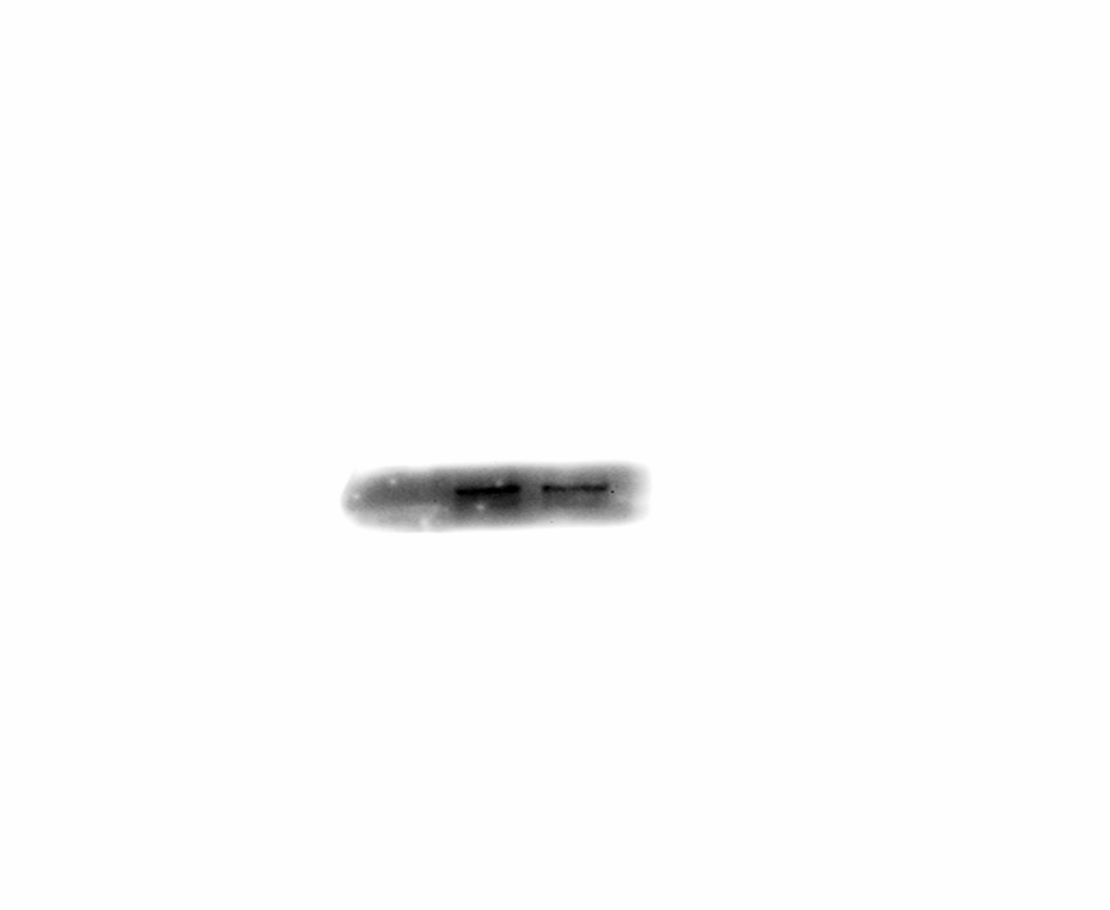

β-ACTIN

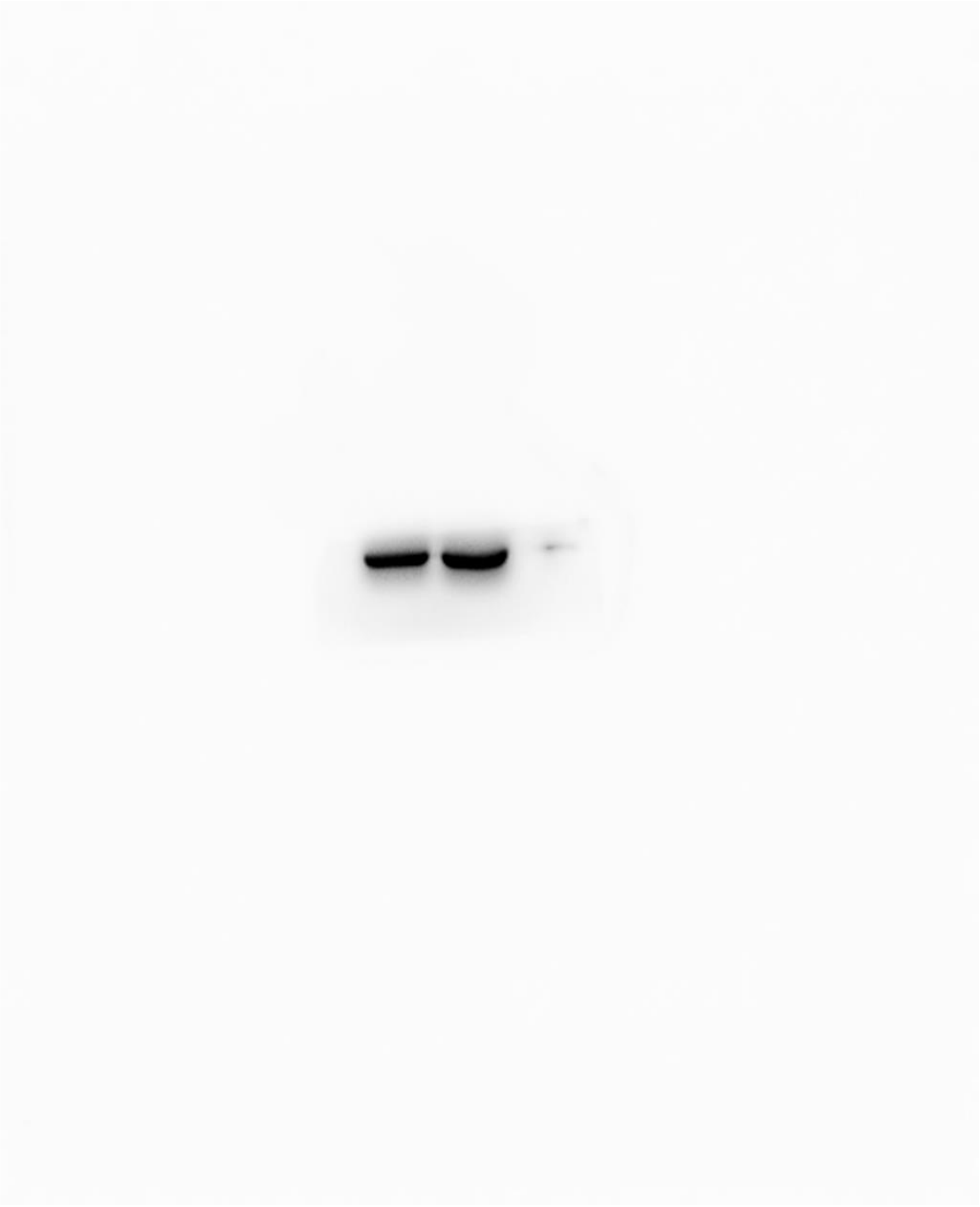

Figure 2_A_PLC/PRF/5

SOCS5

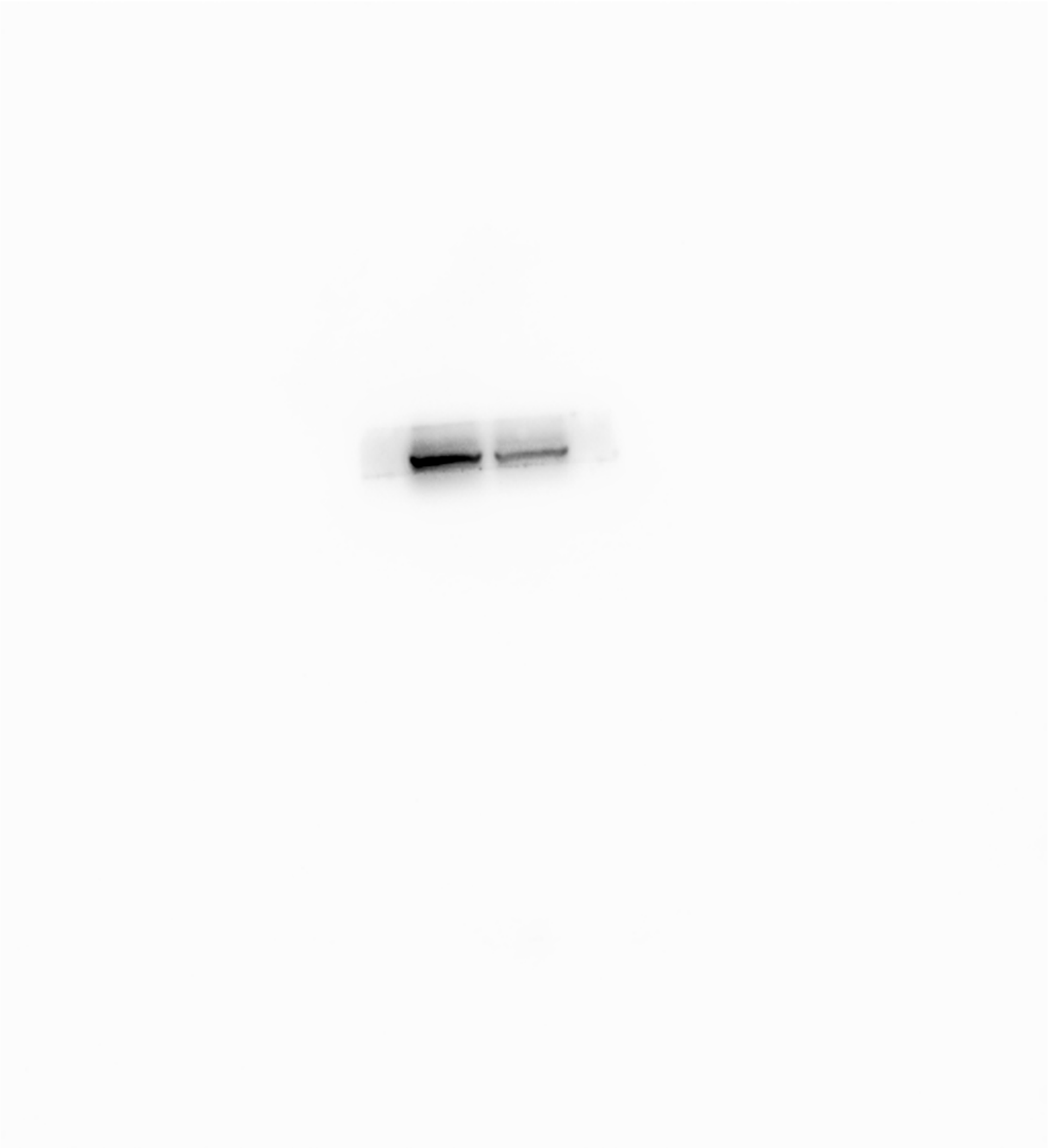

HIF-1α

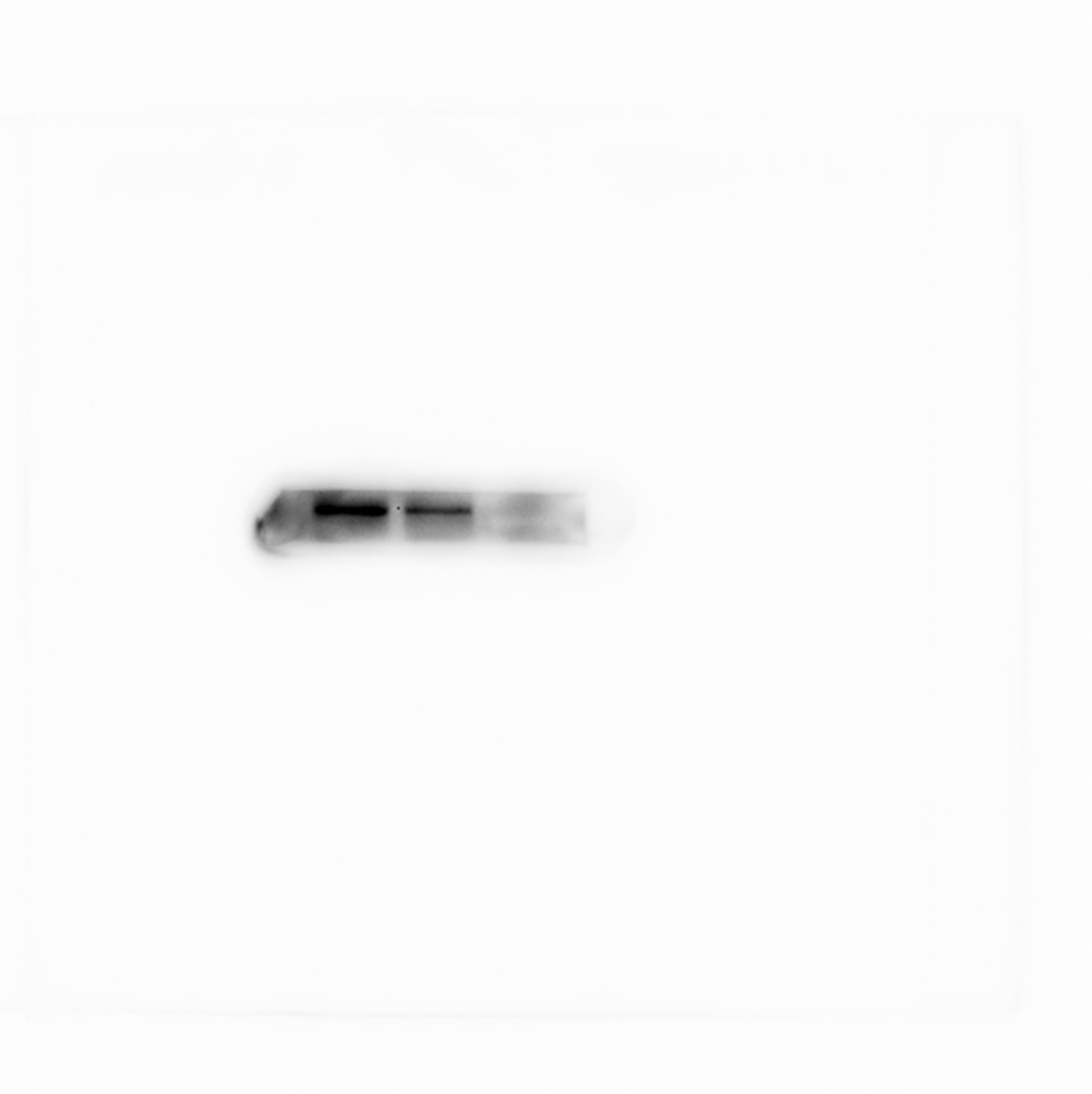

β-ACTIN

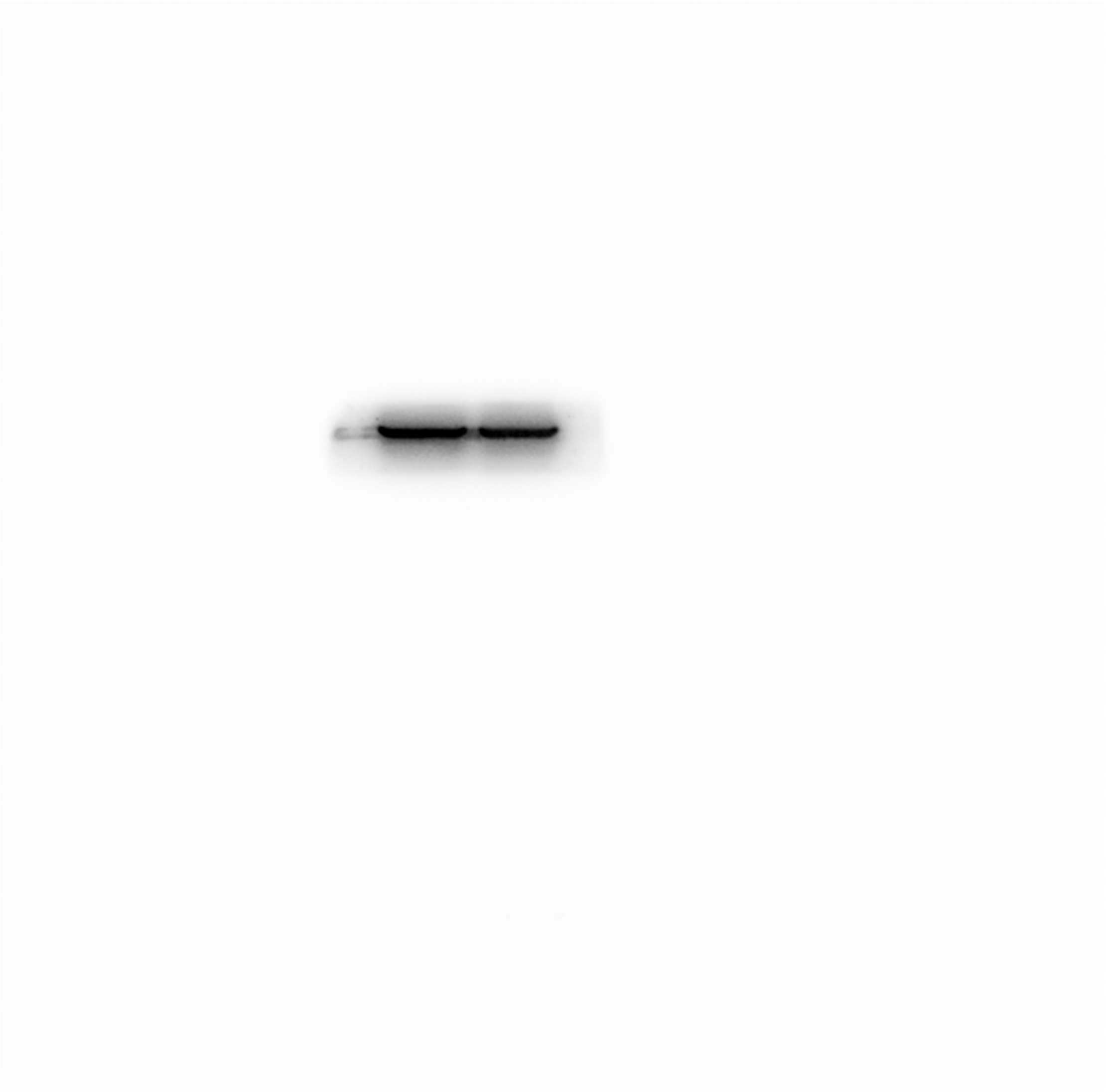

Figure 3_C_HUH7

E-Cadherin

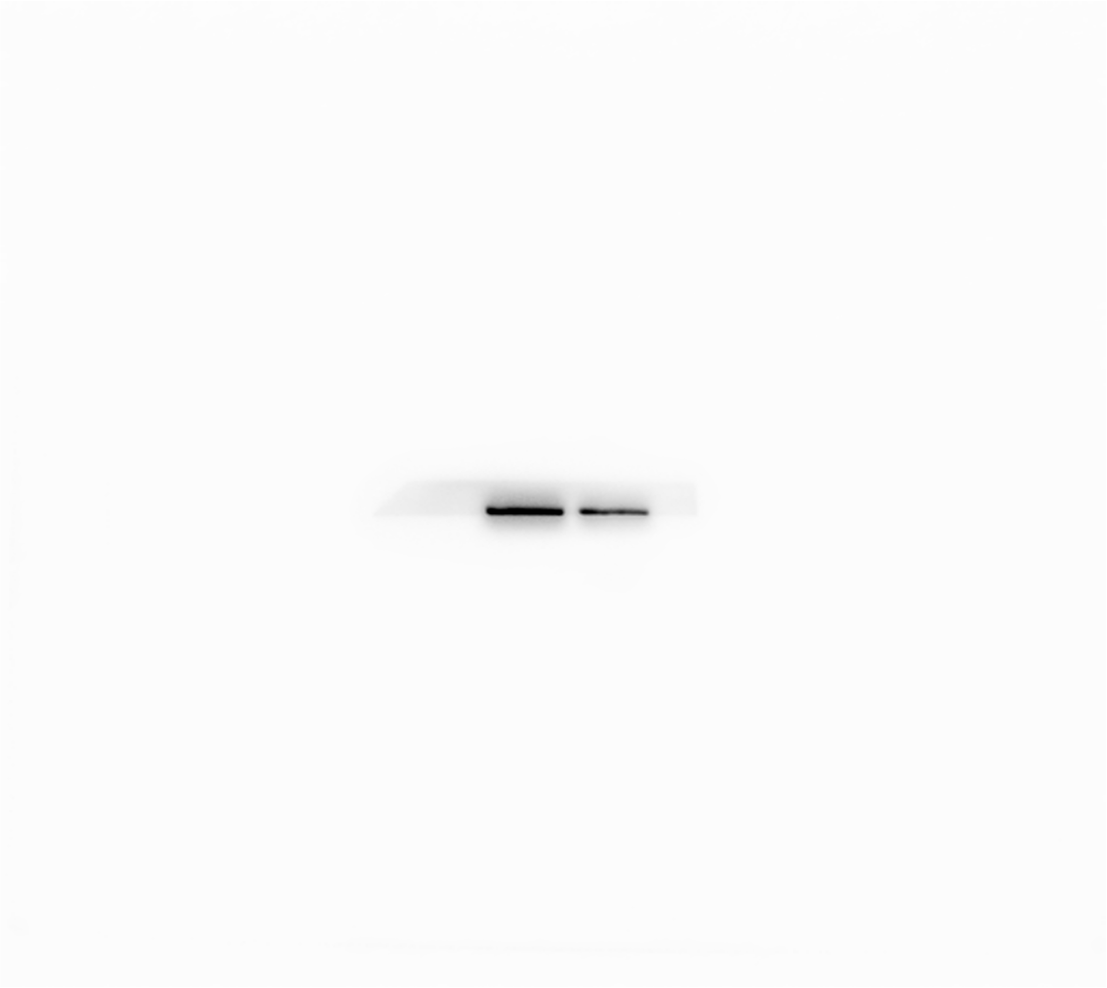

N-Cadherin

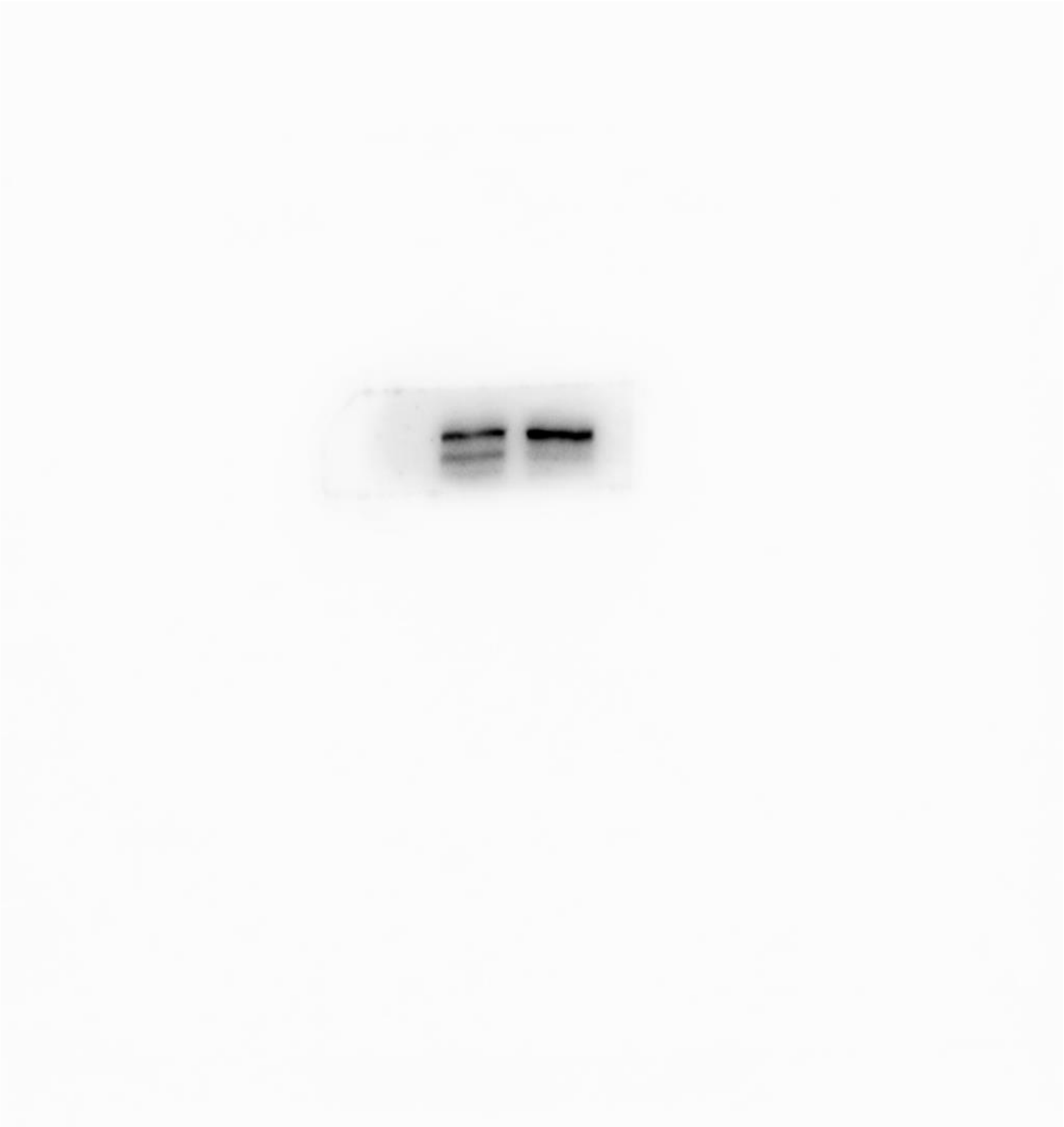

Vimentin

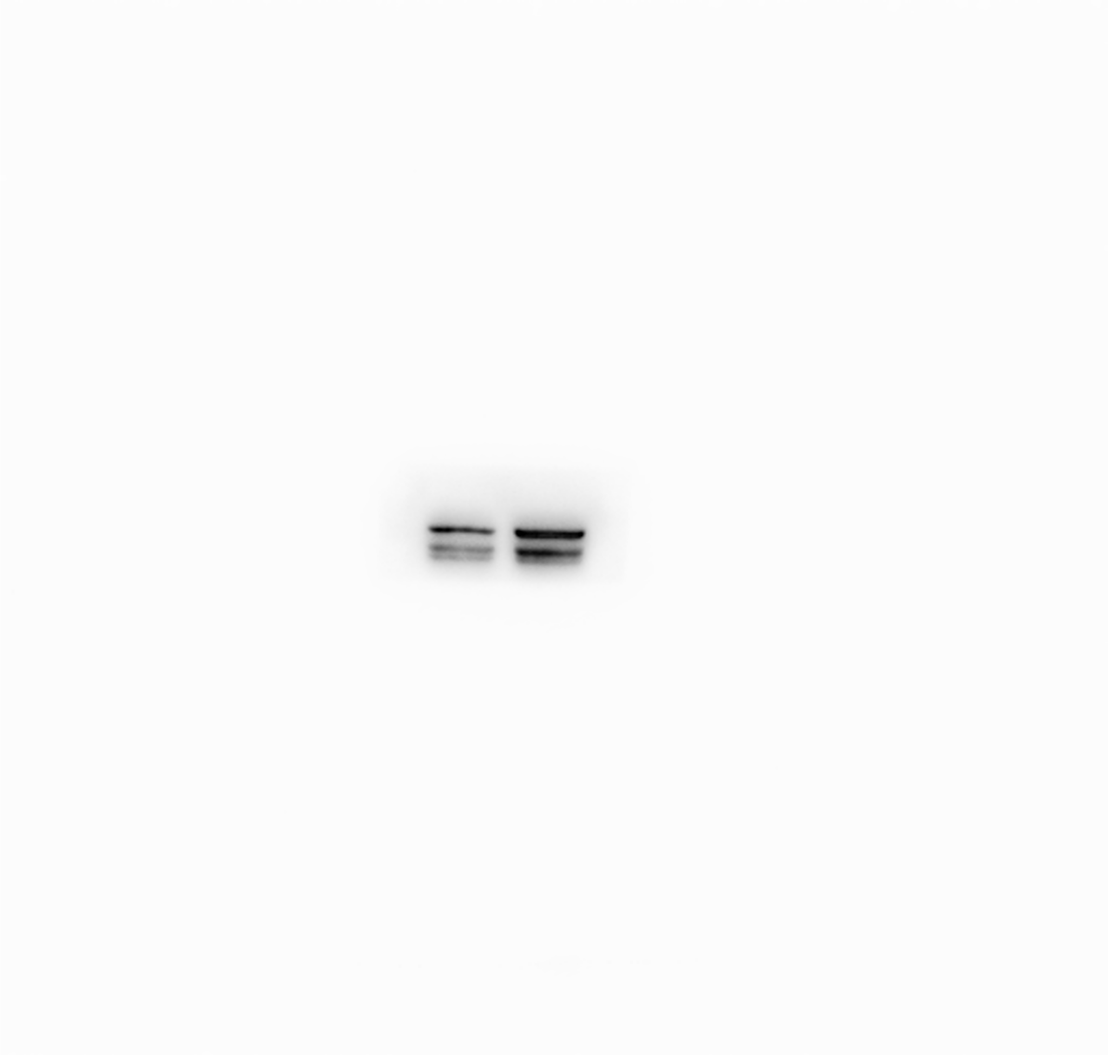

Snail

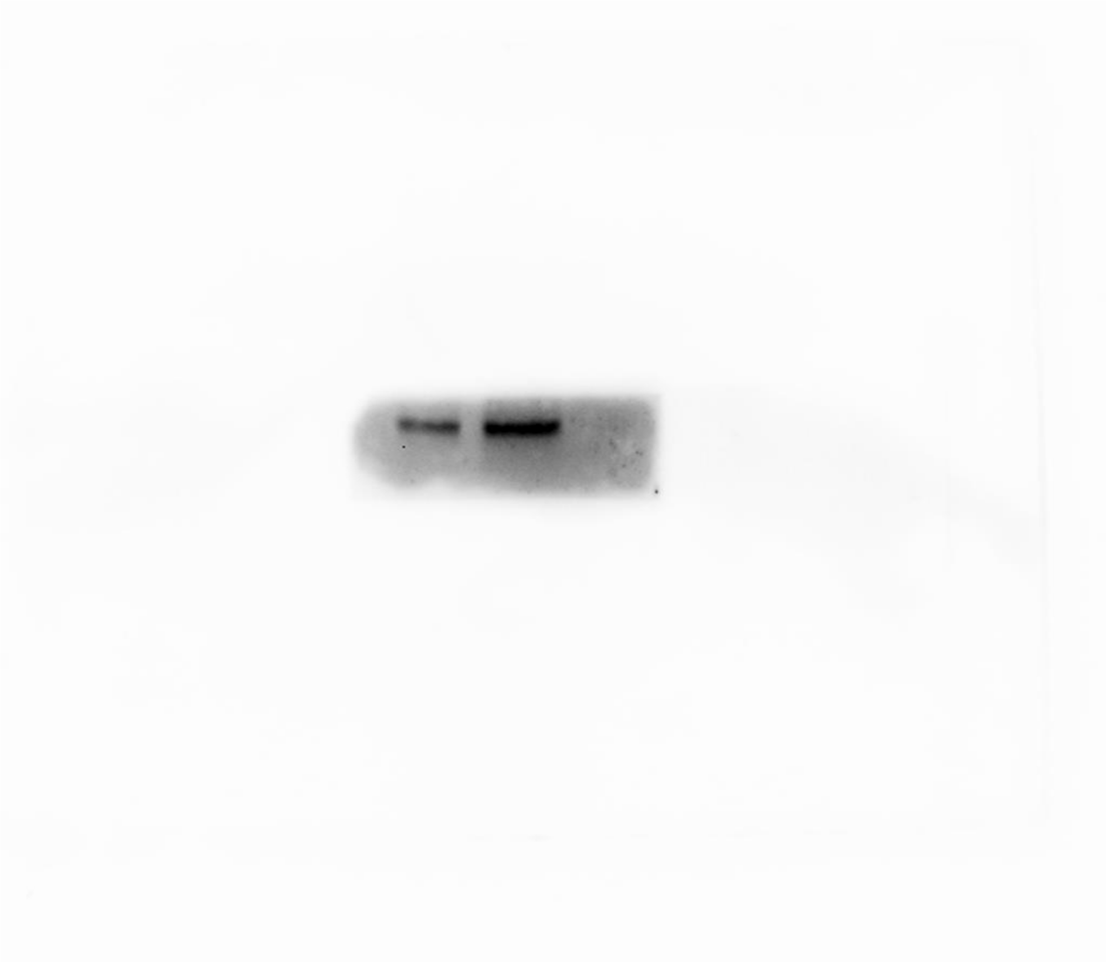

β-ACTIN

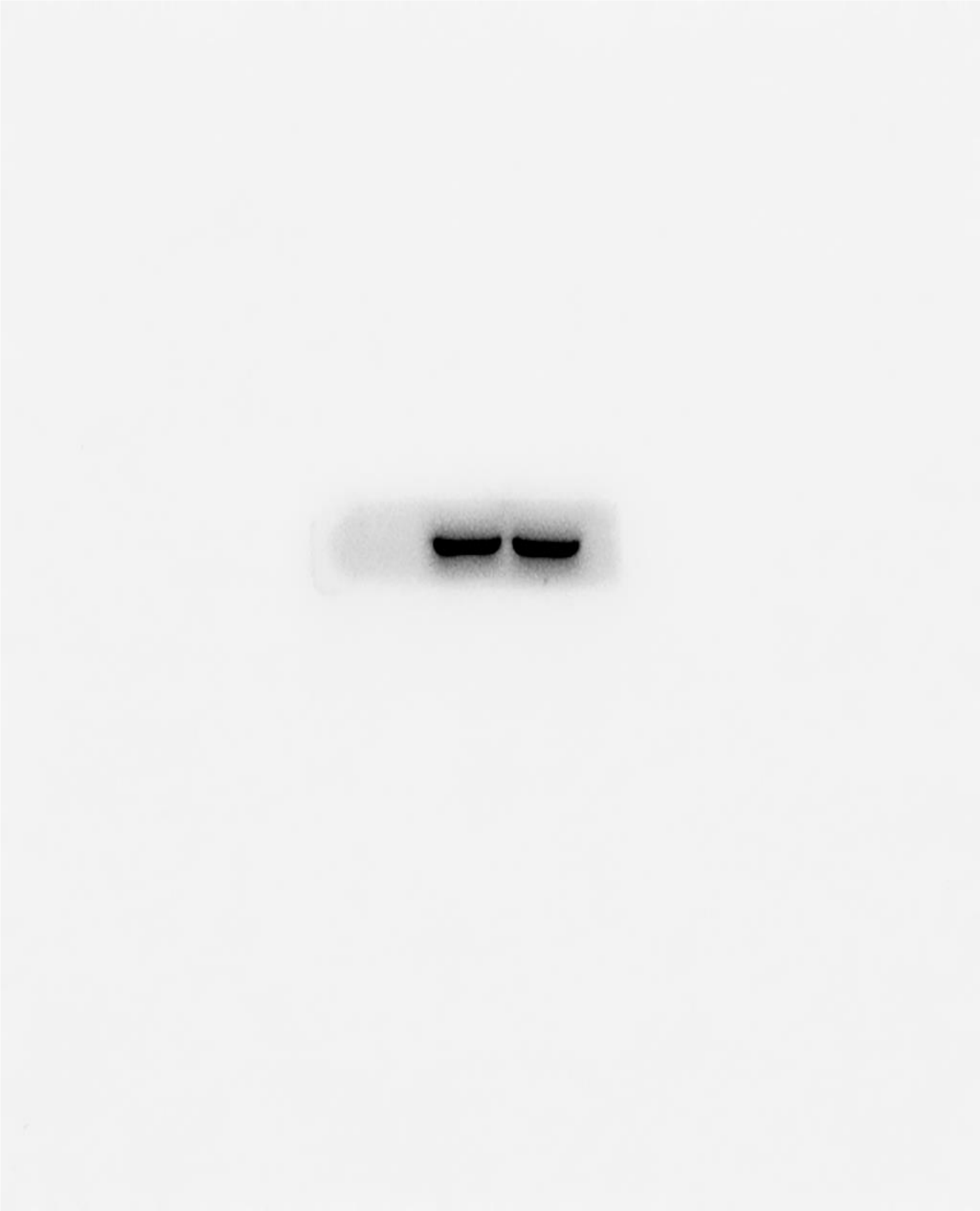

Figure 3_C_HCCLM3

E-Cadherin

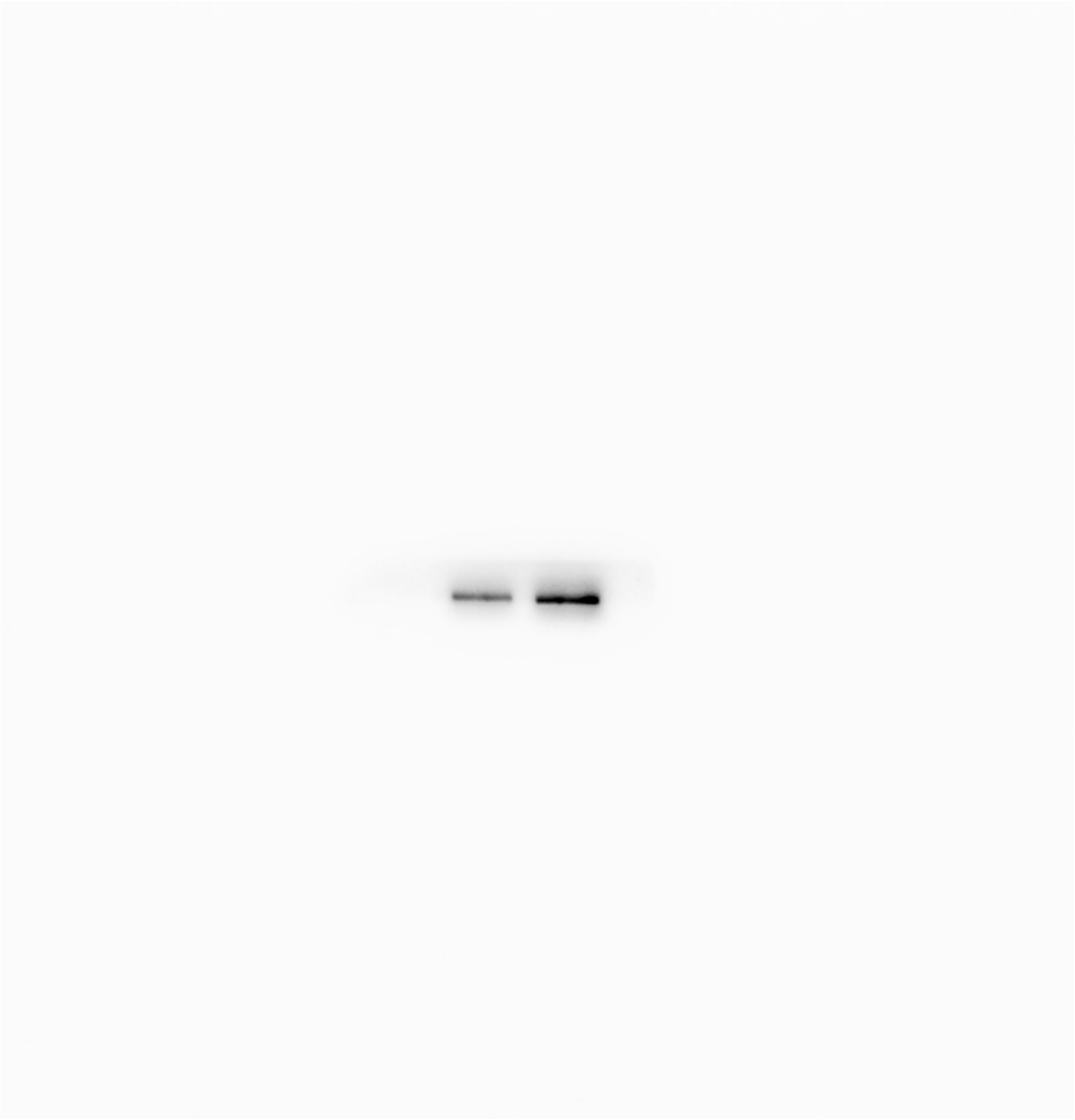

N-Cadherin

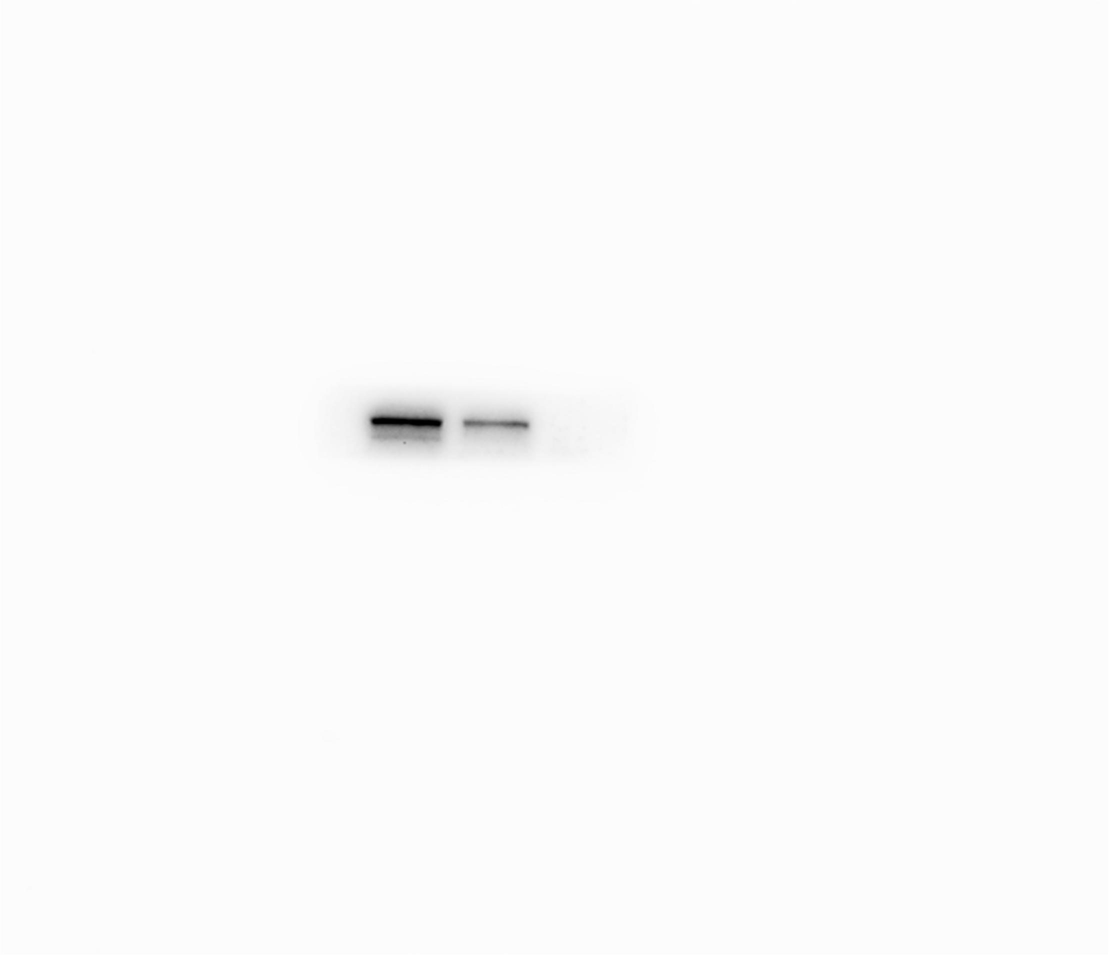

Vimentin

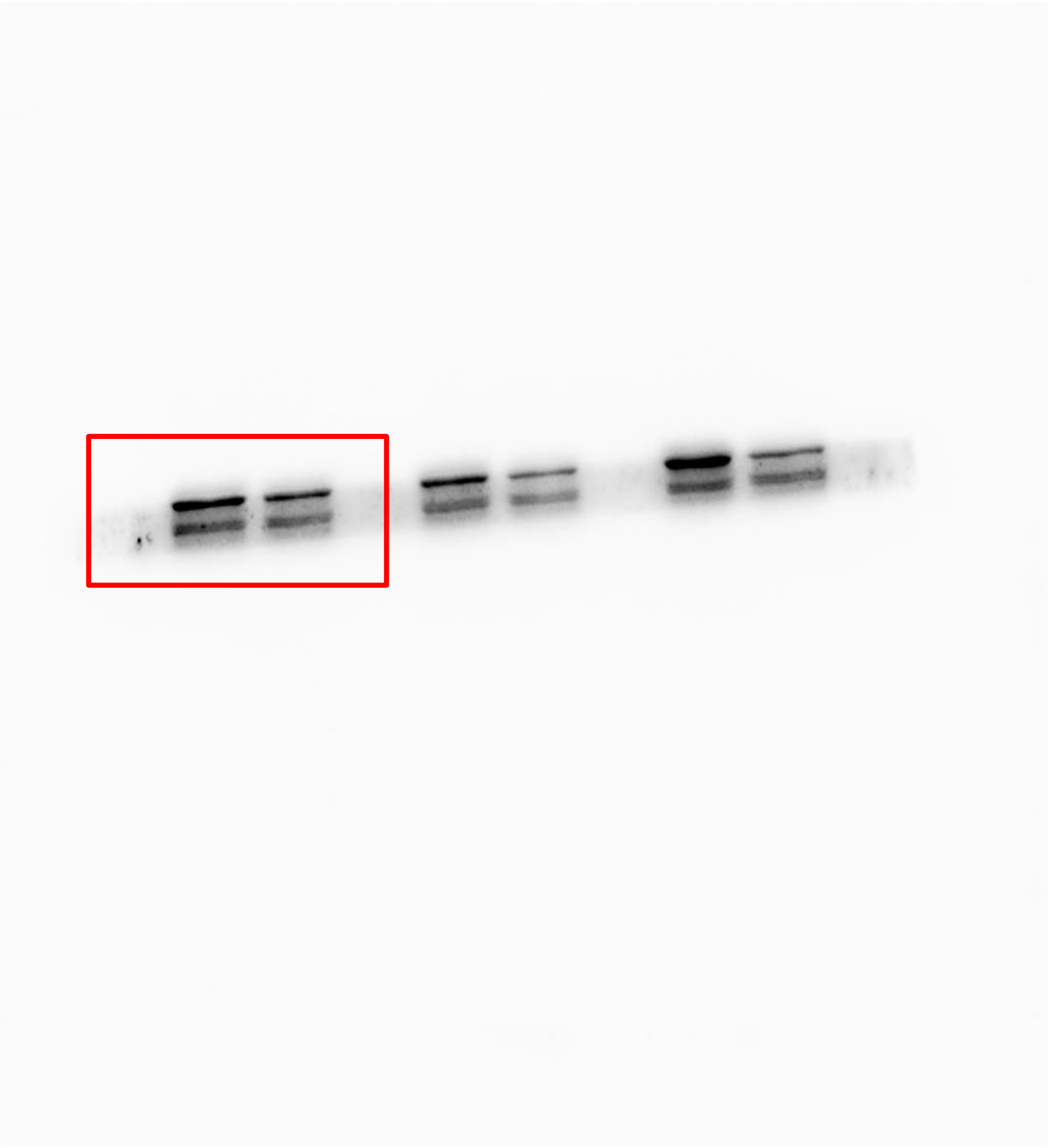

Snail

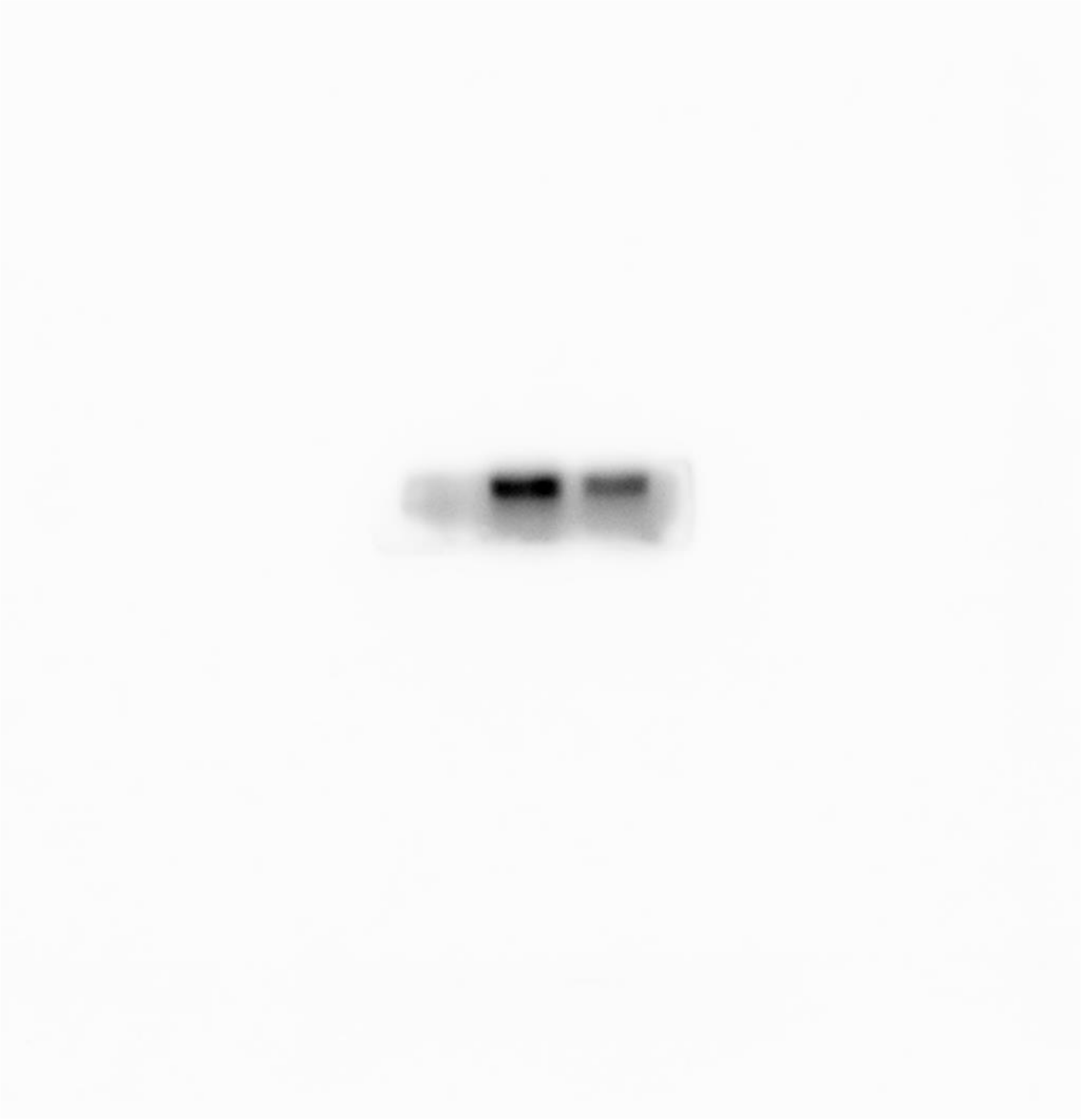

β-ACTIN

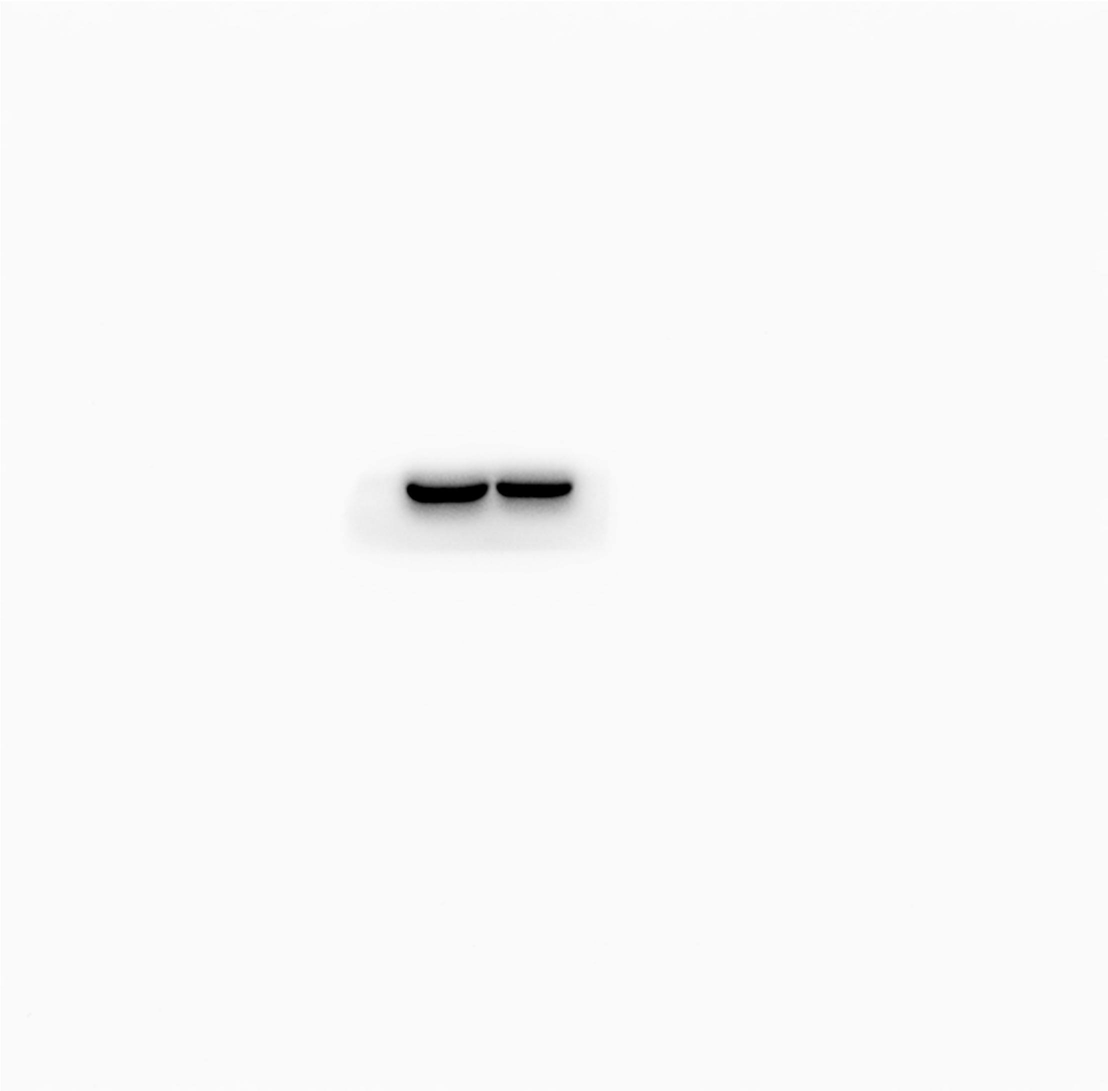

Figure 3_C_MHCC97H

E-Cadherin

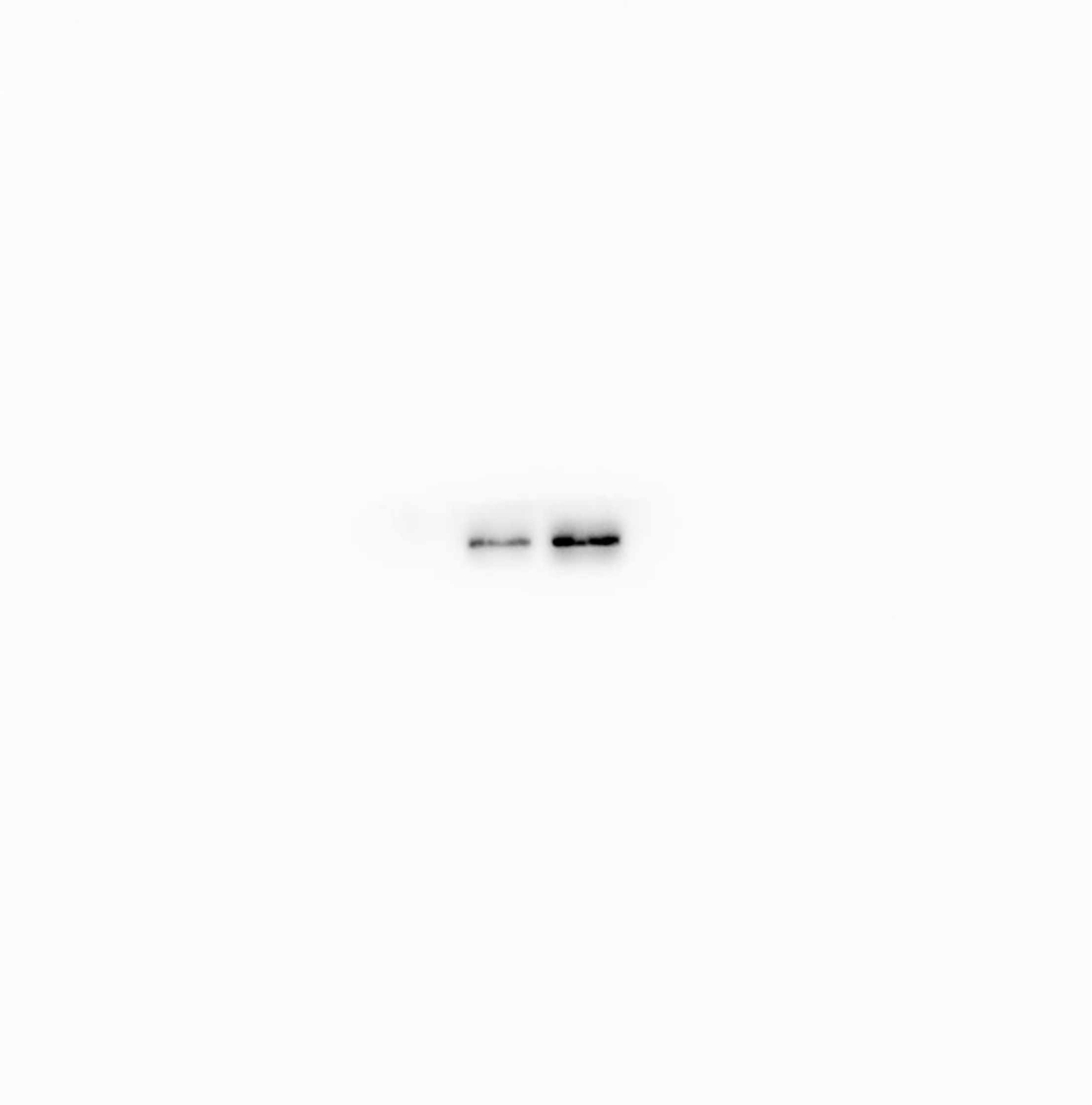

N-Cadherin

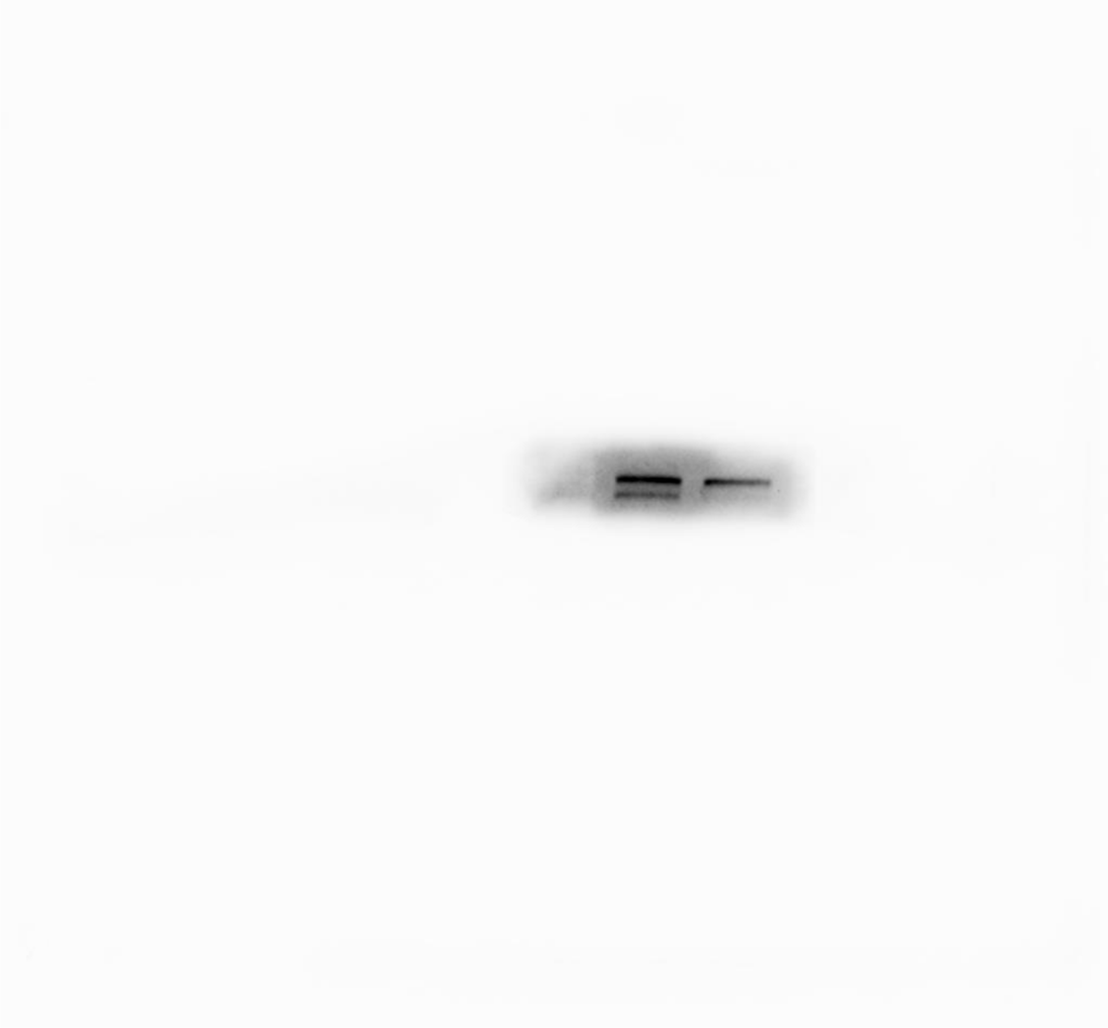

Vimentin

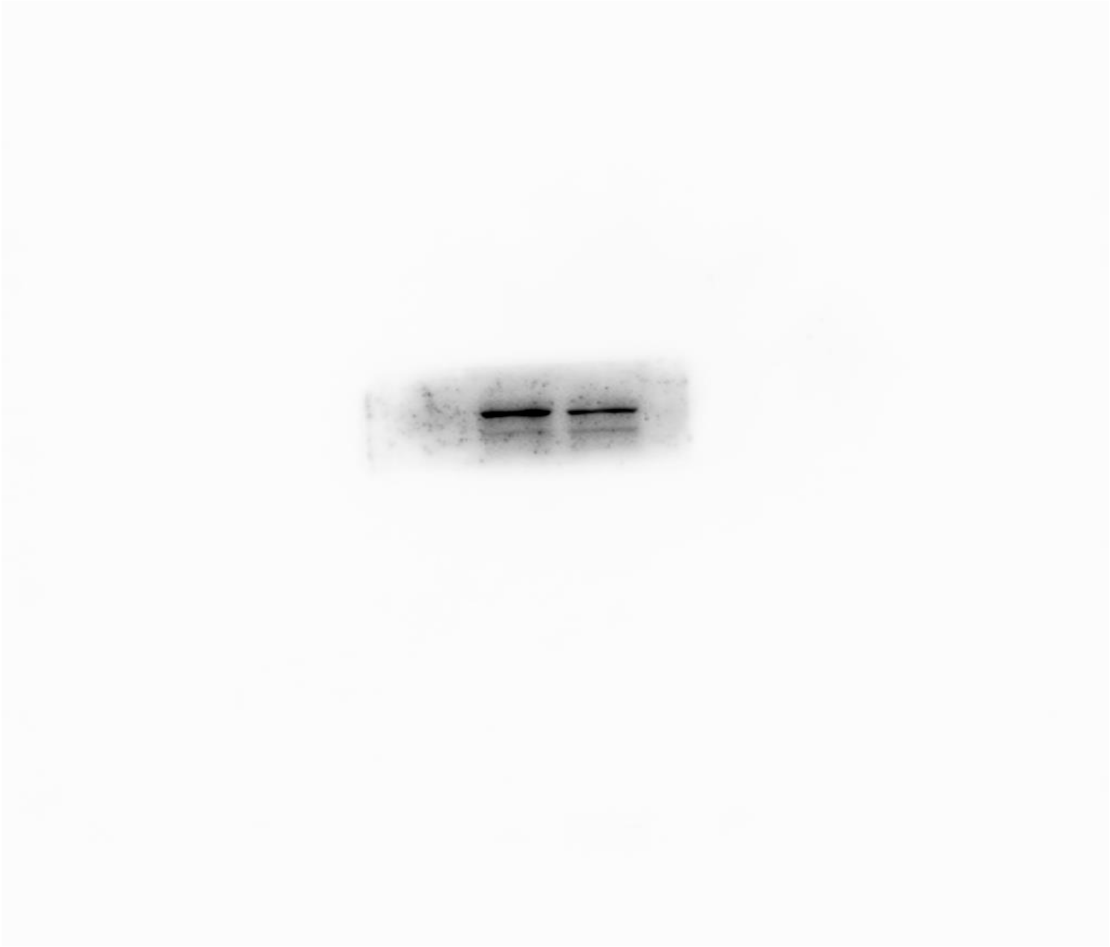

Snail

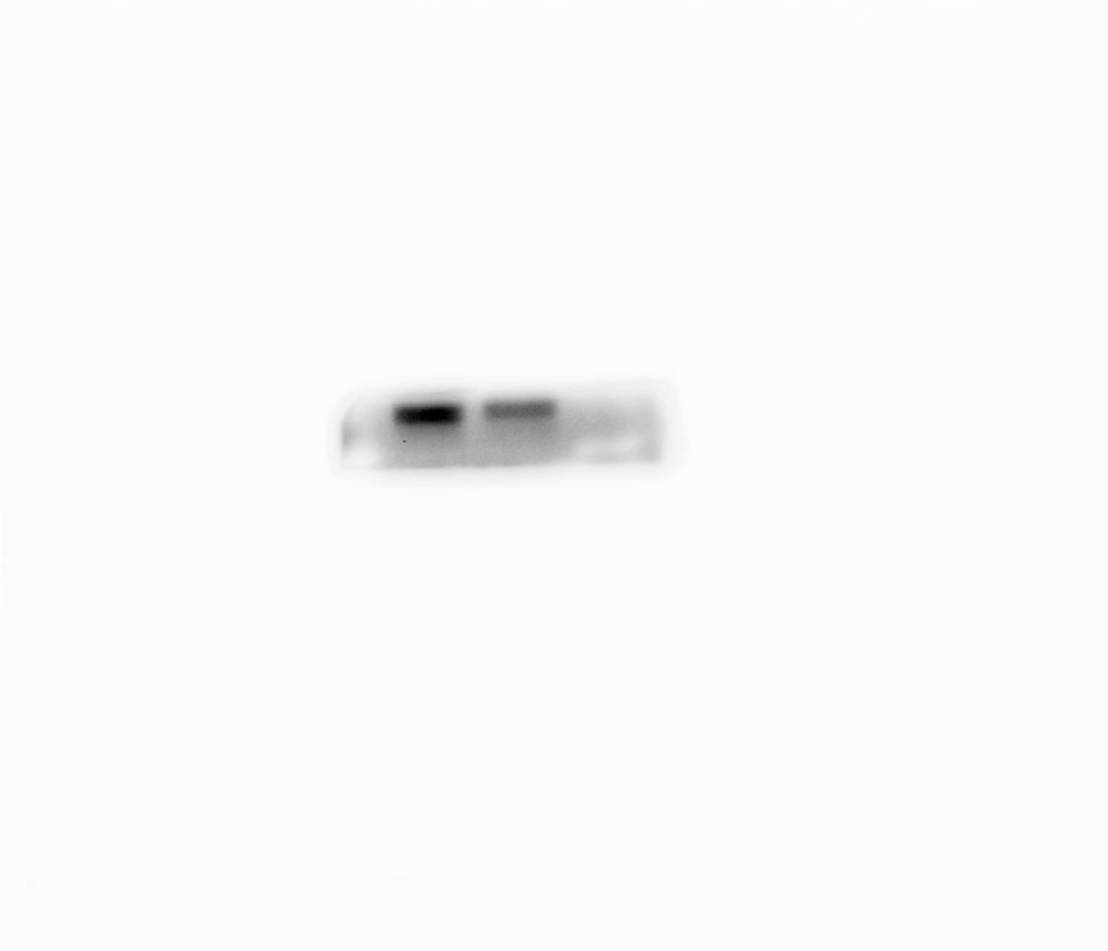

β-ACTIN

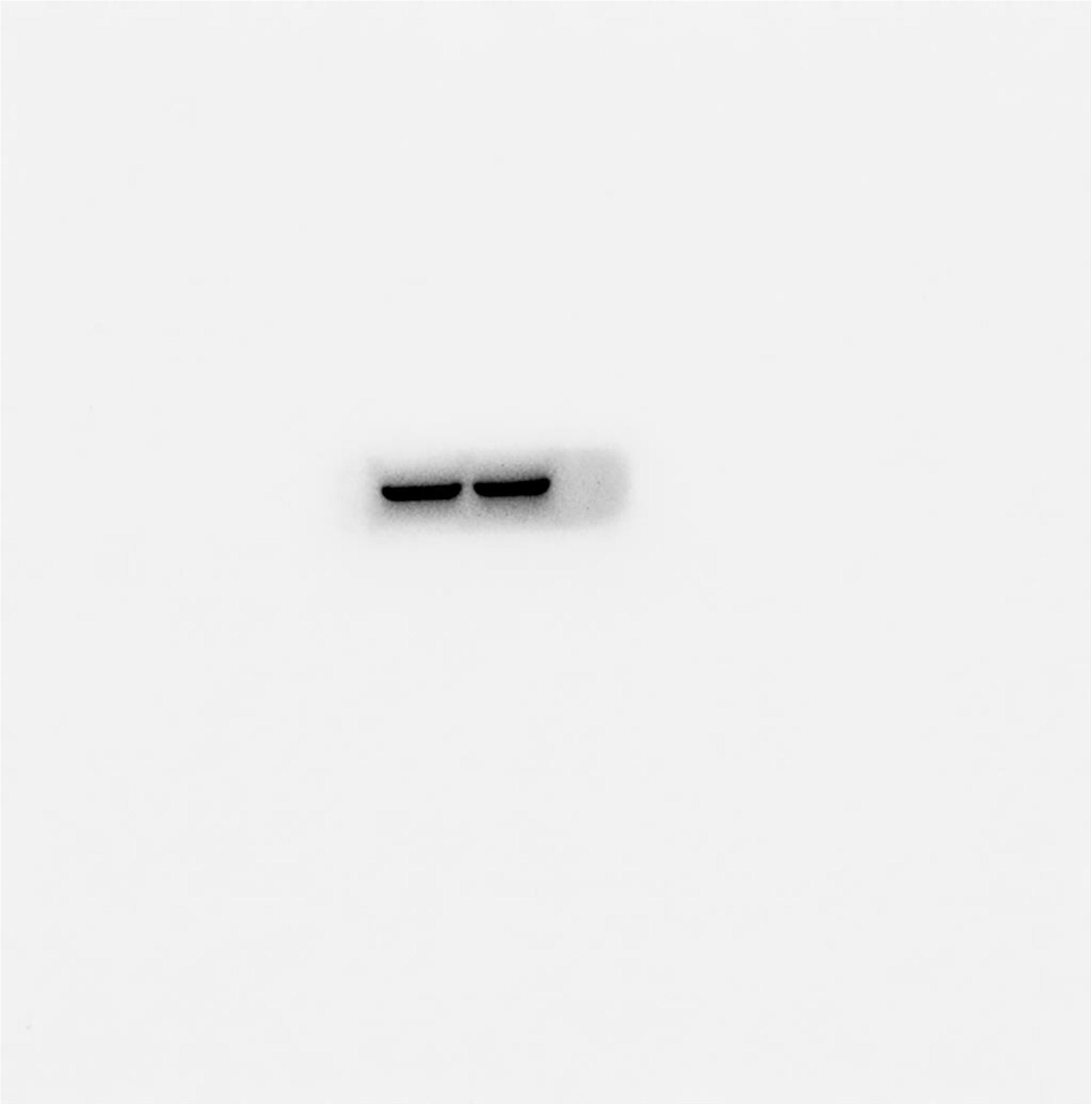

Figure 4_A

HIF-1α

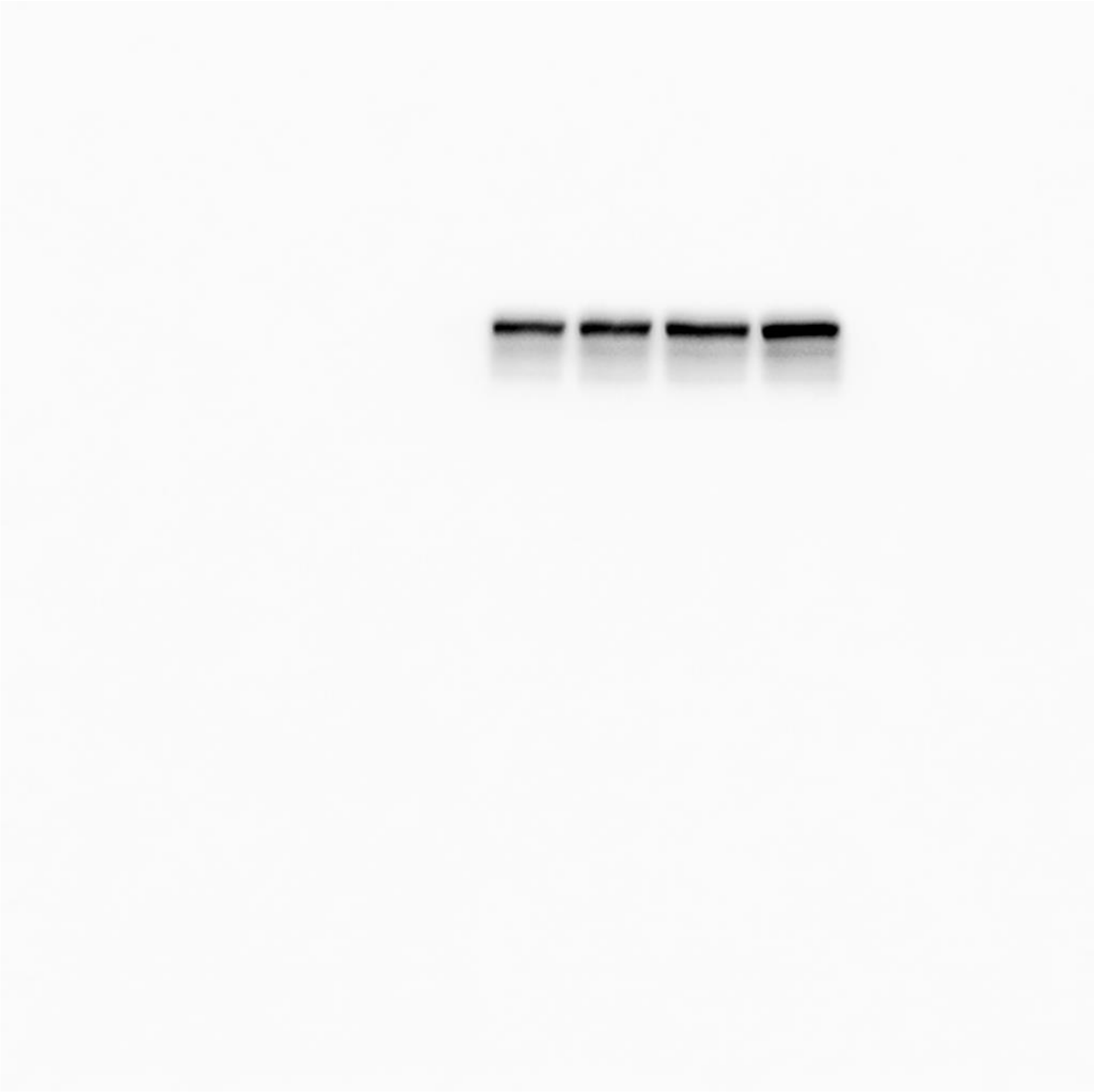

E-Cadherin

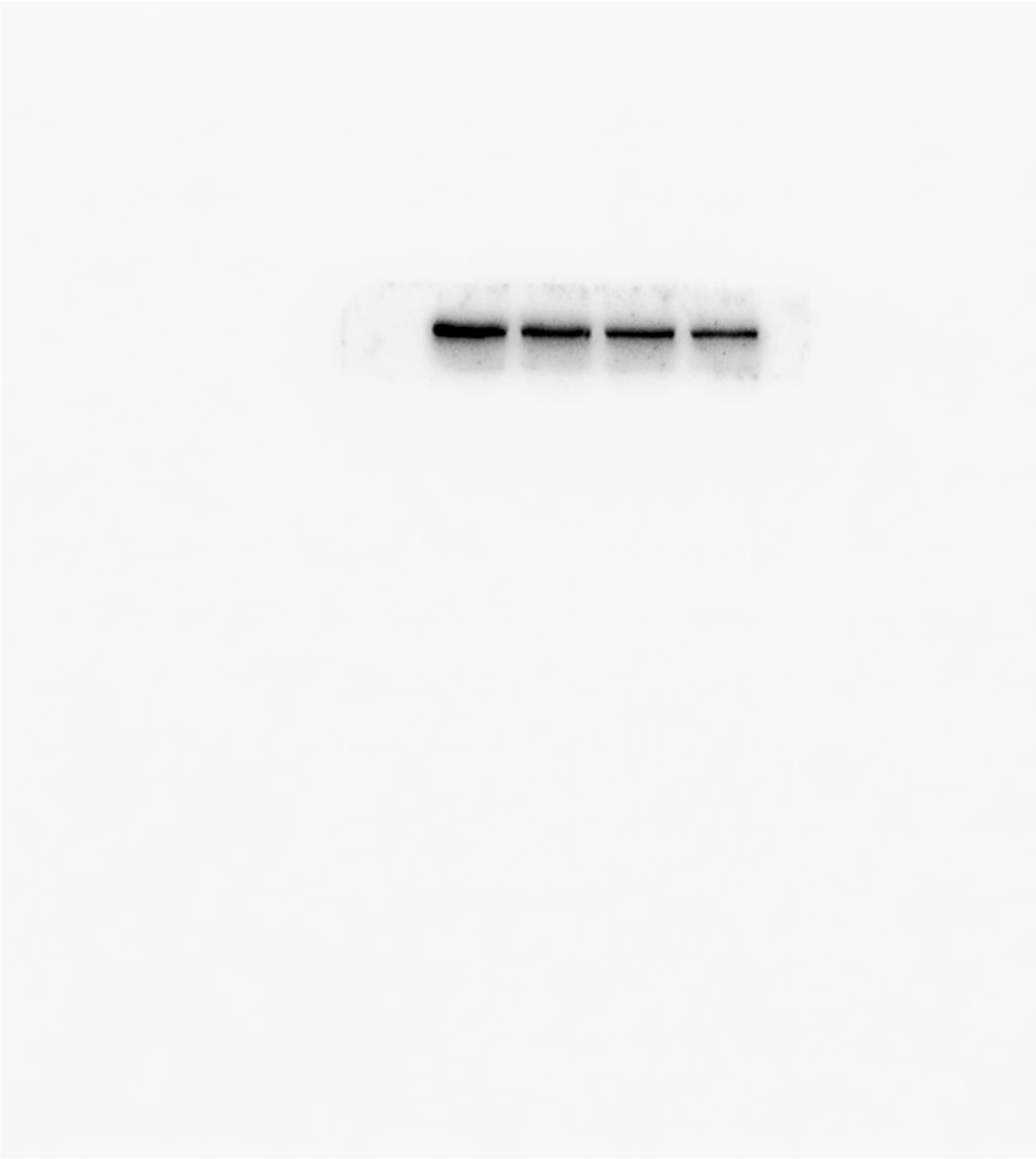

N-Cadherin

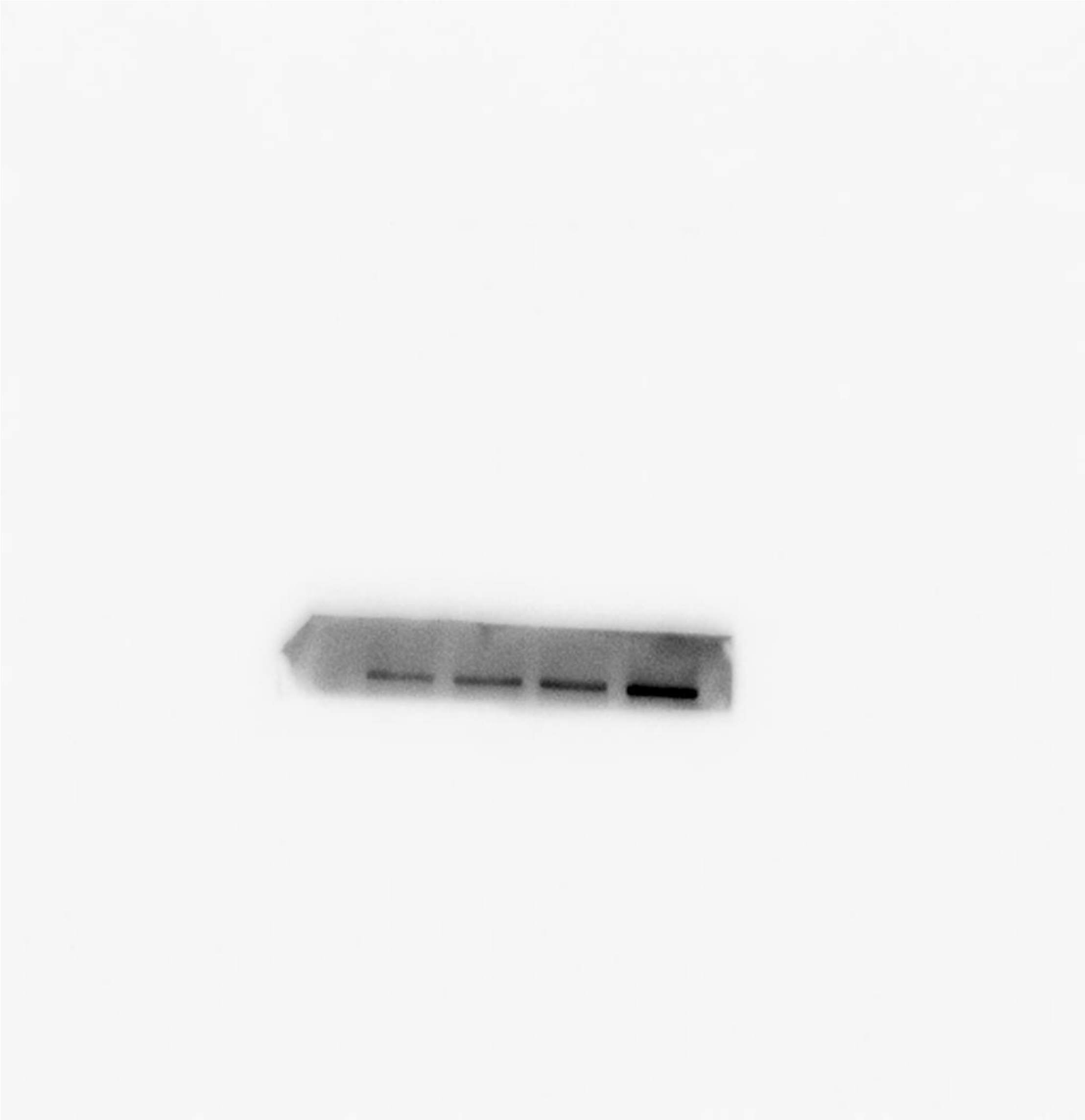

Vimentin

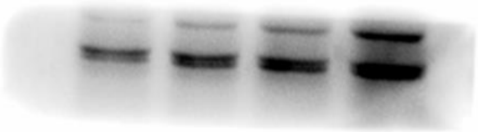

Snail

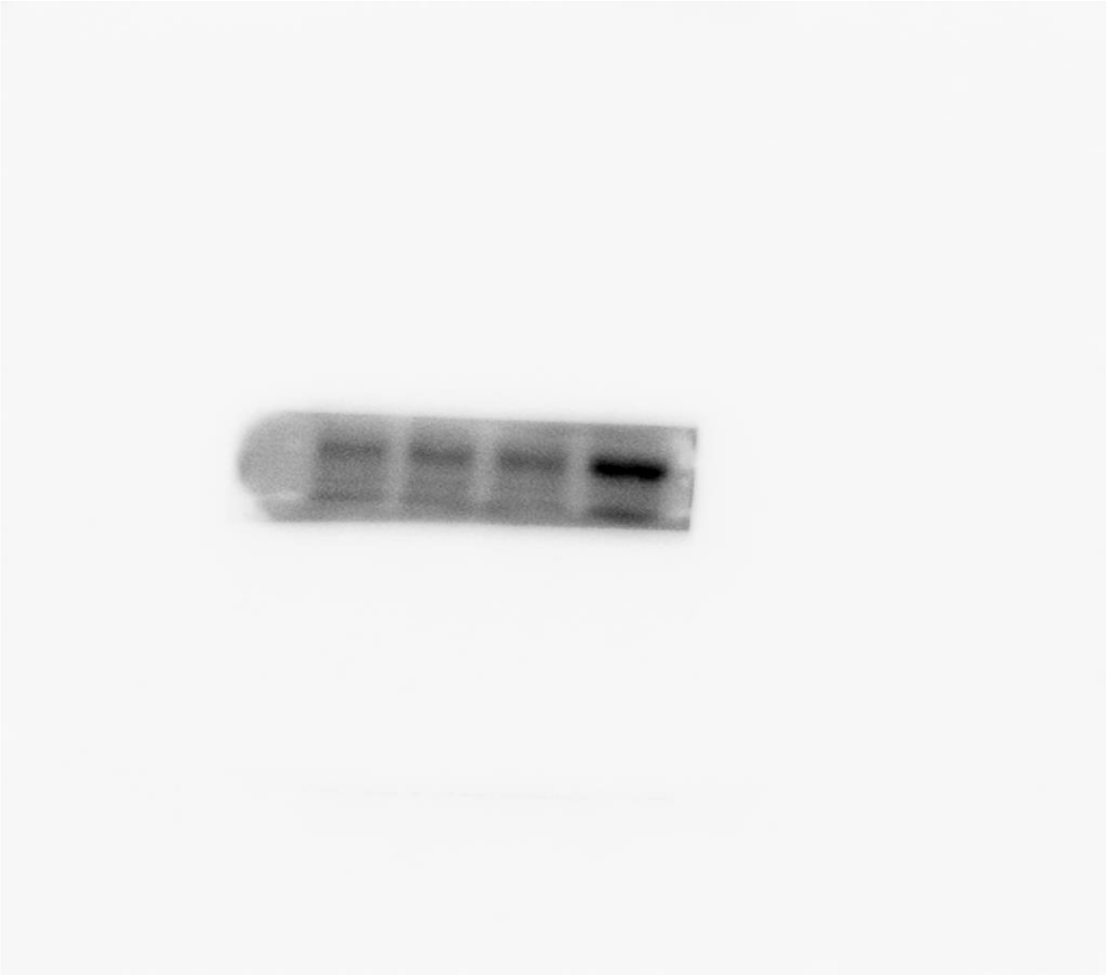

β-ACTIN

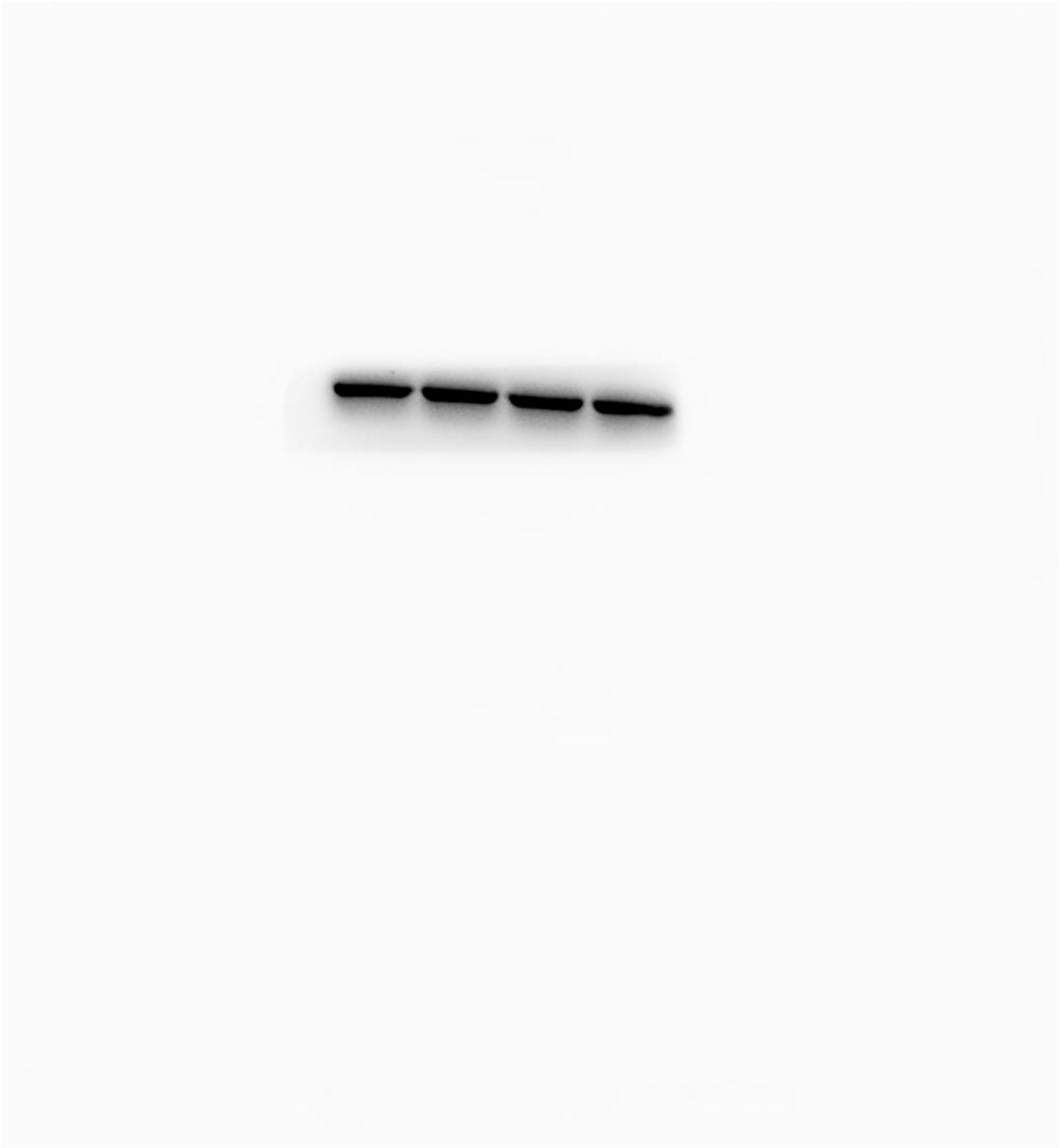

Figure 4_G_MHCC97H

HIF-1α

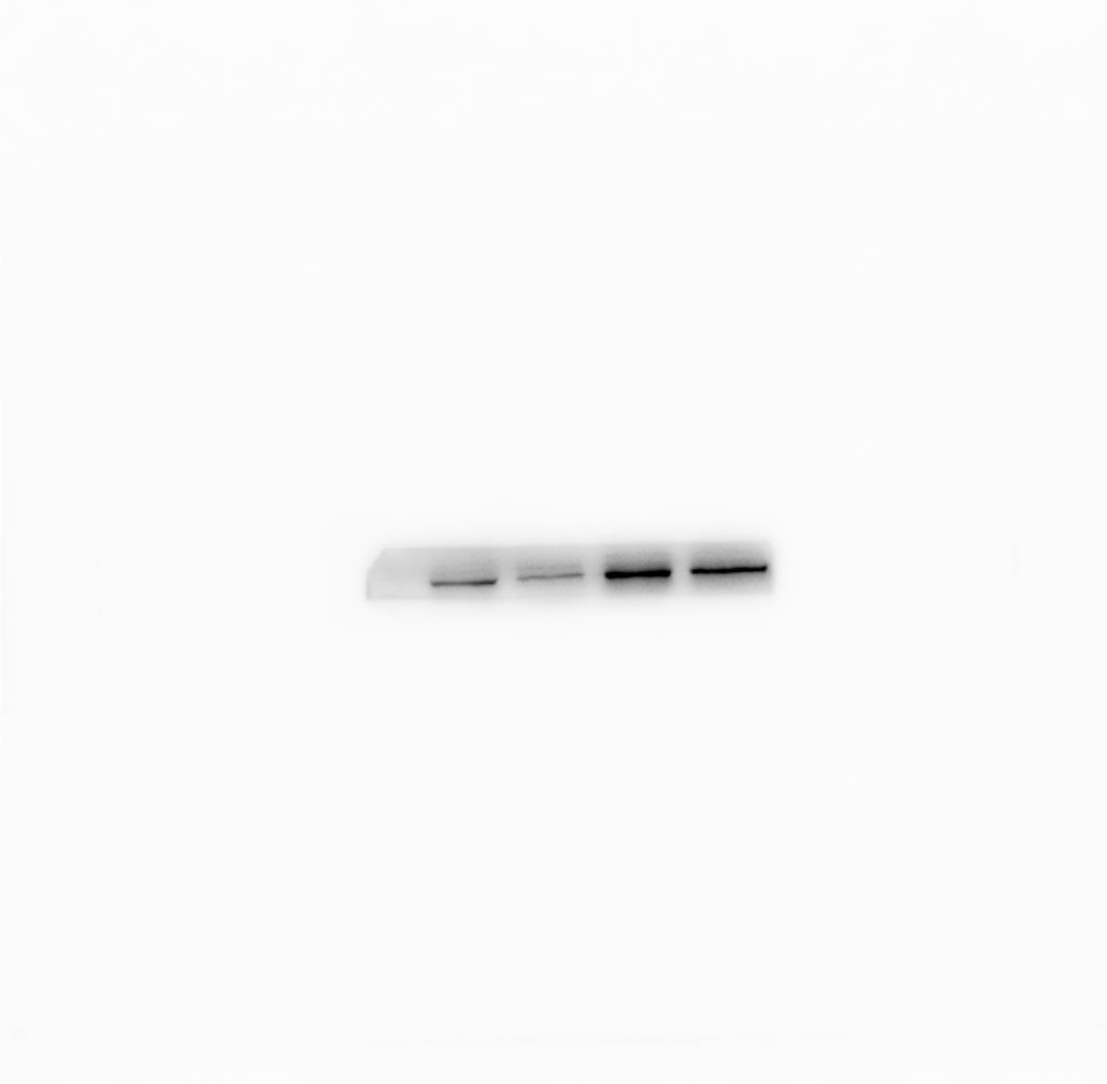

E-Cadherin

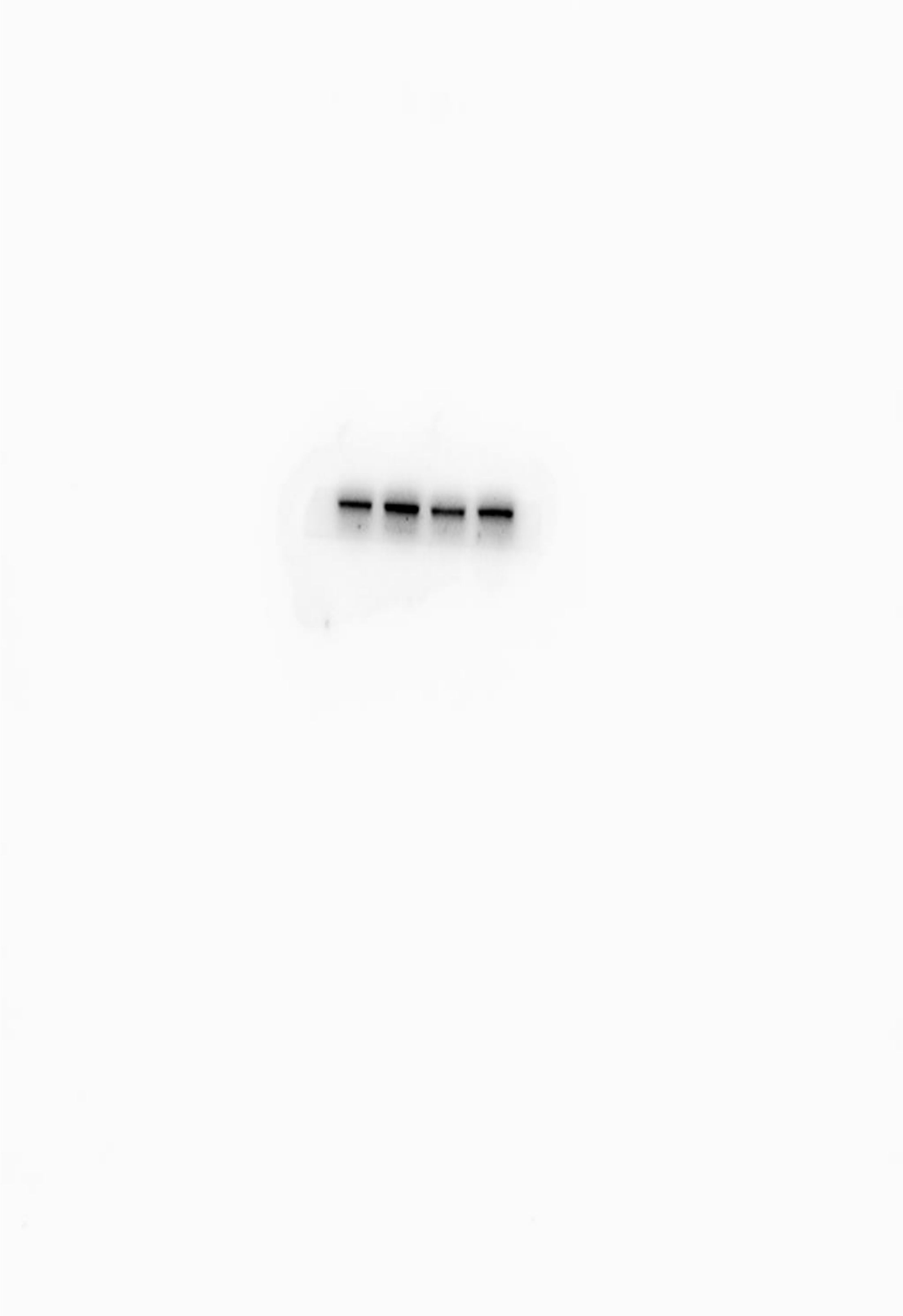

N-Cadherin

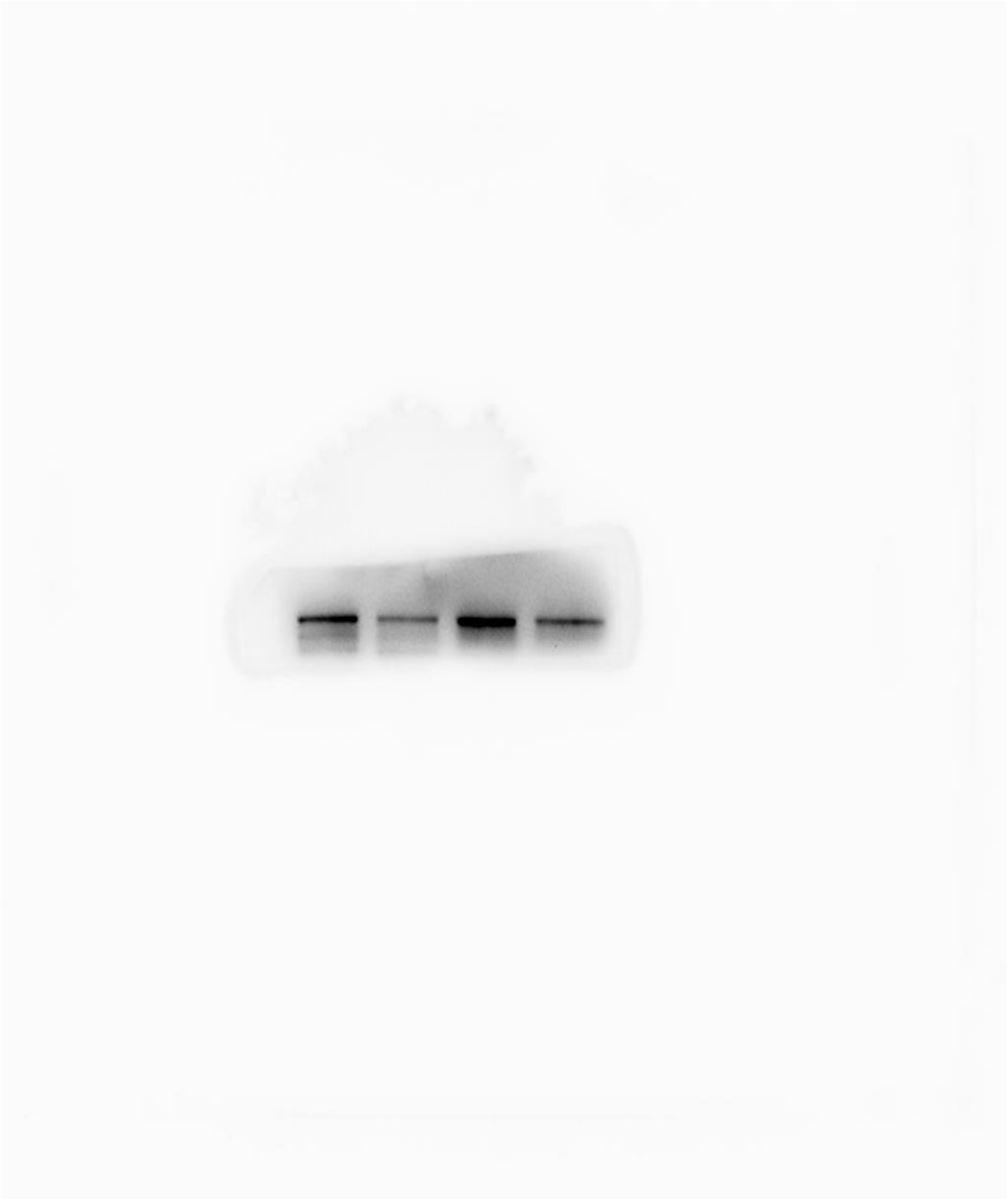

Vimentin

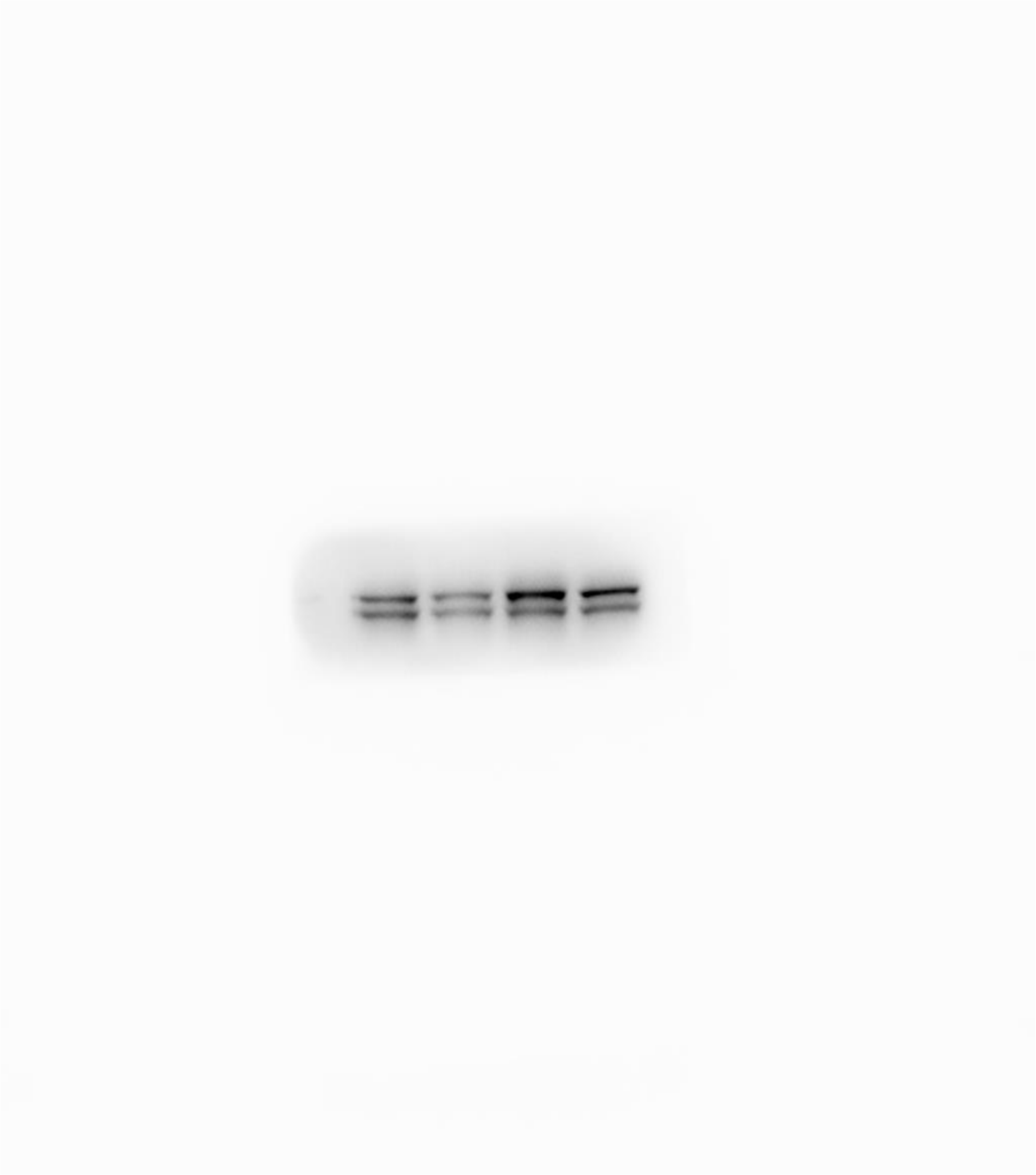

Snail

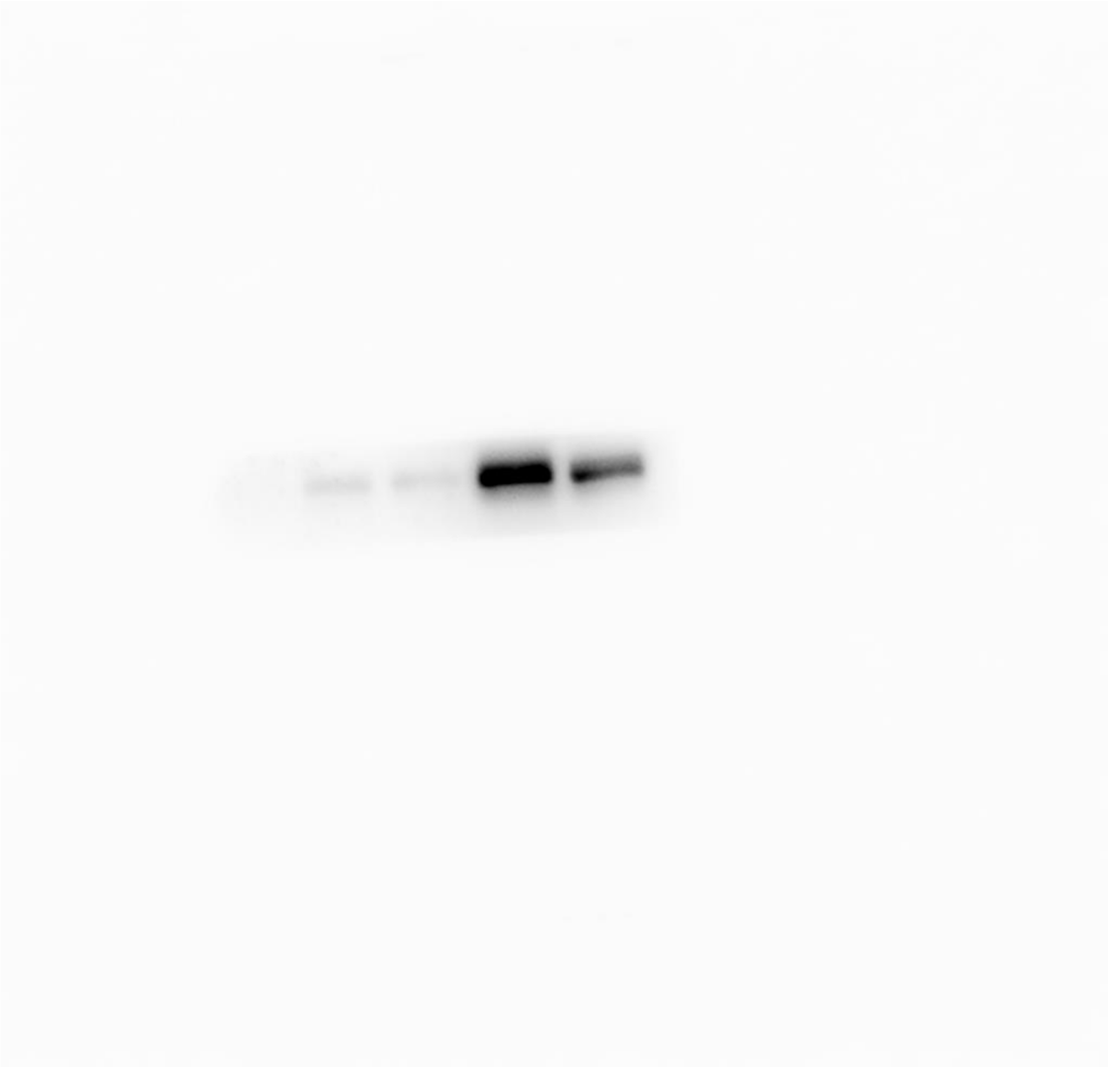

β-ACTIN

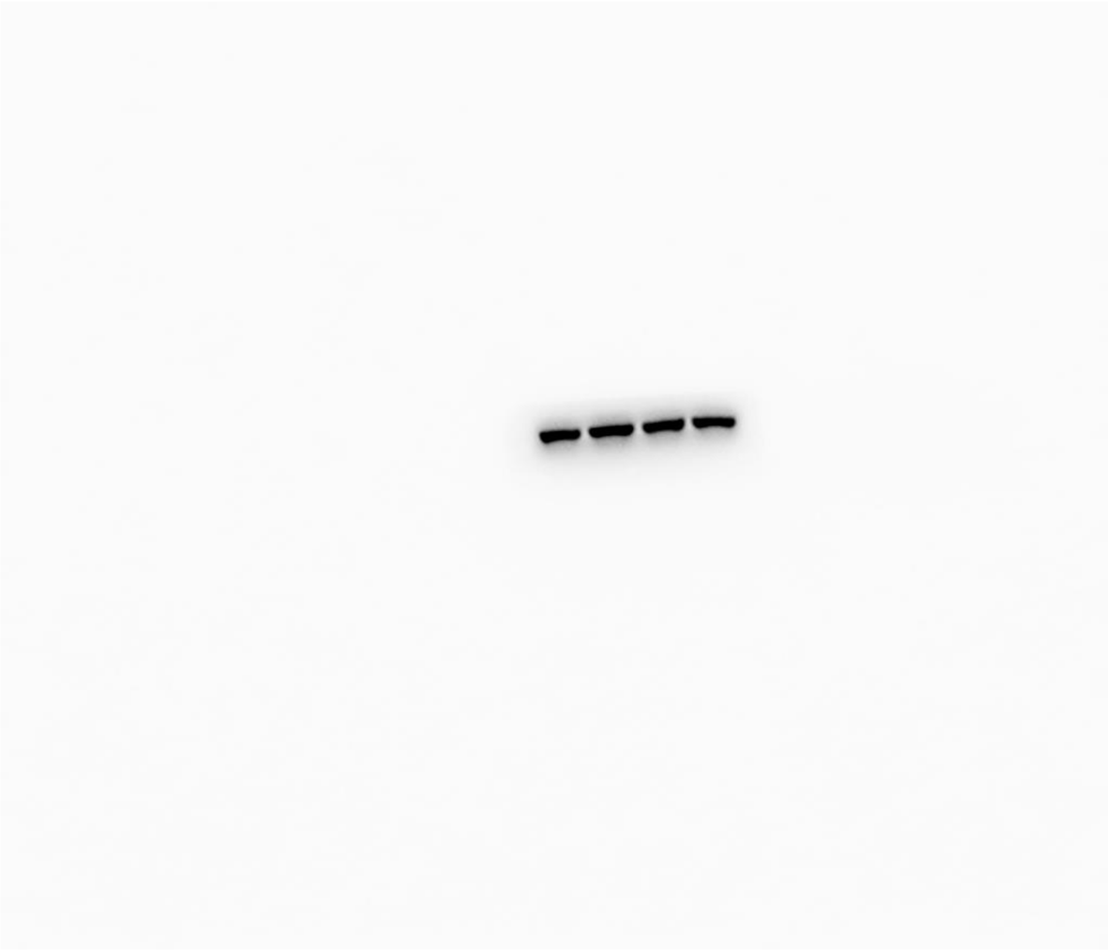

Figure 4_H_HCCLM3

HIF-1α

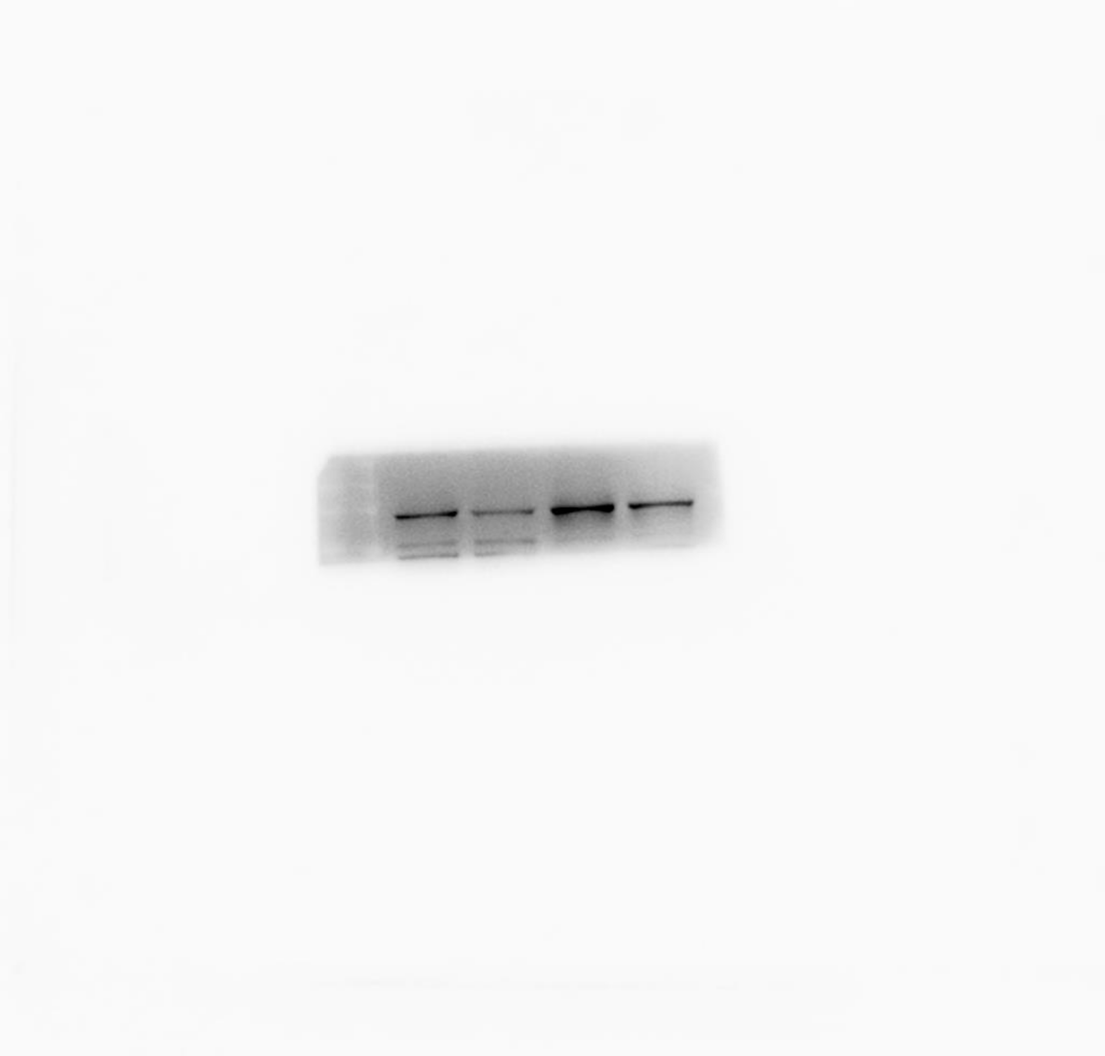

E-Cadherin

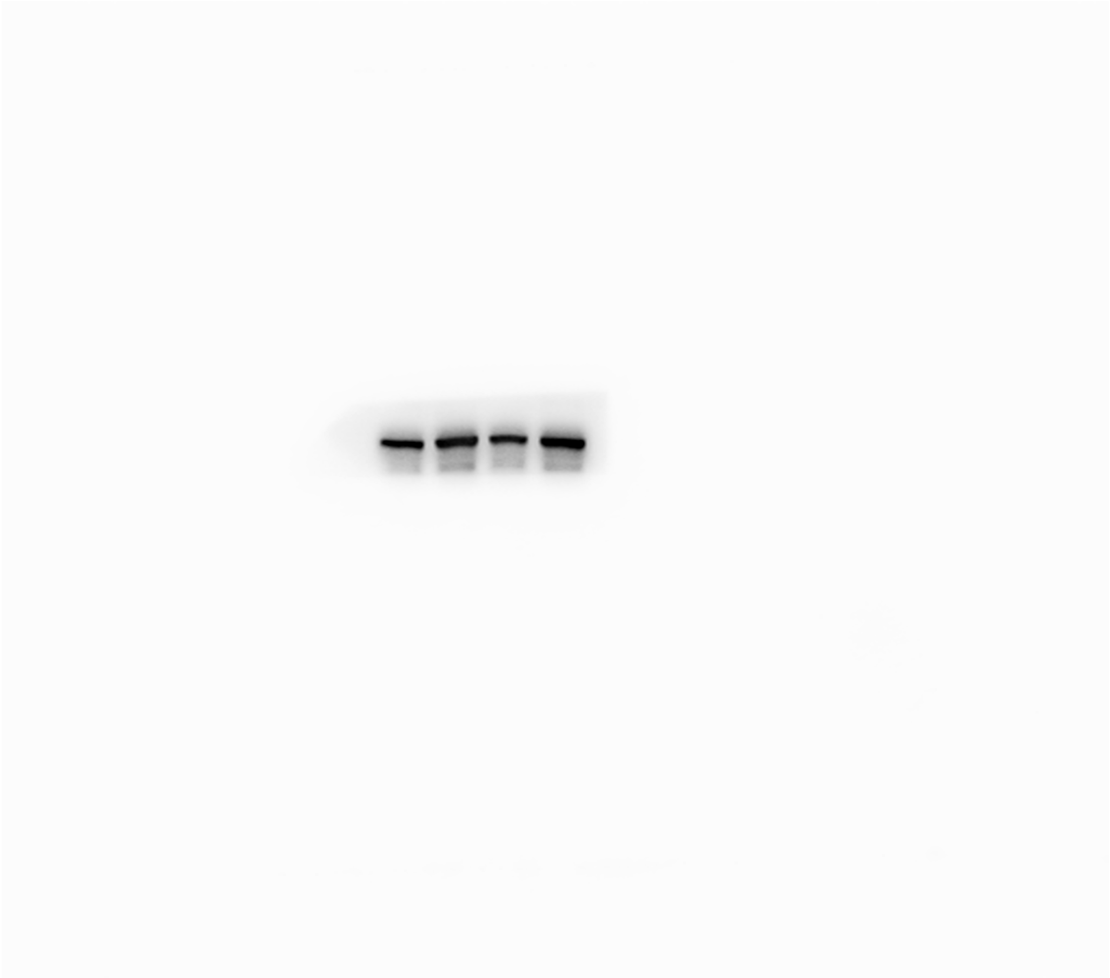

N-Cadherin

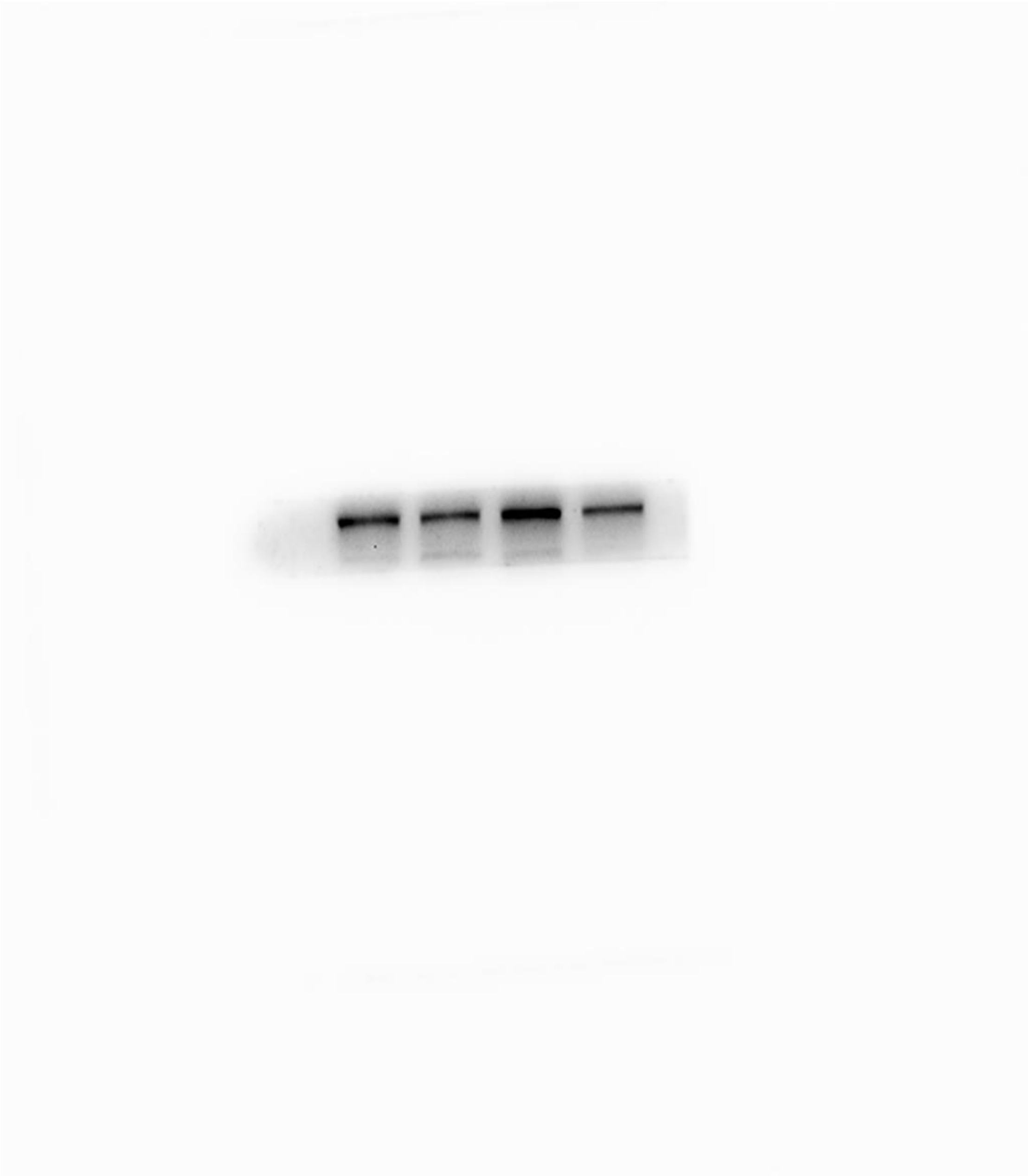

Vimentin

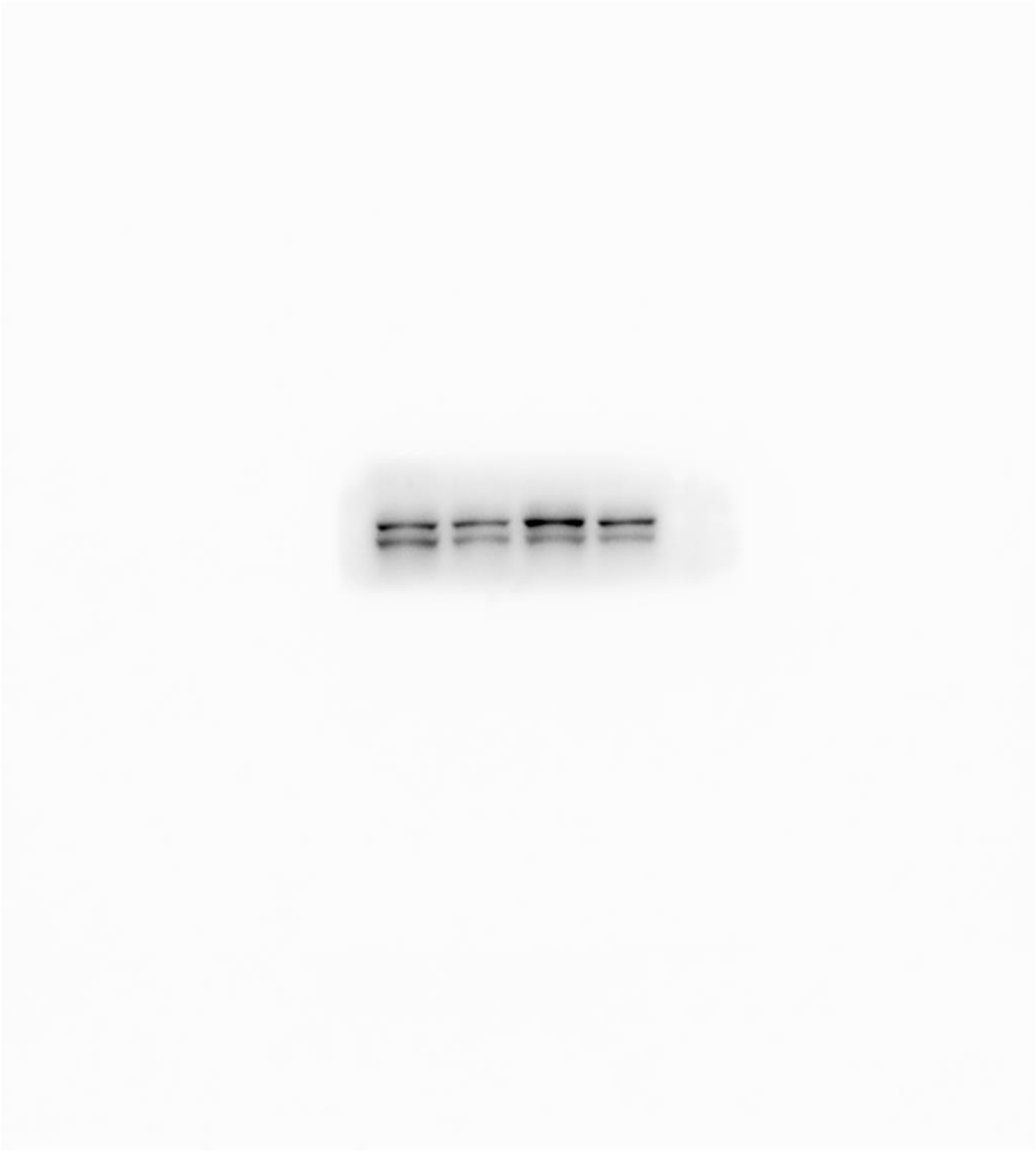

Snail

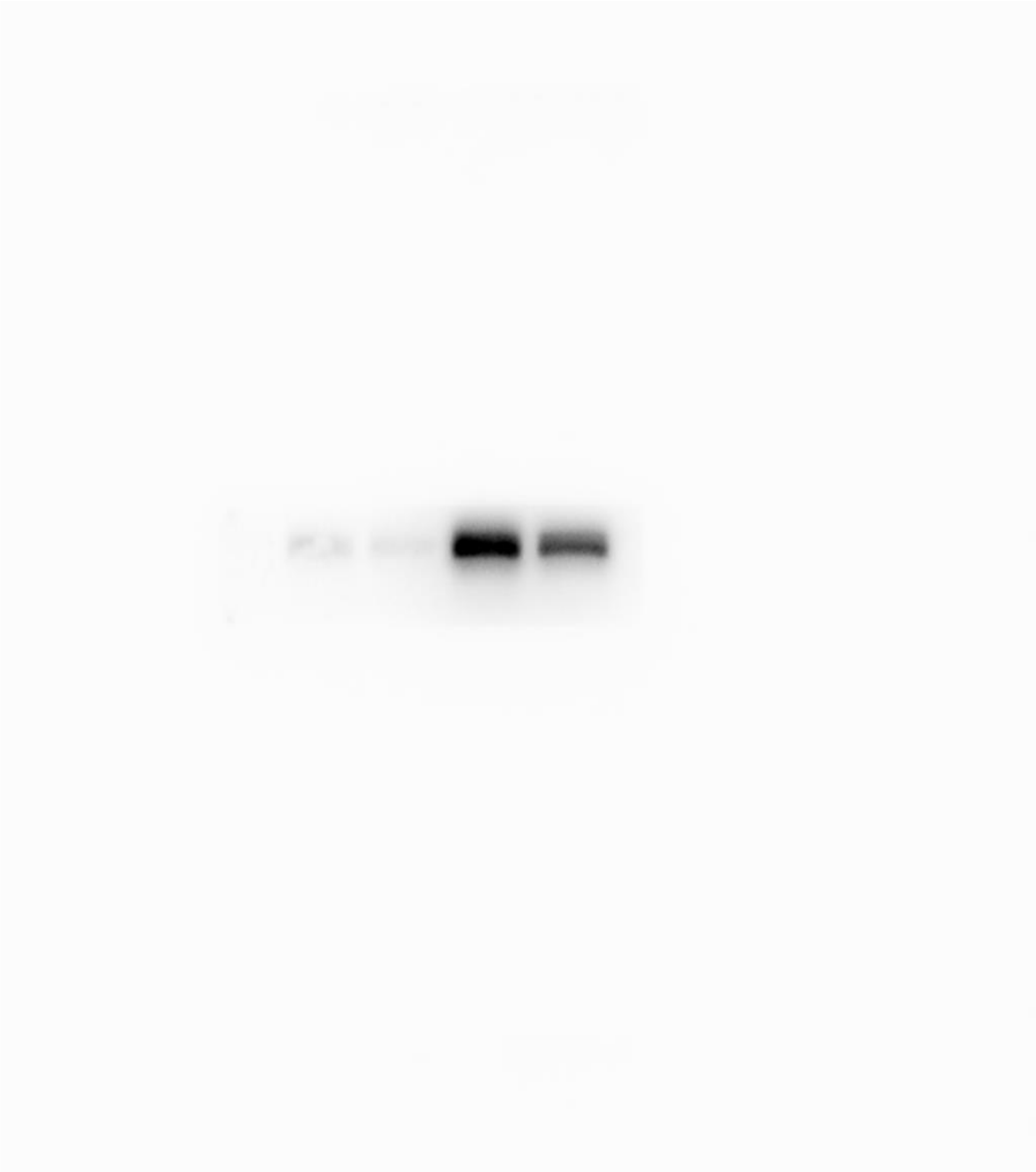

β-ACTIN

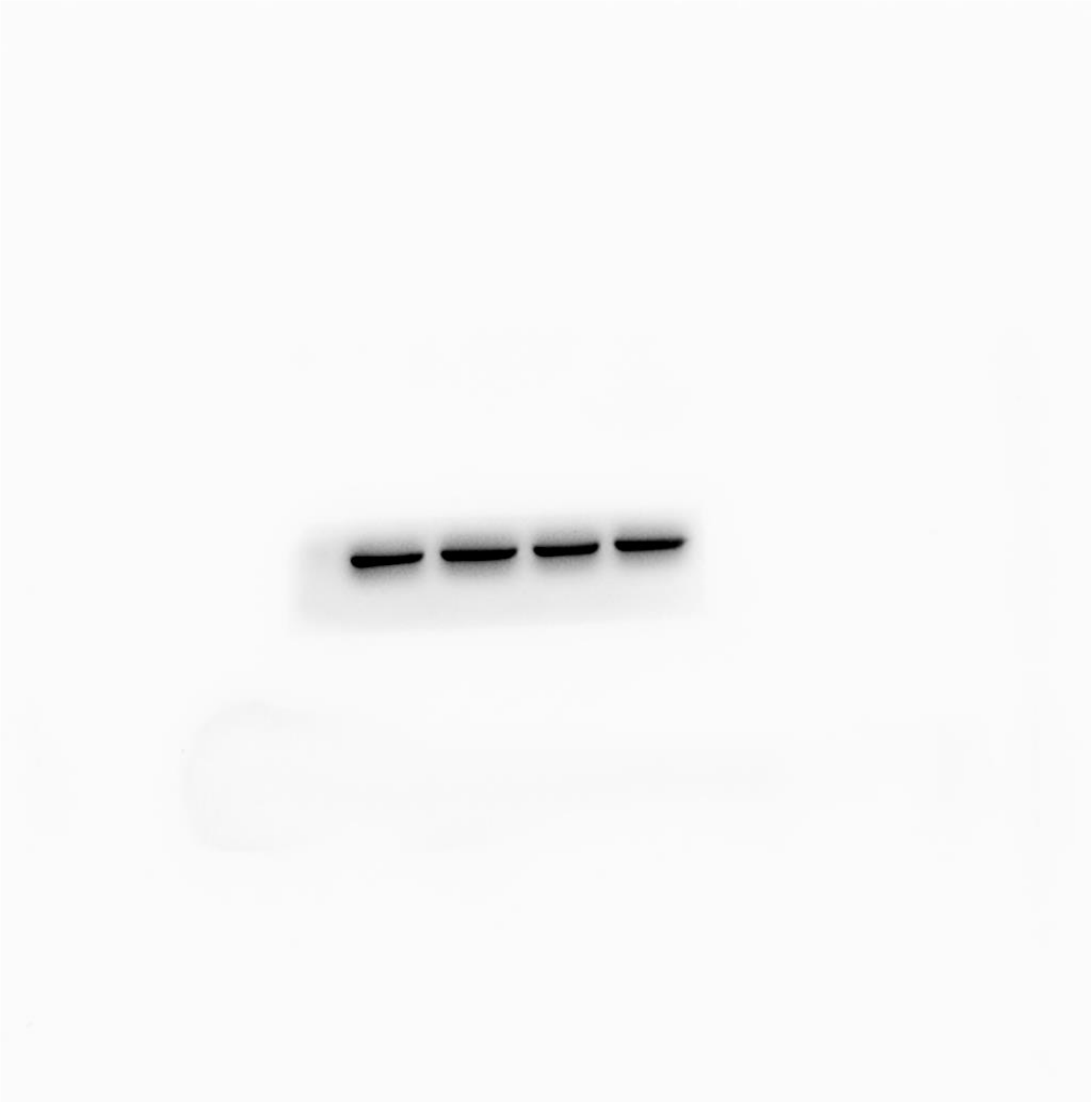

Figure 6_A_LY294002_HUH7

P-AKT(473)

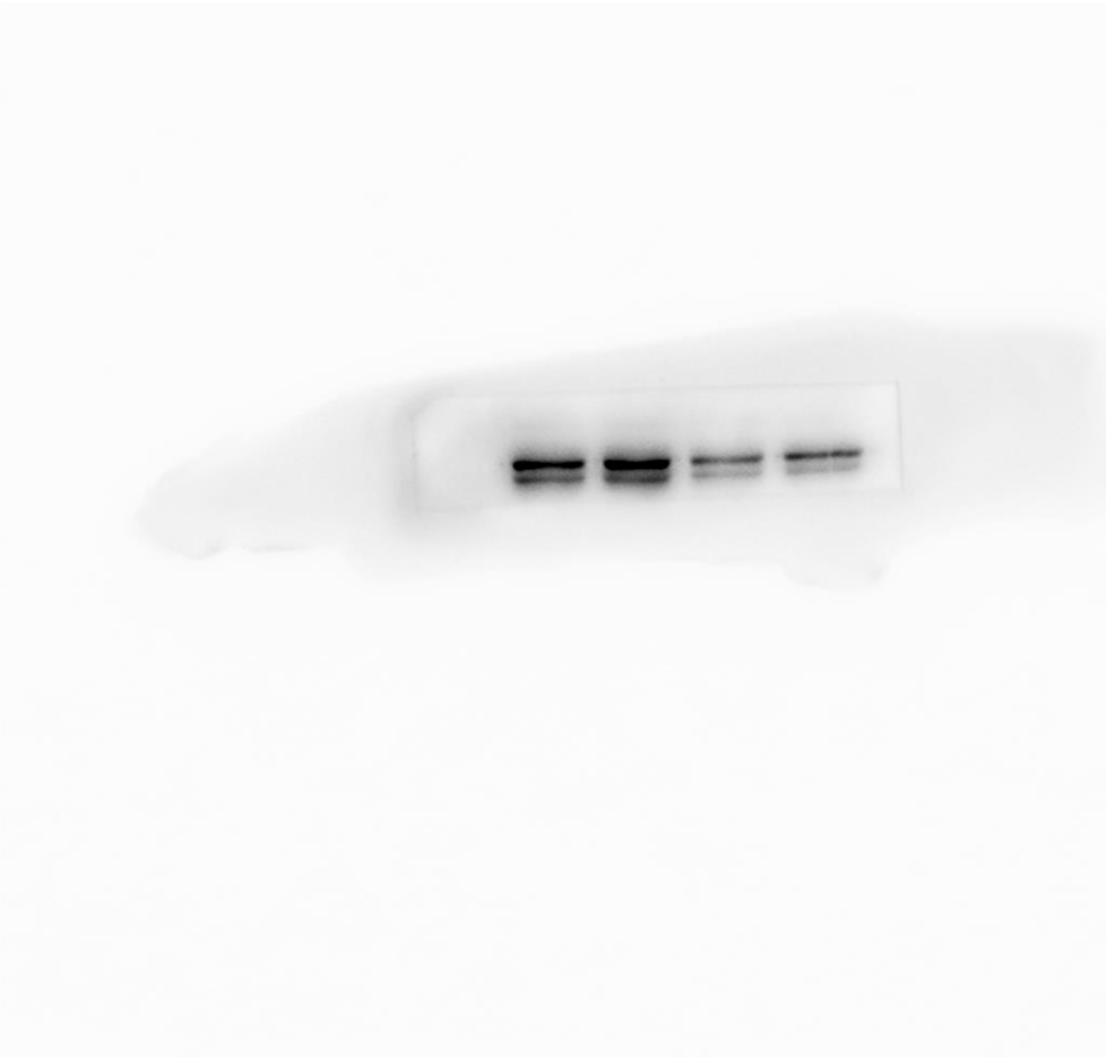

P-MTOR(2448)

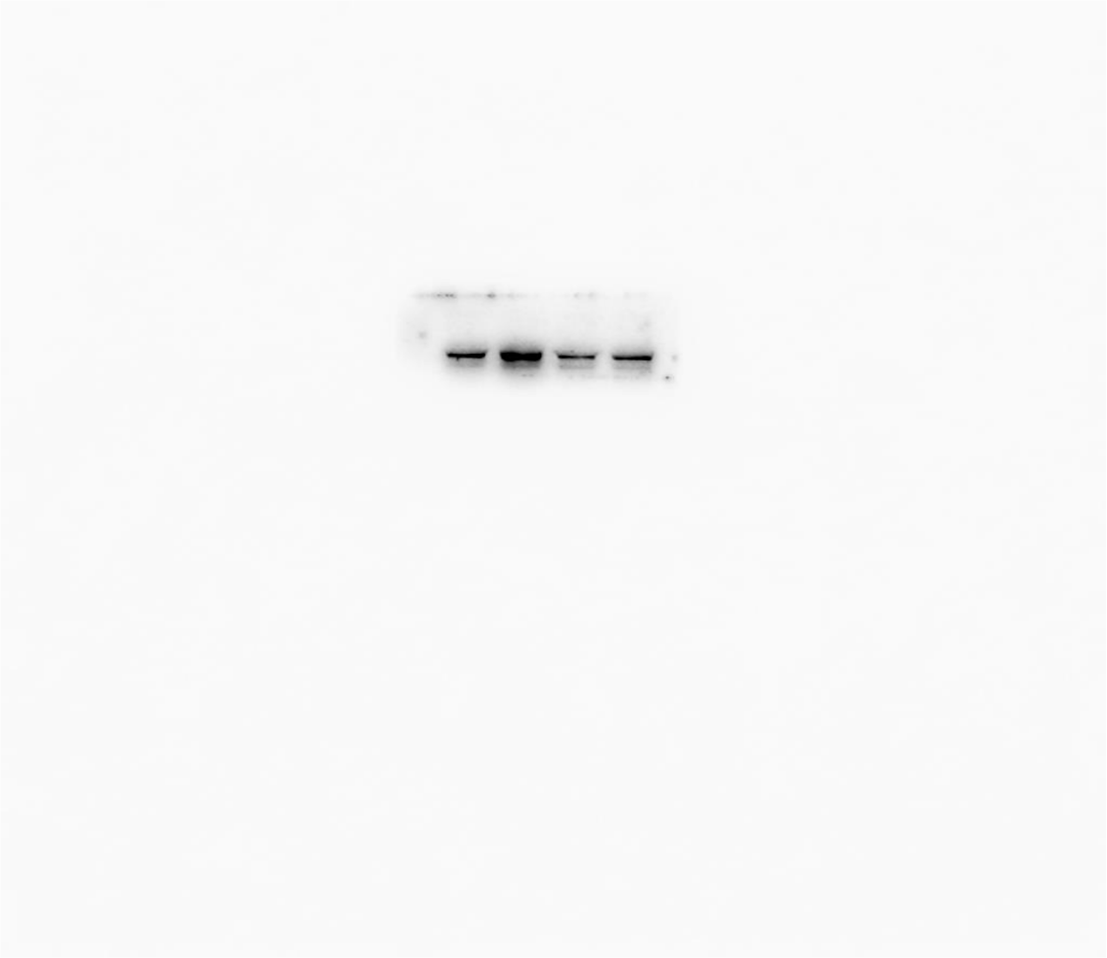

HIF-1α

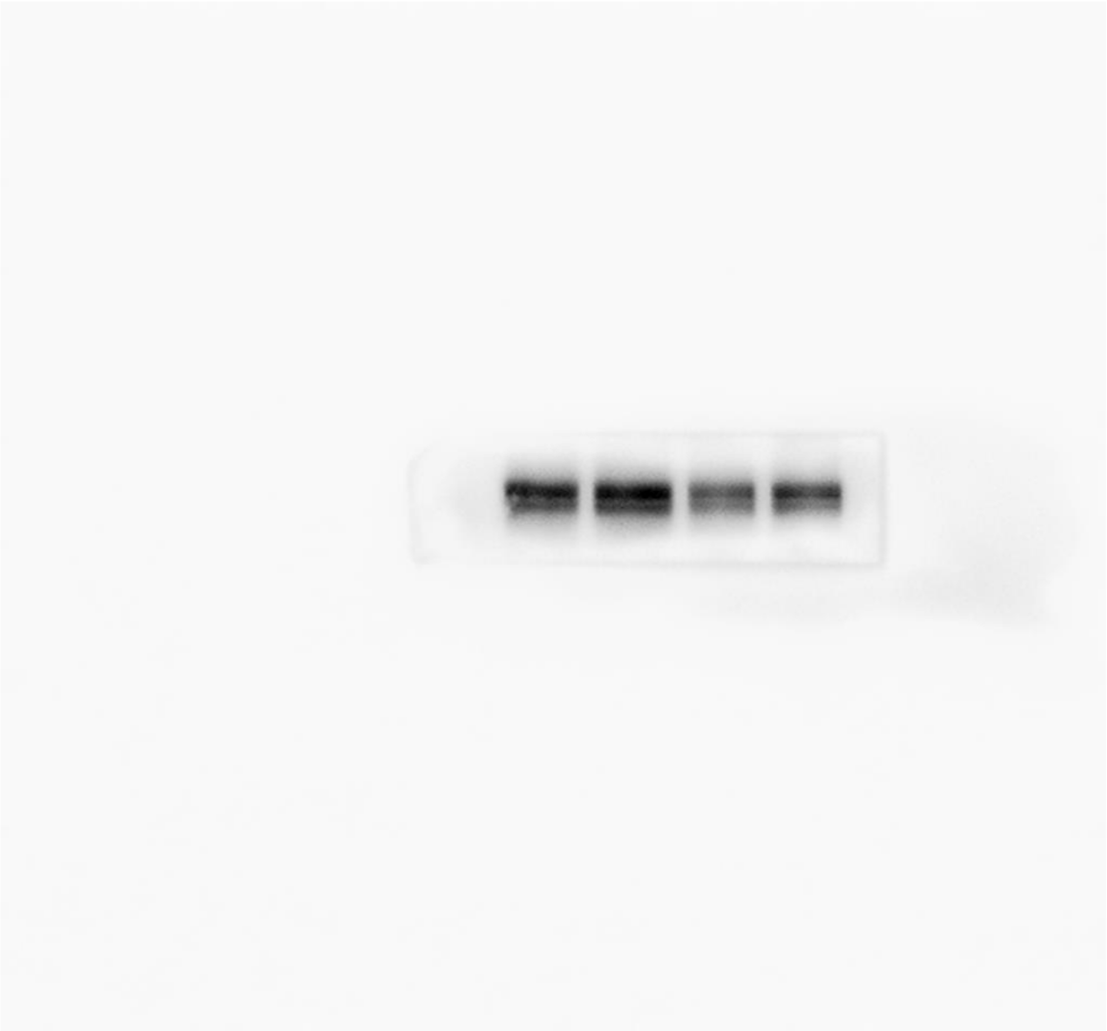

β-ACTIN

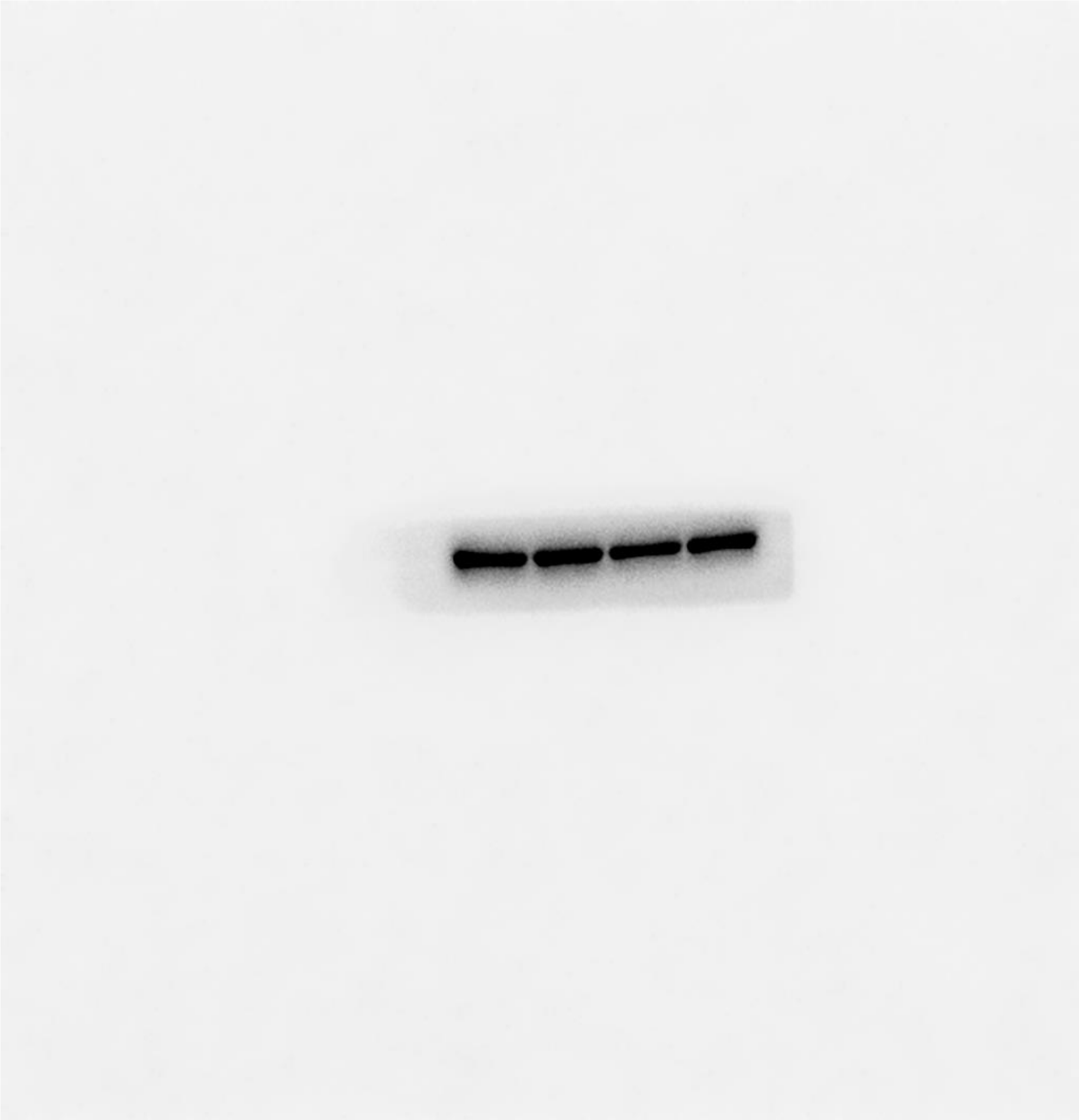

Figure 6_A_LY294002_HEP3B

P-AKT(473)

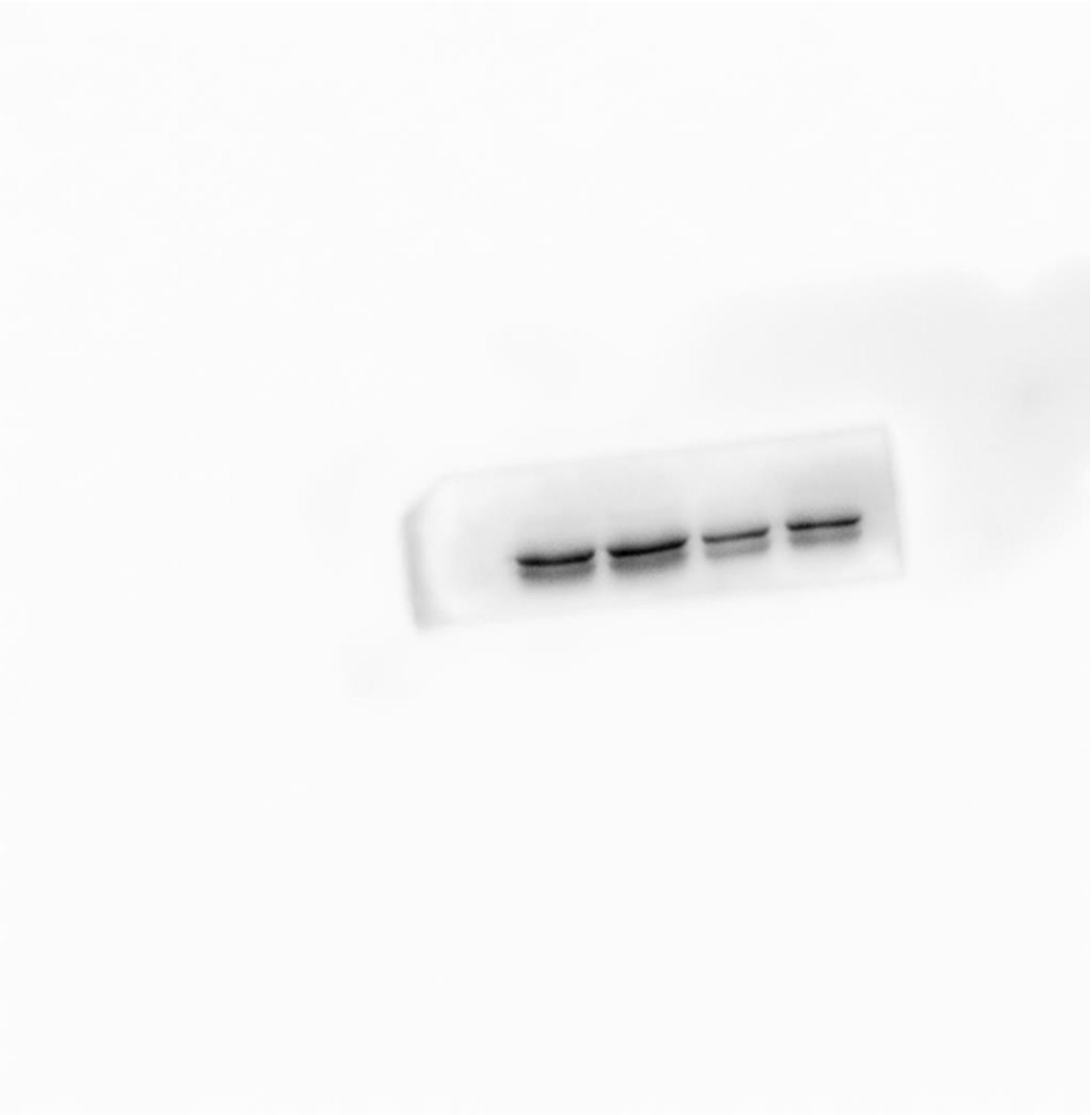

P-MTOR(2448)

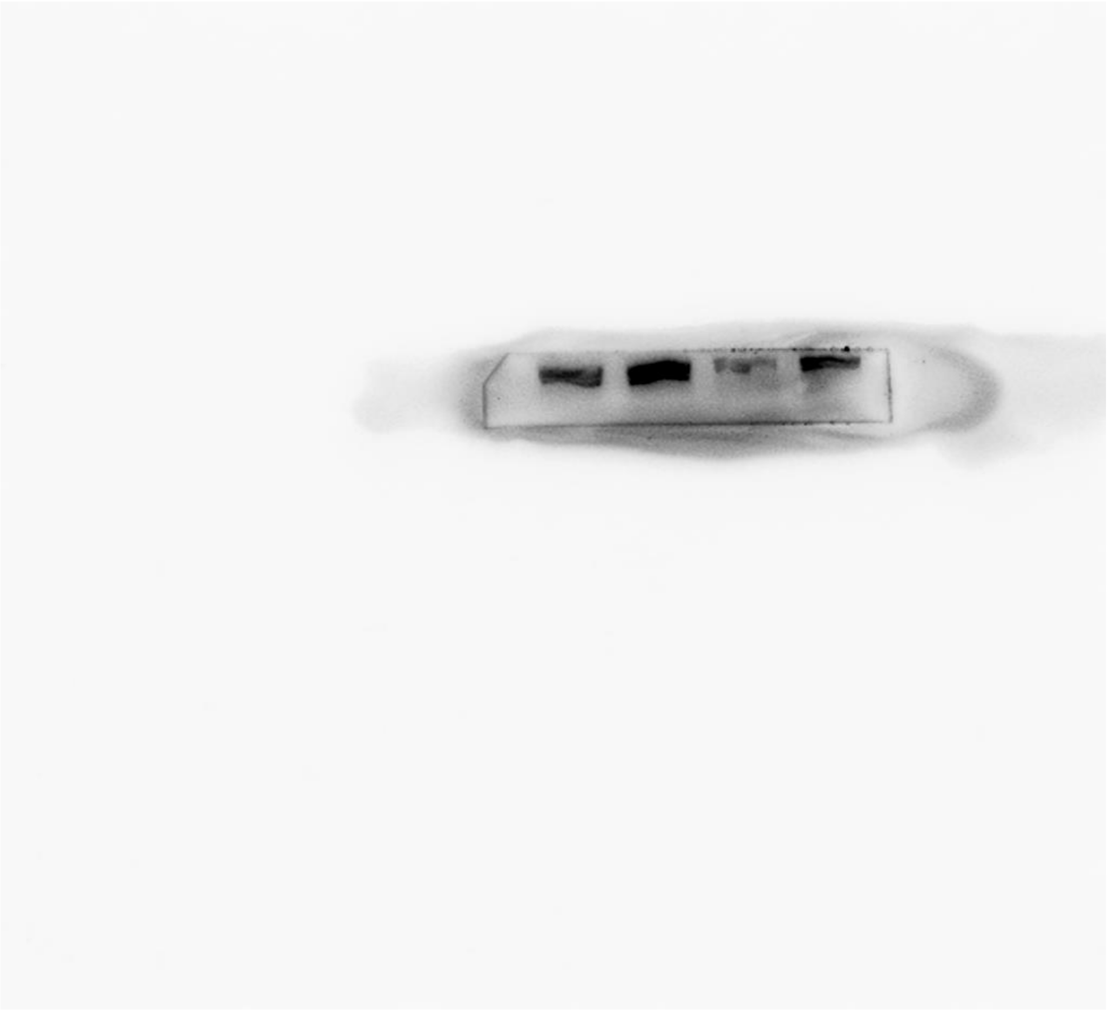

HIF-1α

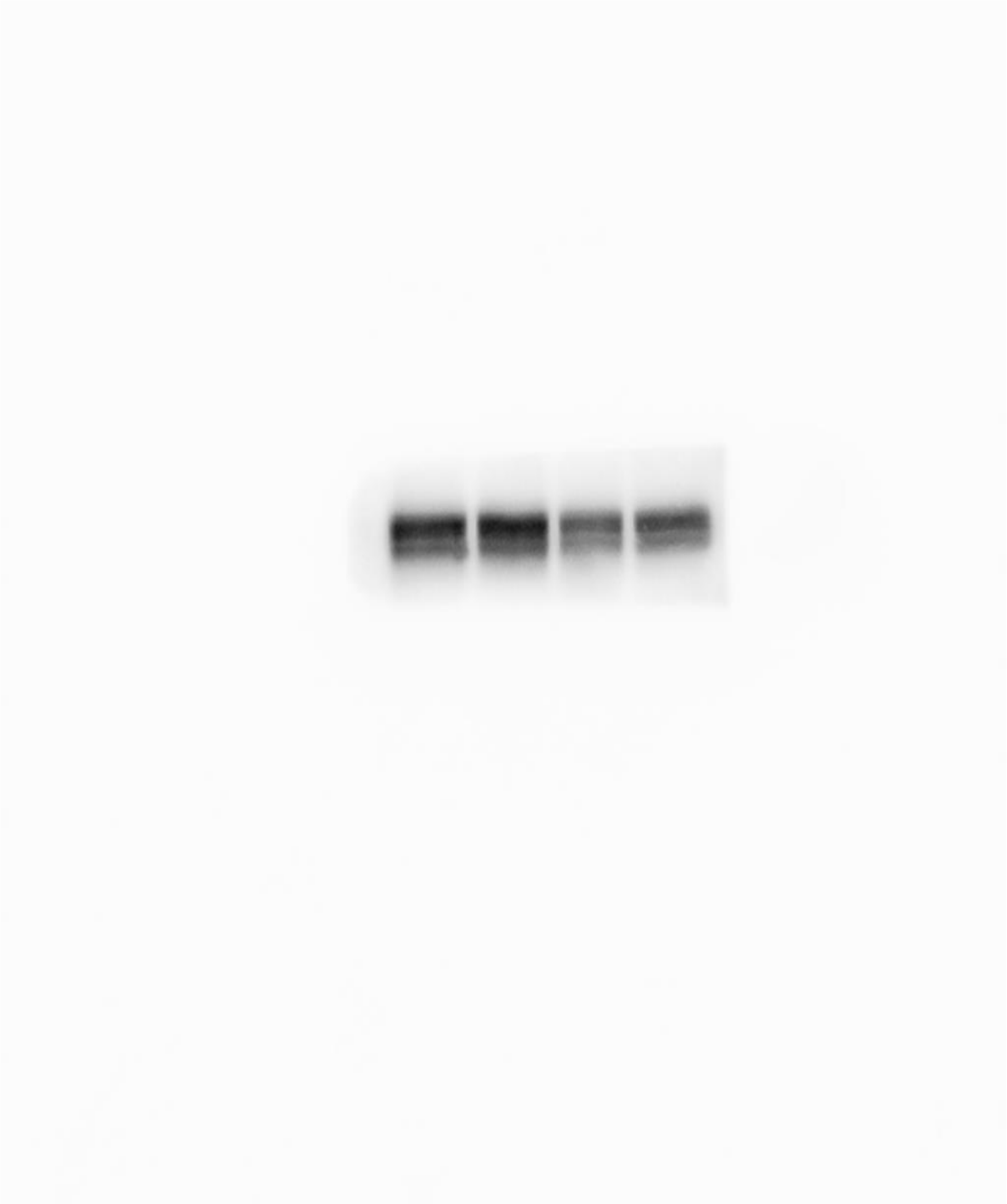

β-ACTIN

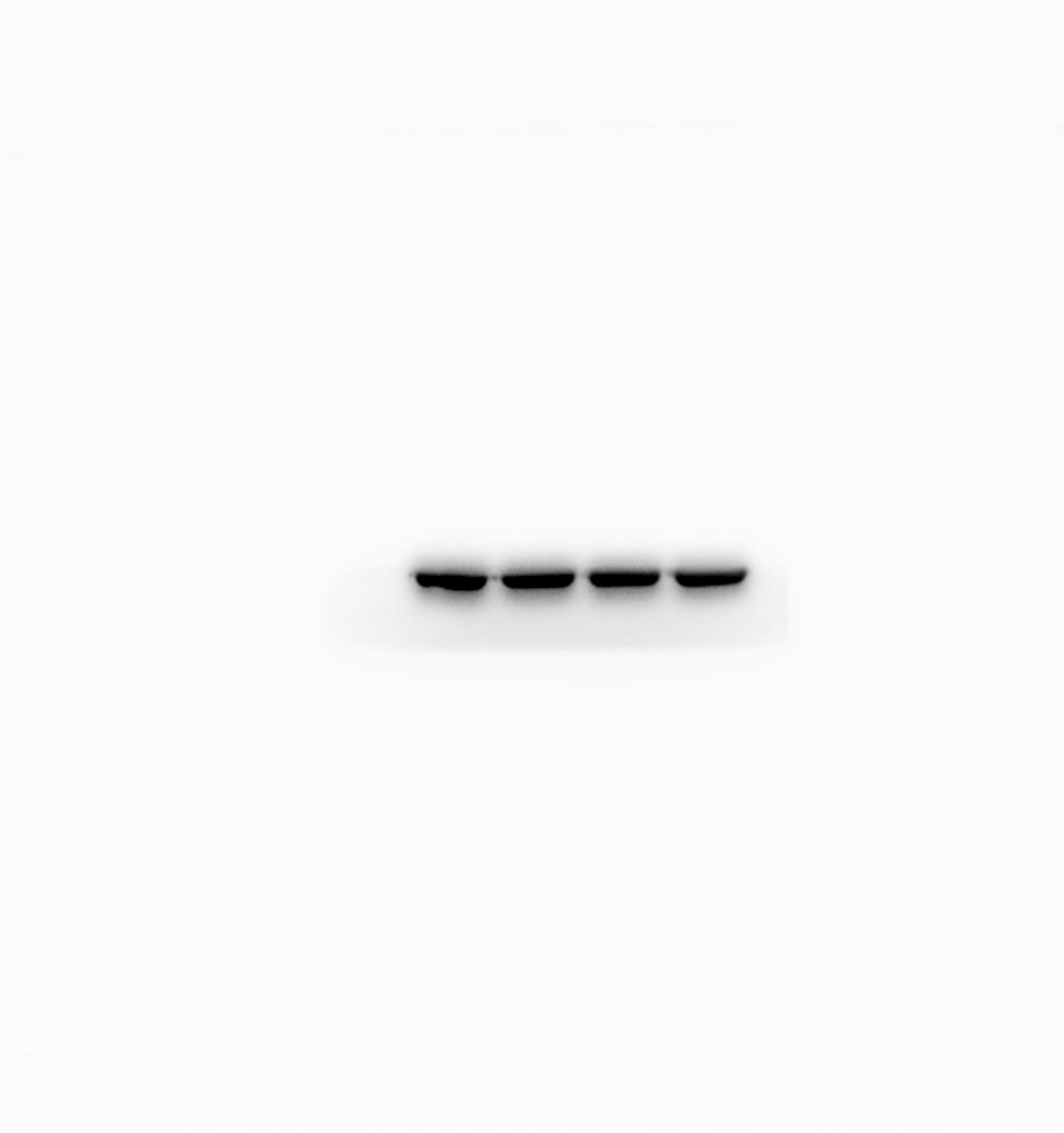

Figure 6_B_RAP_HUH7

HIF-1α

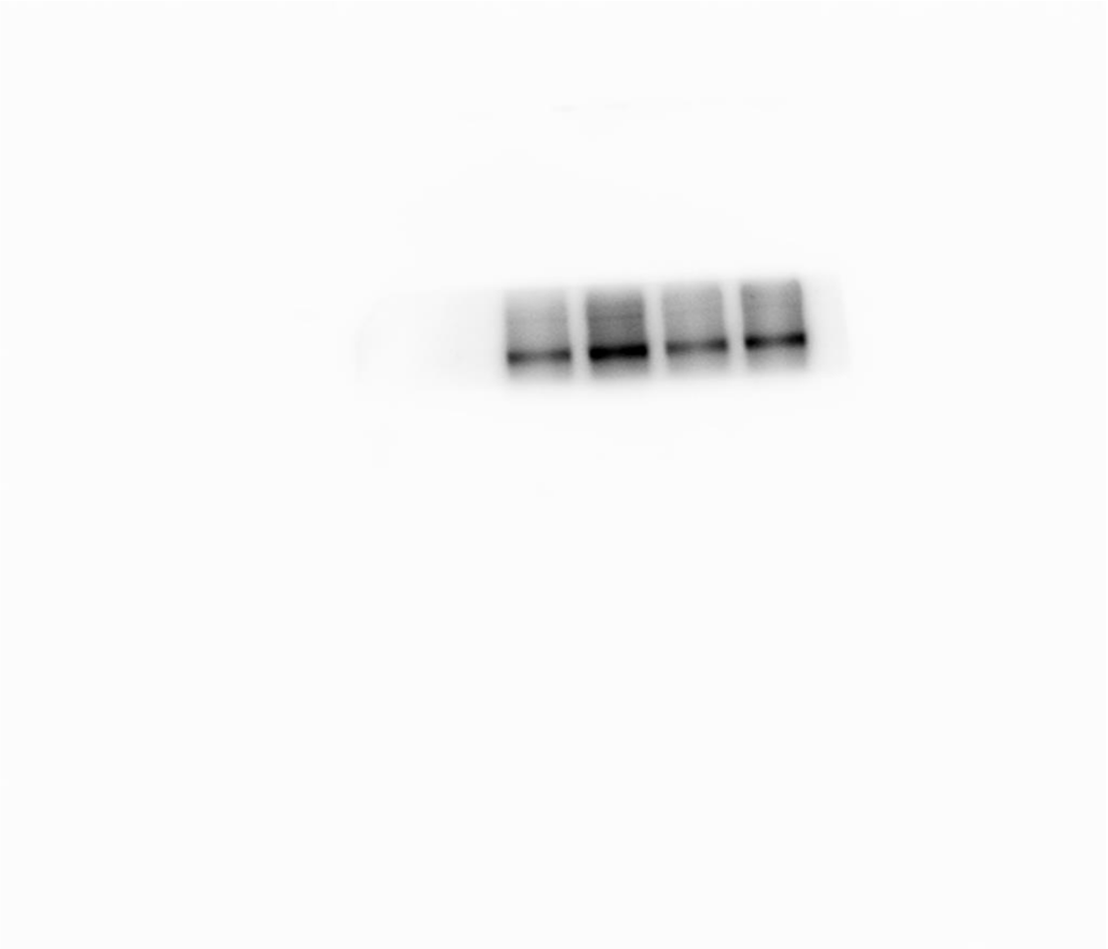

β-ACTIN

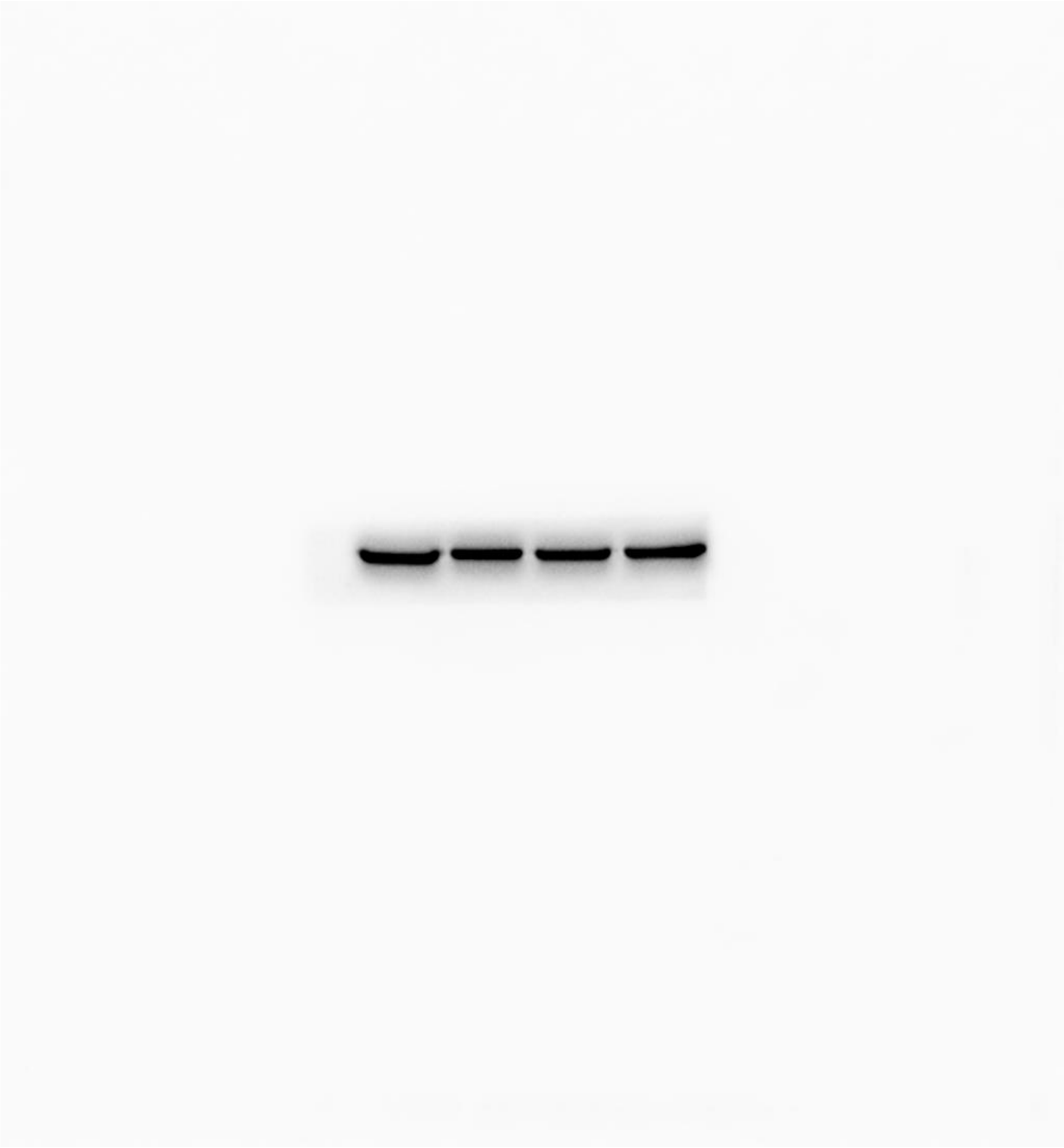

Figure 6_B_RAP_HEP3B

HIF-1α

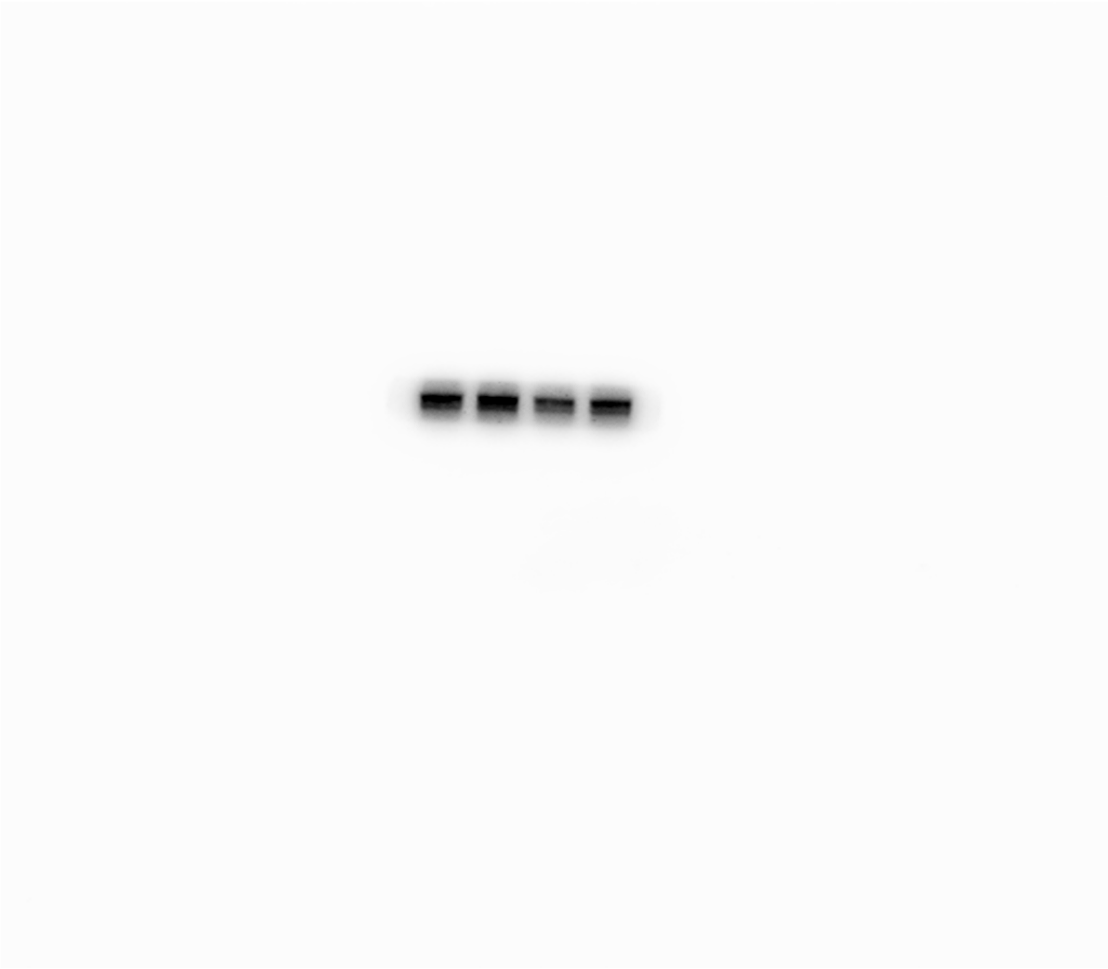

β-ACTIN

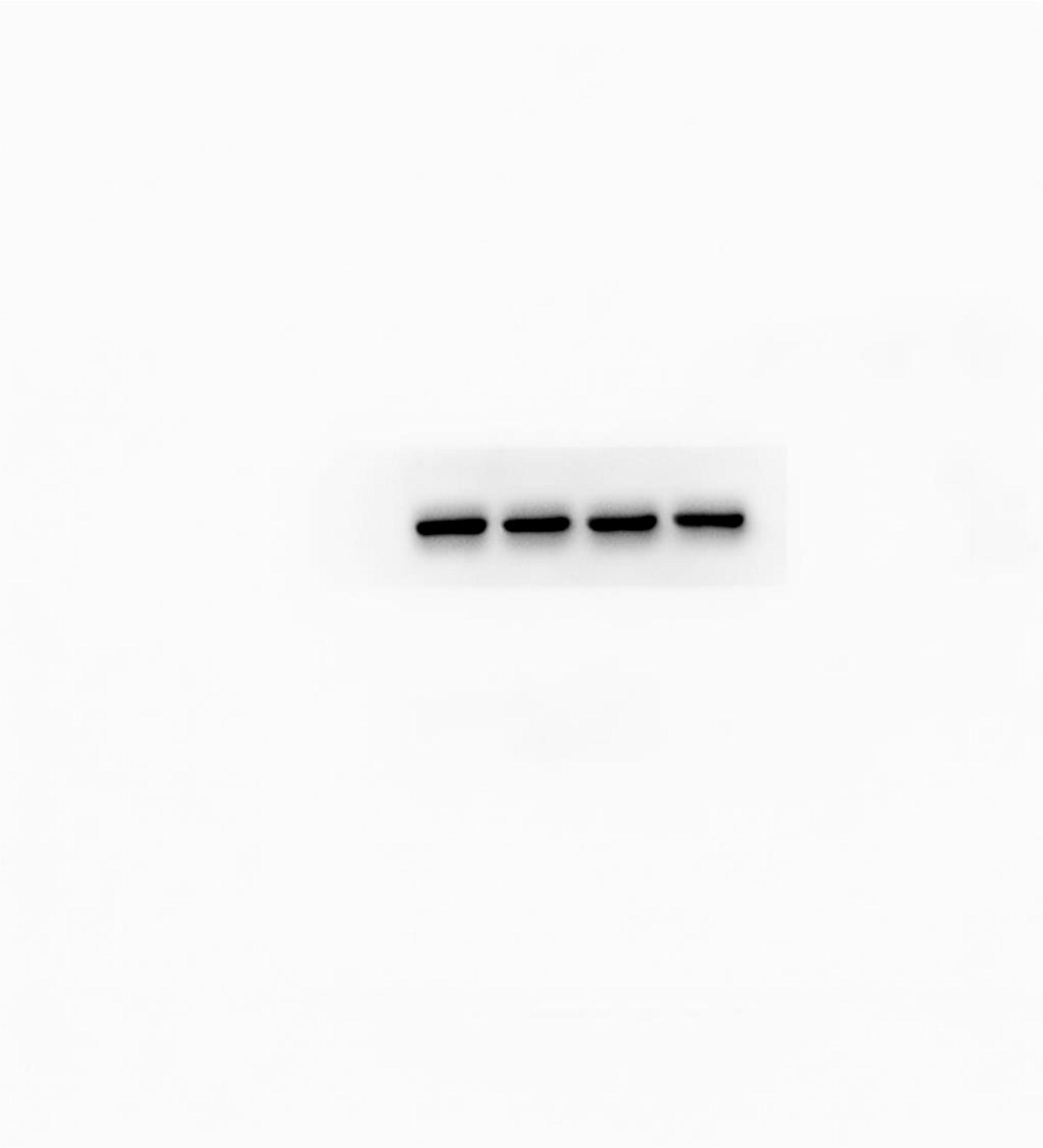

Figure 7_C

SOCS5

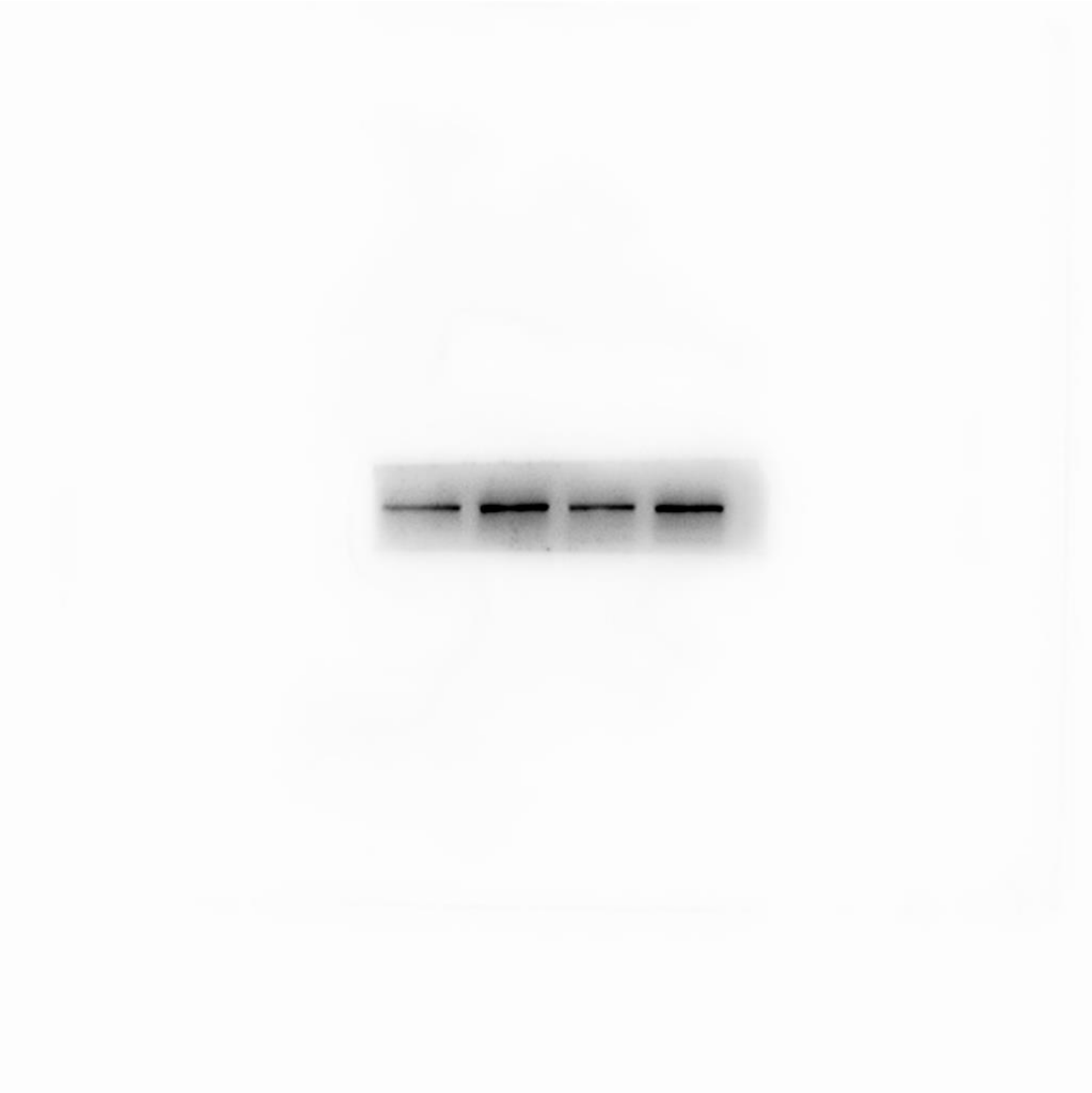

HIF-1α

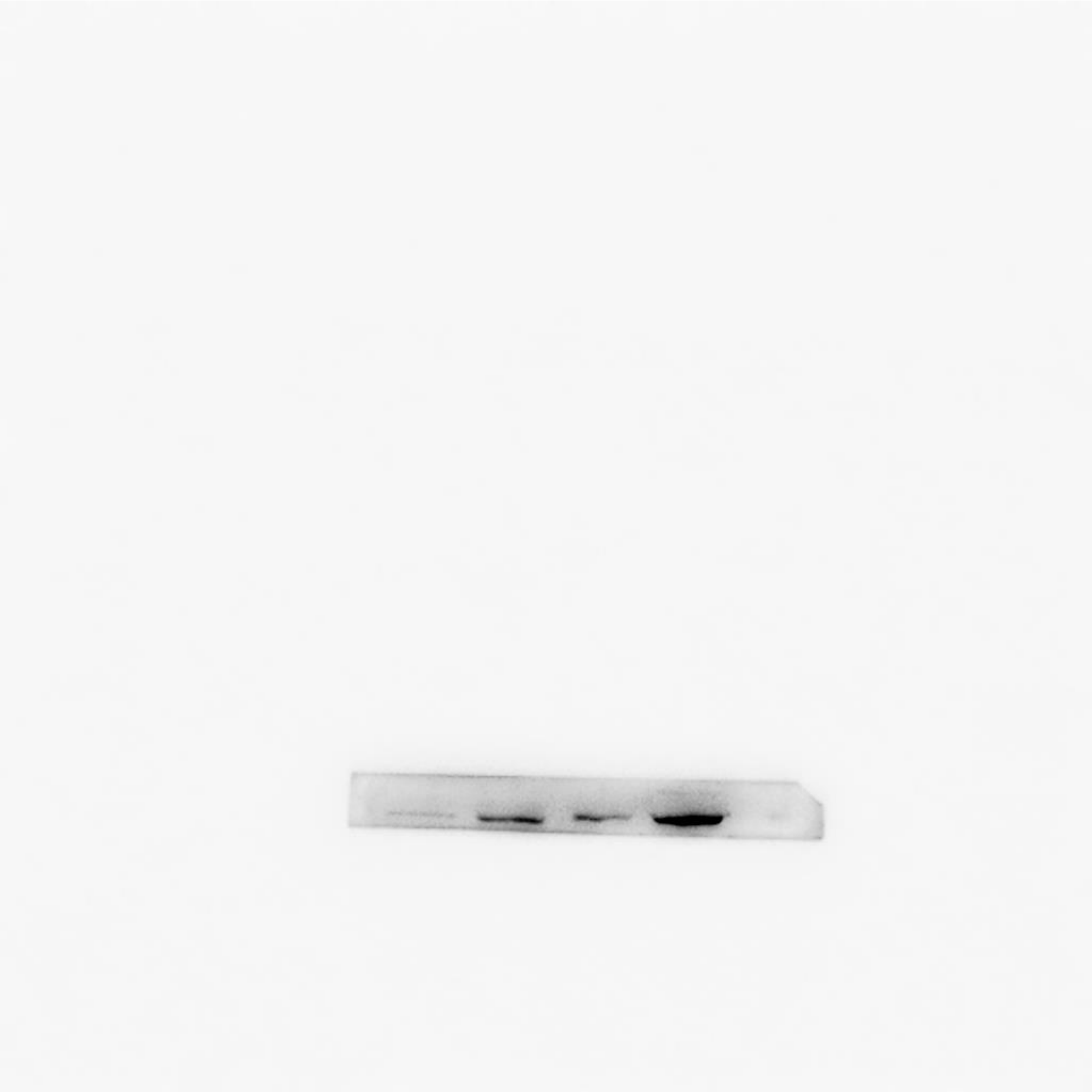

E-Cadherin

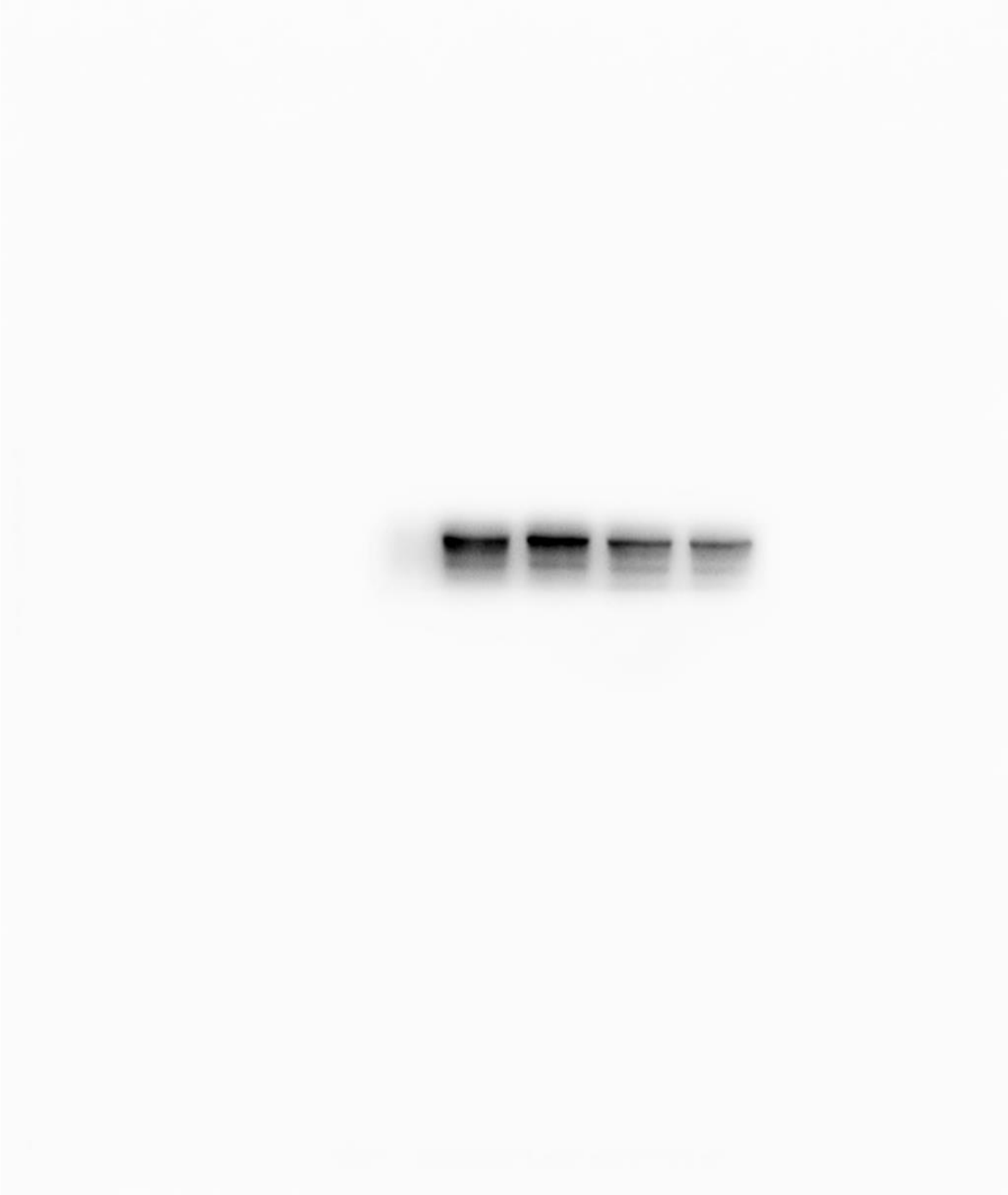

β-ACTIN

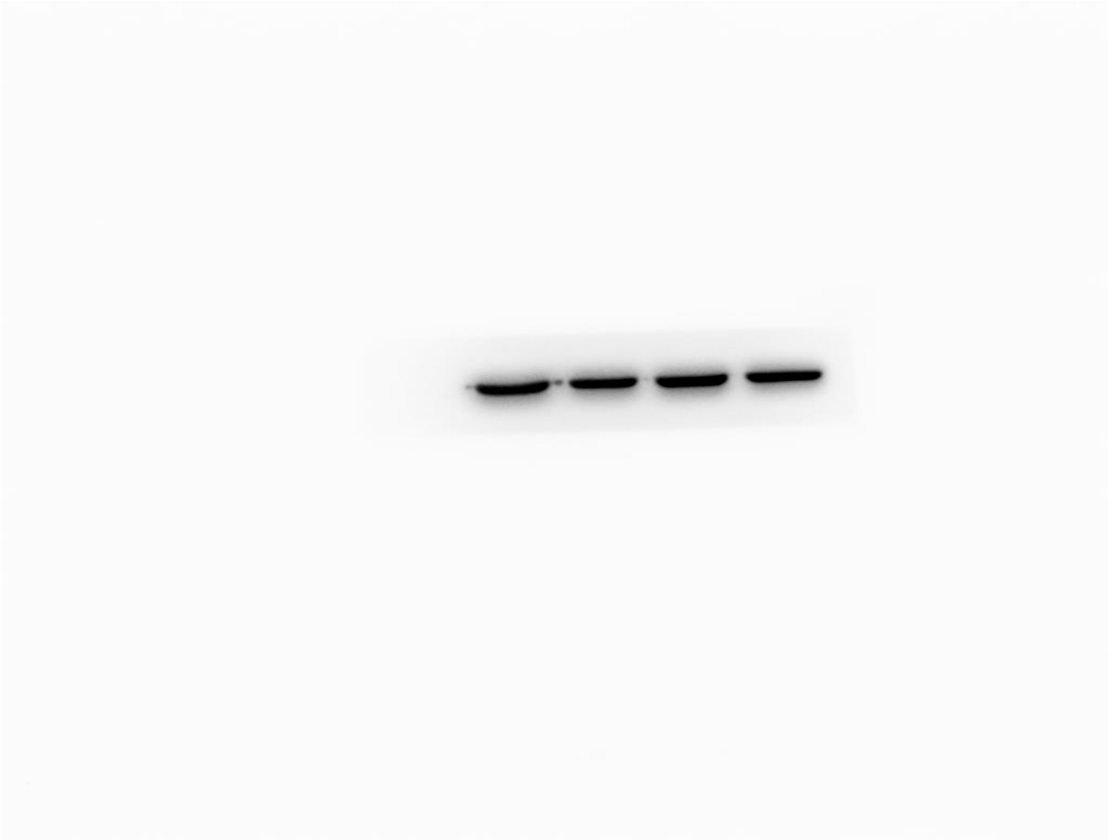

Supplementary Figure S2_B

SOCS5

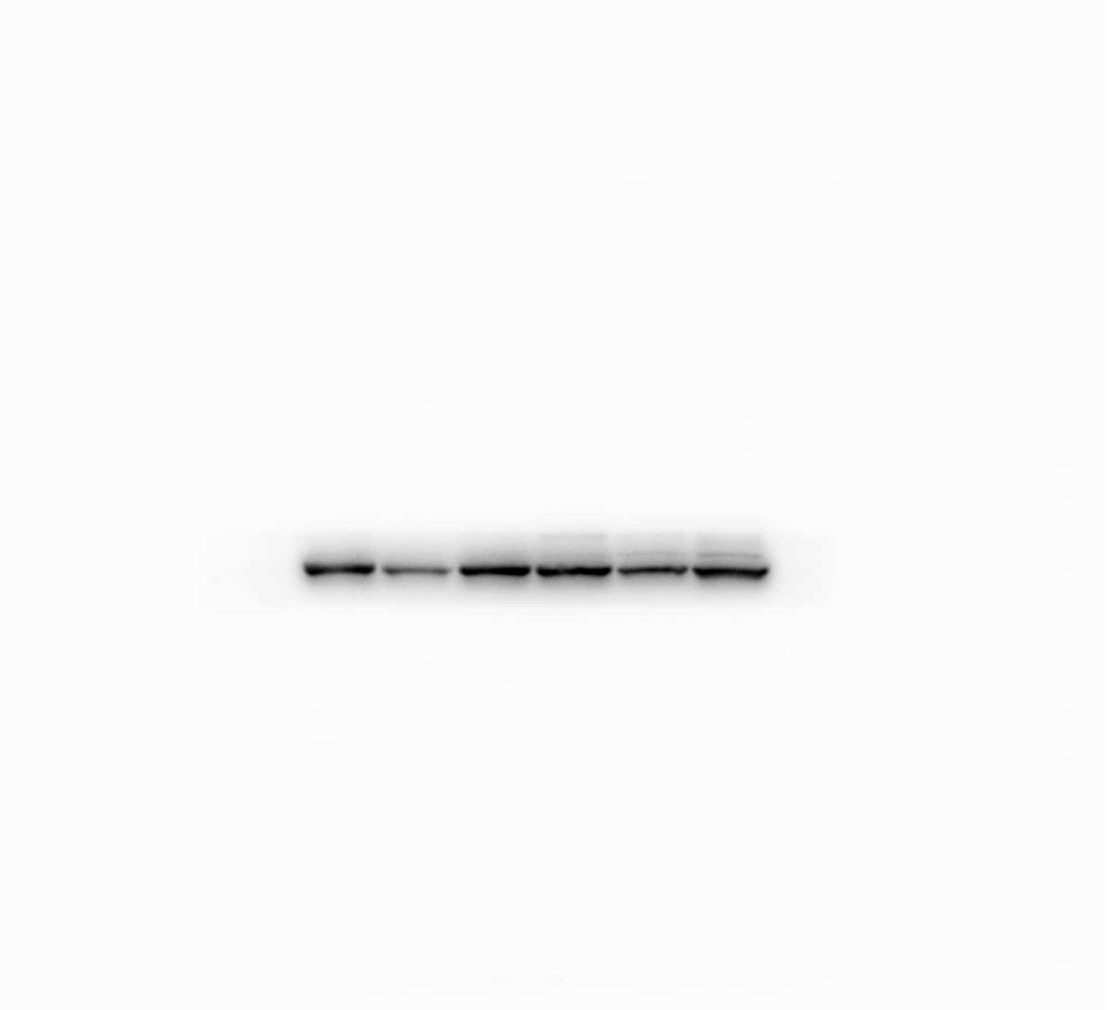

β-ACTIN

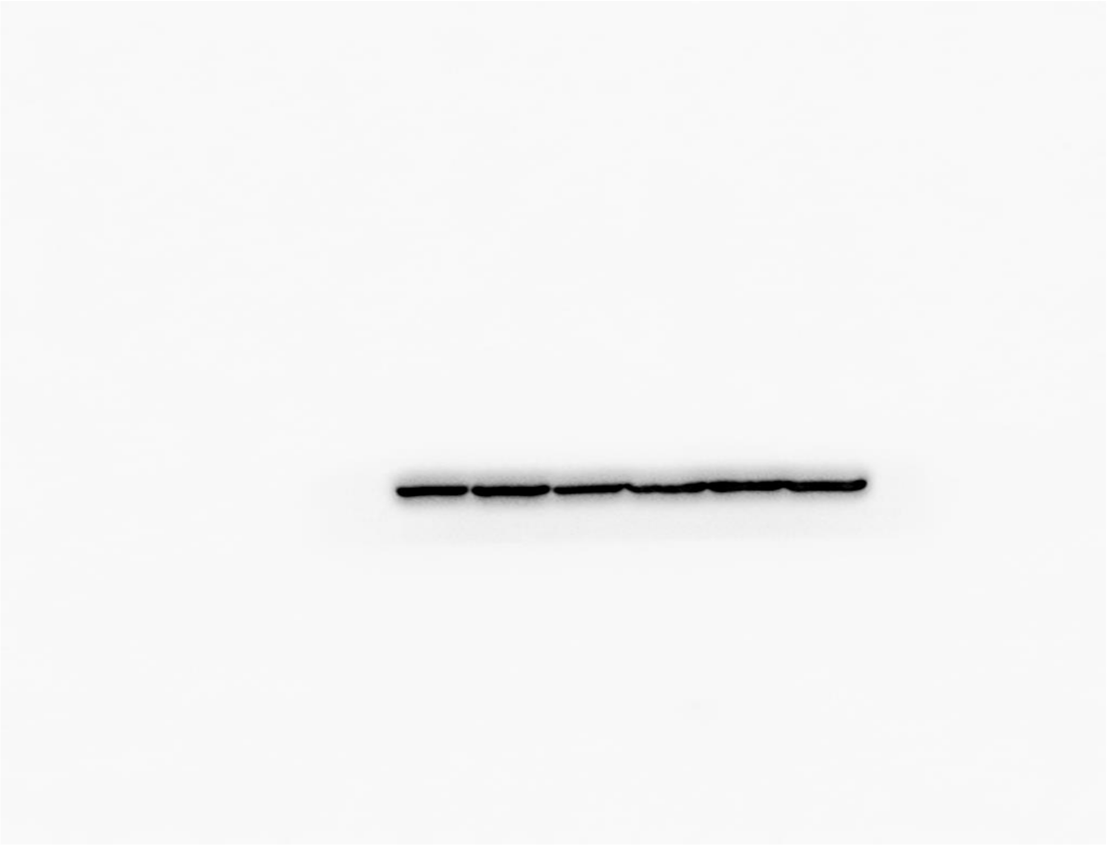

Supplementary Figure S3_C

F-actin

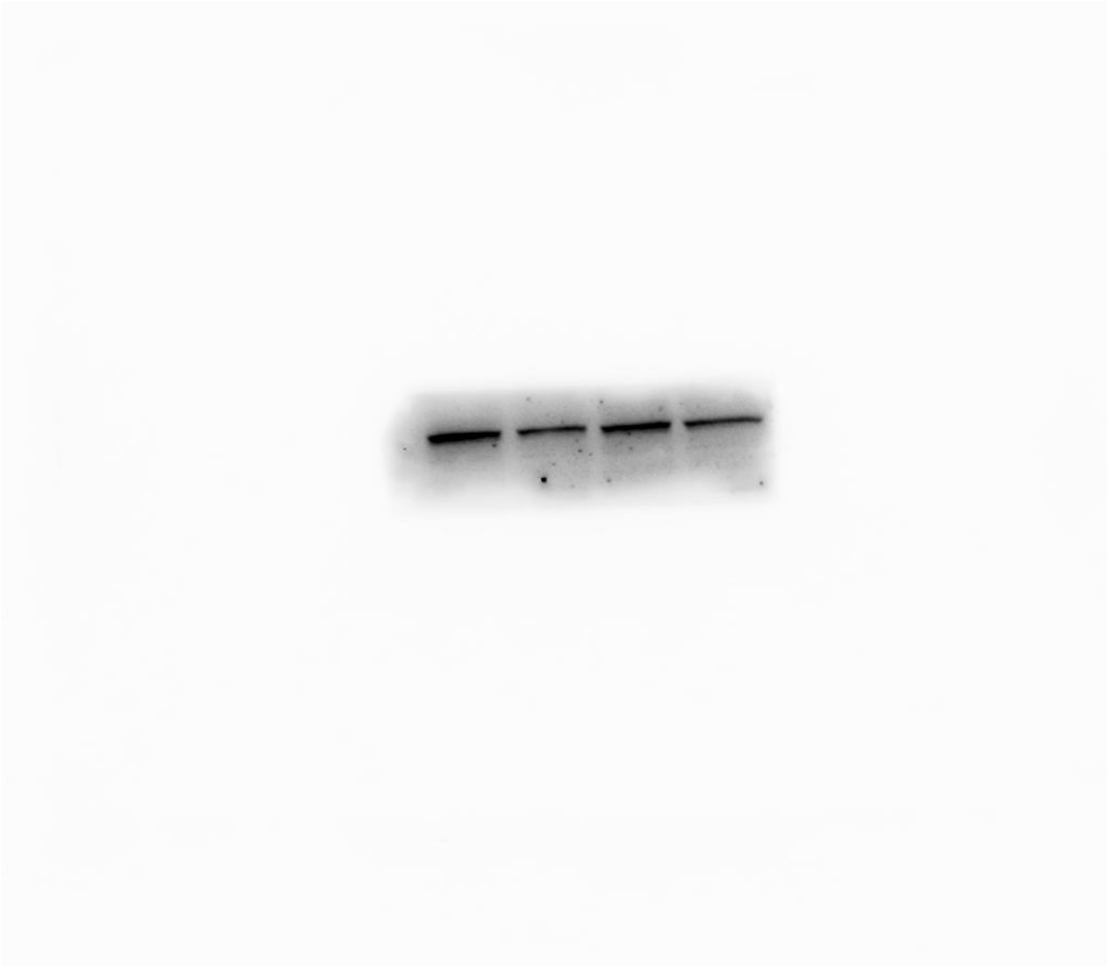

β-ACTIN

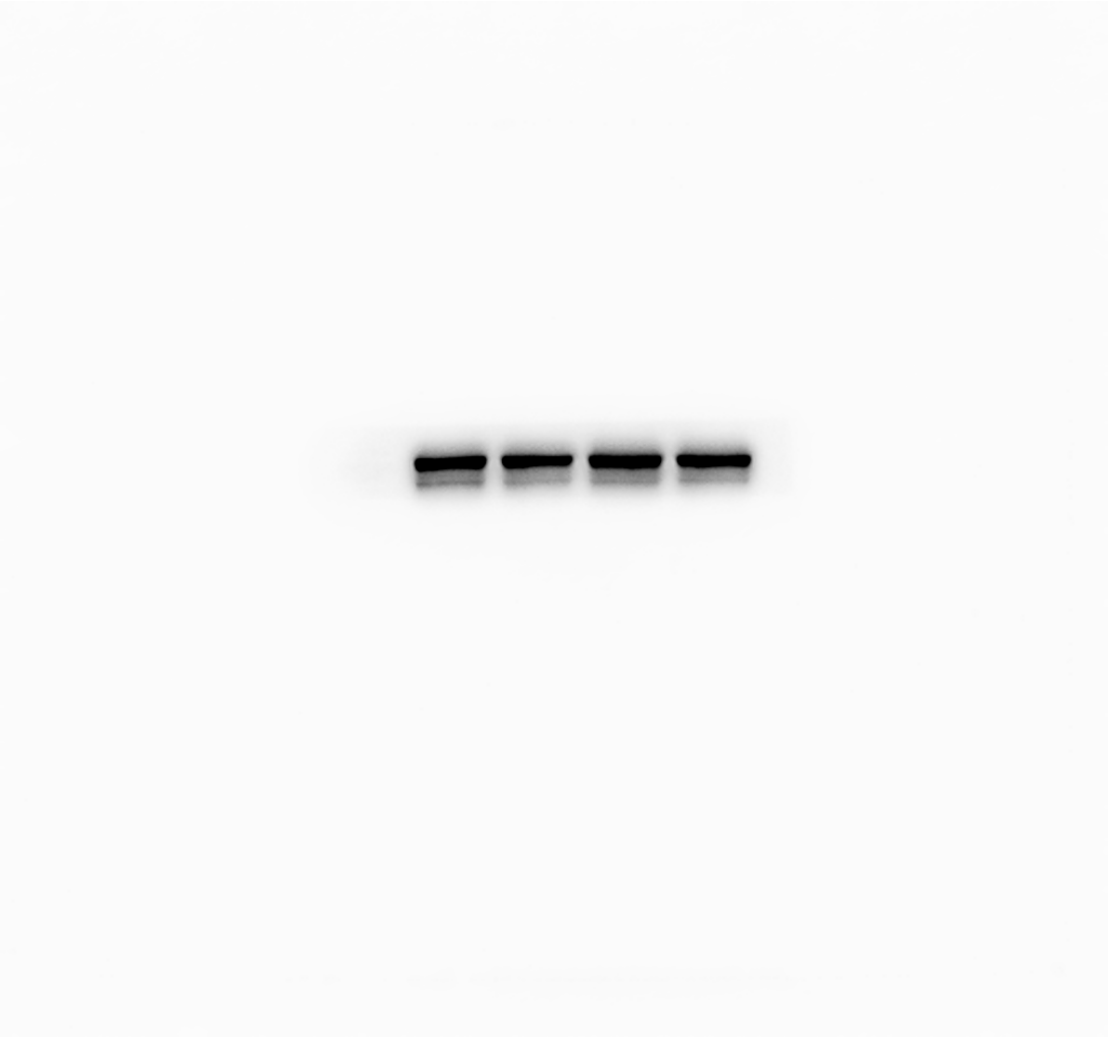

Supplement: Supplementary file 1 — original data files [file 41419_2022_5361_MOESM1_ESM.pdf]
