# Supplementary material for: SOCS5 knockdown suppresses metastasis of hepatocellular carcinoma by ameliorating HIF-1α-dependent mitochondrial damage
Source: Cell Death Dis. 2022 Nov 1;13(11):918. doi: 10.1038/s41419-022-05361-z (PMC9626553; doi:10.1038/s41419-022-05361-z)
Supplement: Supplementary file 2 — Supplementary Figures [file 41419_2022_5361_MOESM2_ESM.docx]

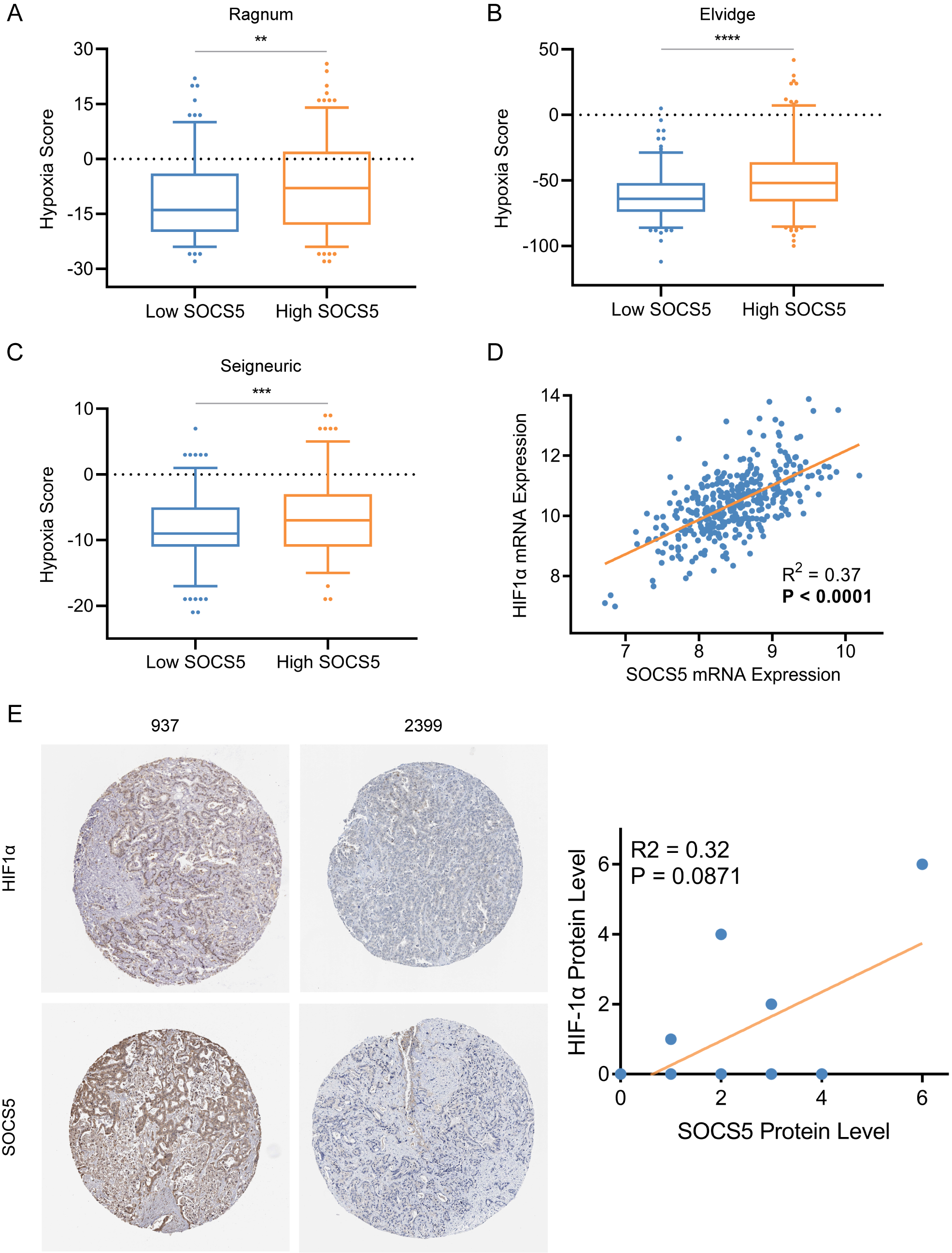


Supplementary Figure S1. *The public database explores the relationship between SOCS5 and HIF-1α.* The high and low expression of SOCS5 was divided according to the median value of SOCS5 mRNA expression in TCGA database, and hypoxia scores of HCC patients were calculated using the hypoxia score formula of Ragnum (A), Elvidge (B) and Seigneuric (C). A positive relationship between SOCS5 and HIF-1α at the RNA and protein level via the cBioPortal website (D) and Human Protein Atlas website (E).


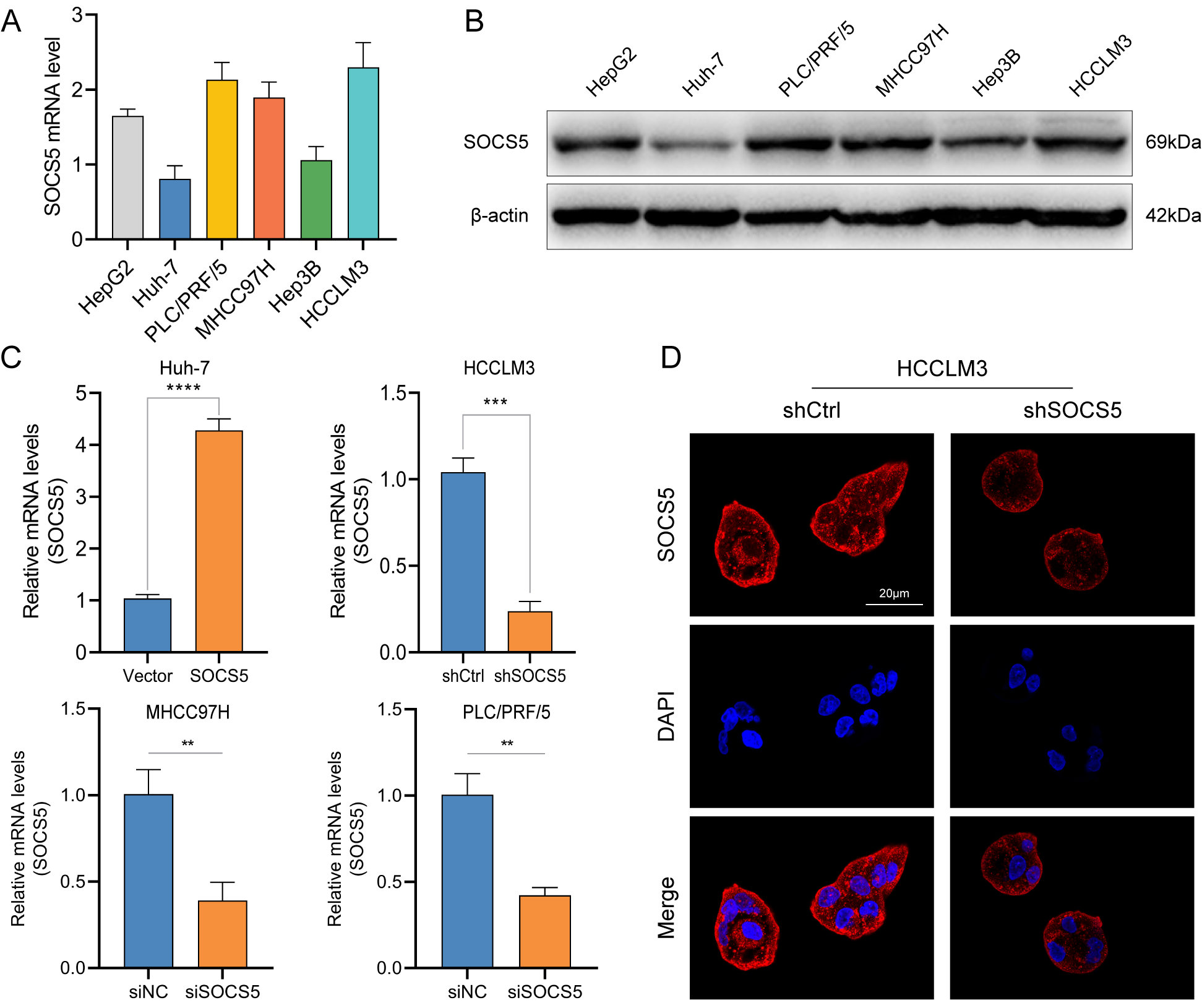


Supplementary Figure S2. A qPCR analysis of SOCS5 basal mRNA expression in six cell lines. SOCS5 mRNA expression levels were normalized according to the GAPDH expression levels. B Western blotting analysis of SOCS5 basal protein expression in the six cell lines. C qPCR analysis of SOCS5 mRNA expression in Huh7, HCCLM3, MHCC97H, and PLC/PRF/5 cells after upregulation and downregulation of SOCS5. mRNA expression levels were normalized according to the GAPDH expression levels. D Immunofluorescence images of SOCS5 in HCCLM3 cells.


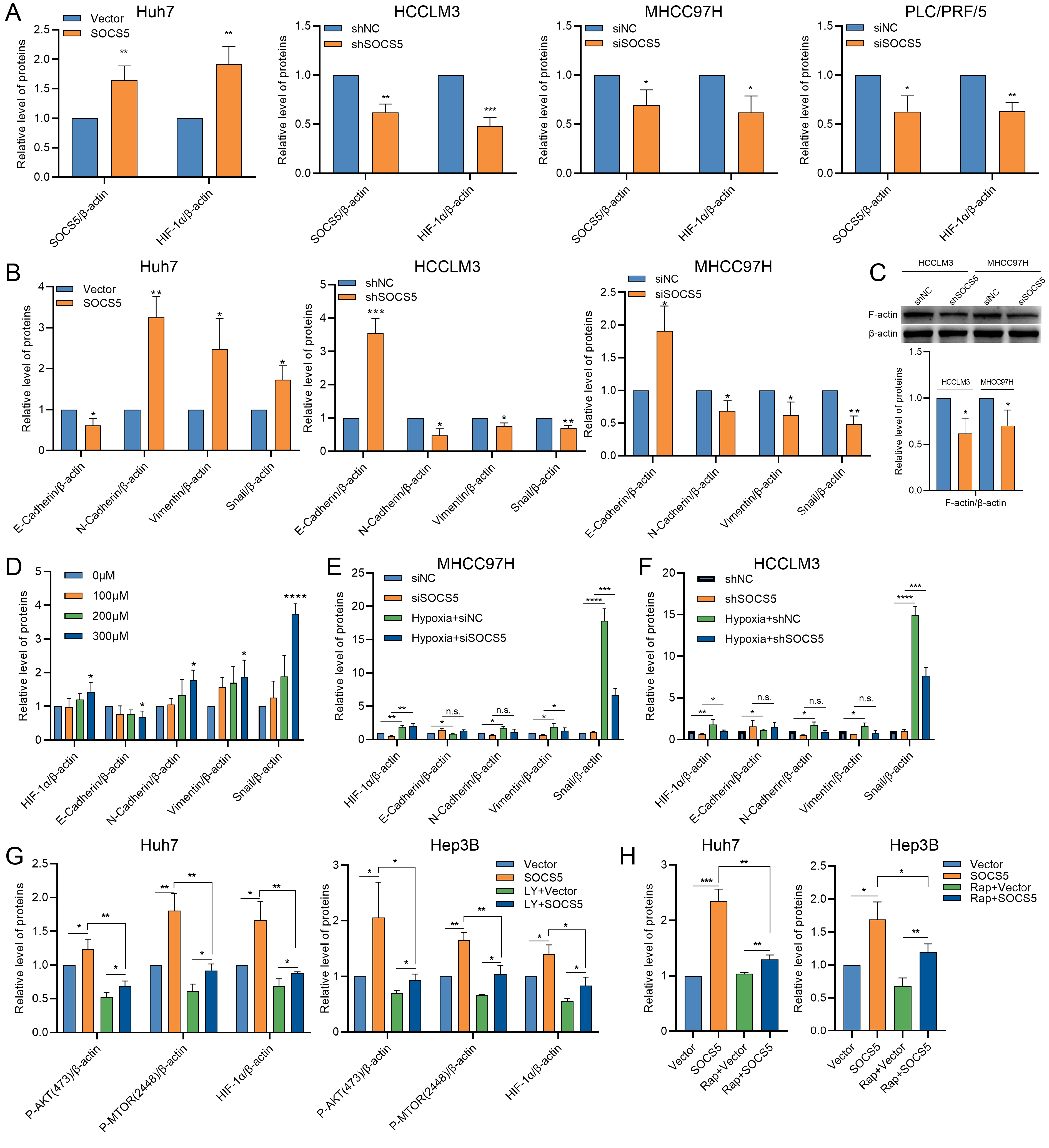


Supplementary Figure S3. A PCR analysis of HIF-1α RNA expression in HCC cells after upregulation and downregulation of SOCS5. B PCR analysis of EMT-related RNA expression in HCC cells after upregulation and downregulation of SOCS5. C Western blot and PCR analysis of F-actin protein and RNA expression in HCC cells after downregulation of SOCS5. D PCR analysis of HIF-1α and EMT-related RNA expression in HCCLM3 cells under hypoxia-induced by Cocl2 (100-300μM). PCR analysis of HIF-1α and EMT-related RNA expression in MHCC97H (E) and HCCLM3 (F) cells. G PCR analysis of p-Akt (Ser^473^), p-mTOR (Ser^2448^), and HIF-1α RNA expression in Huh7 and Hep3B SOCS5-OE-cells treated with 20 μg/mL LY294002 for 48 h. H PCR analysis of HIF-1α RNA expression in Huh7 and Hep3B SOCS5-OE-cells treated with 10 μg/mL Rapamycin for 48 h.
